# Dragonfly‐Inspired Wing Design Enabled by Machine Learning and Maxwell's Reciprocal Diagrams

**DOI:** 10.1002/advs.202207635

**Published:** 2023-04-29

**Authors:** Hao Zheng, Hossein Mofatteh, Marton Hablicsek, Abdolhamid Akbarzadeh, Masoud Akbarzadeh

**Affiliations:** ^1^ Polyhedral Structures Laboratory, Department of Architecture, Weitzman School of Design University of Pennsylvania Philadelphia PA 19146 USA; ^2^ General Office, Department of Architecture and Civil Engineering City University of Hong Kong 83 Tat Chee Avenue, Kowloon Tong Kowloon HKSAR China; ^3^ Advanced Multifunctional and Multiphysics Metamaterials Lab (AM3L), Department of Bioresource Engineering McGill University Montreal QC H9X 3V9 Canada; ^4^ Mathematical Institute Leiden University Leiden 2333CA The Netherlands; ^5^ Department of Mechanical Engineering McGill University Montreal QC H3A 0C3 Canada; ^6^ General Robotic, Automation, Sensing and Perception (GRASP) Lab, School of Engineering and Applied Science University of Pennsylvania 3330 Walnut St Philadelphia PA 19104 USA

**Keywords:** 3D printing, bio‐inspired structures, form and force diagrams, graphic statics, machine learning, structural form‐finding

## Abstract

This research is taking the first steps toward applying a 2D dragonfly wing skeleton in the design of an airplane wing using artificial intelligence. The work relates the 2D morphology of the structural network of dragonfly veins to a secondary graph that is topologically dual and geometrically perpendicular to the initial network. This secondary network is referred as the reciprocal diagram proposed by Maxwell that can represent the static equilibrium of forces in the initial graph. Surprisingly, the secondary graph shows a direct relationship between the thickness of the structural members of a dragonfly wing and their in‐plane static equilibrium of forces that gives the location of the primary and secondary veins in the network. The initial and the reciprocal graph of the wing are used to train an integrated and comprehensive machine‐learning model that can generate similar graphs with both primary and secondary veins for a given boundary geometry. The result shows that the proposed algorithm can generate similar vein networks for an arbitrary boundary geometry with no prior topological information or the primary veins' location. The structural performance of the dragonfly wing in nature also motivated the authors to test this research's real‐world application for designing the cellular structures for the core of airplane wings as cantilever porous beams. The boundary geometry of various airplane wings is used as an input for the design proccedure. The internal structure is generated using the training model of the dragonfly veins and their reciprocal graphs. One application of this method is experimentally and numerically examined for designing the cellular core, 3D printed by fused deposition modeling, of the airfoil wing; the results suggest up to 25% improvements in the out‐of‐plane stiffness. The findings demonstrate that the proposed machine‐learning‐assisted approach can facilitate the generation of multiscale architectural patterns inspired by nature to form lightweight load‐bearable elements with superior structural properties.

## Graphical abstract

This research investigates the use of graphic statics and machine learning to analyze the structural geometry of a dragonfly wing, understand its performance, and generate similar patterns. It can be used in a more universal workflow to generate structural forms inspired from the collected dataset of a wider range of species.

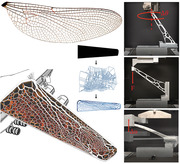

## 1 Introduction

Engineers, scientists, and designers have long been inspired by the morphological and mechanical properties of natural/biological structures and used them in the design of next generation of advanced materials and structures in various scales.^[^ ^2^, ^3^ ^]^ The dragonfly wing is an example of a high‐performance, lightweight structure, providing a repository of design inspirations.^[^ ^4^, ^5^, ^6^, ^7^ ^]^ Various properties of dragonfly wings have been studied previously that may fall into the following main categories: (i) the morphological properties including geometric descriptions and the microstructures of the materials of the veins and surface patches with a focus on the structural properties of the joints;^[^ ^8^, ^9^, ^10^, ^11^, ^12^, ^13^, ^14^, ^15^ ^]^ (ii) mechanical and aerodynamic performance of the wing including the assessment of the behavior of the wing under different loading conditions;^[^ ^5^, ^16^, ^17^, ^18^, ^19^, ^20^, ^21^, ^22^ ^]^ and, (iii) the biological and material properties of the wing particularly in terms of recording the structural parameters of artificial wings under specific loading conditions and the materialization of designs related to advanced materials inspired by the microstructures of the dragonfly wing.^[^ ^4^, ^23^, ^24^, ^25^ ^]^

This research is the first step of multi‐step research on the structural pattern of a dragonfly wing that starts by projecting the 3D geometry of the wing's network on a 2D plane. Valuable research has been conducted on the 3D morphology of the dragonfly wing, including the ridges and valleys of the wing.^[^ ^13^, ^26^, ^27^, ^28^ ^]^ Nevertheless, we deem the 2D pattern of the wing still possesses exciting properties that are yet to be studied and exploited in the design of architected materials or structures. Thus, we limit the scope of this work to 2D networks and intend to shed light on some undiscovered features through this research. In addition, we develop machine‐learning tools to realize alternative bio‐inspired structures that may experience out‐of‐plane bending under uniform pressure and point load or twist. We extrude our networks in the normal direction to create 3D sections to relate the proposed in‐plane geometry to the structural bending performance for potential applications without involving the complexities of the actual folded plate geometry of the dragonfly wing as discussed by other researchers.^[^ ^27^, ^28^ ^]^ The outcome of this research may pave the road for a better understanding of the 3D morphology of the dragonfly wing.

This work builds upon previous research on the morphological aspects of the wing by Hoffmann et al.^[^ ^8^ ^]^ In their research, they divide the internal network structure of the wing into the primary and secondary veins, and provide an approach using a Voronoi decomposition ^[^ ^29^ ^]^ to generate the secondary veins if the primary veins and the boundary geometry of the wing are given. In the current study, we introduce an alternative approach that enables generating similar networks for given boundaries without the need for information about the primary veins.

In the 19th century, mathematicians and engineers investigated the rigidity of frameworks made of iron bars and nodes and their studies led them to the internal static equilibrium of tension and compression forces in the bars. Maxwell in 1864^[^ ^1^ ^]^ proposed a geometry‐based method to calculate the internal static equilibrium of forces. In this proposal, for a given network consisting of closed polygons of bars and nodes a *dual* network is constructed such that each polygon in the first/*primal* network can be related to a node/vertex in the dual. Moreover, each bar/edge of the primal corresponds to an edge in the dual. If the dual diagram is drawn such that each edge is normal to its corresponding edge in the primal diagram, then these two diagrams are *reciprocal*, that is, topologically dual and geometrically perpendicular. The direct application of this approach is in structural analysis, where one diagram represents the structural form while the other represents the forces in equilibrium. The former is called the *form diagram* and the latter is the *force diagram*, which are interchangeable.

Maxwell elucidated the relationship between the reciprocal diagrams as follows: each closed polygon in the force diagram shows the equilibrium of the corresponding node in the structure, and the length of each edge of the force diagram represents the magnitude of the force in its reciprocal members of the structural form. This relationship was the basis of the methods of *Graphic statics* that have been used to find efficient structural forms of cathedrals, bridges, buildings, and long‐span structures for the past 150 years.^[^ ^1^, ^30^, ^31^, ^32^, ^33^, ^34^, ^35^, ^36^, ^37^, ^38^, ^39^, ^40^, ^41^, ^42^ ^]^ This approach has been used to describe the equilibrium of forces in the spider web that consists of convex polygons and it is justified that the spider web can be created from a projection of a polyhedron on a 2D plane.^[^ ^43^ ^]^ We are inspired by this approach and our study begins with the geometric analysis of the network of the veins using a methodology proposed by Maxwell in 1864.^[^ ^1^ ^]^ Maxwell reciprocity has been used to analyze spiderwebs that consist of convex polygons.^[^ ^43^ ^]^ The convex‐dominant pattern of the dragonfly has never been studied using his proposed reciprocity.

**Figure 1:**
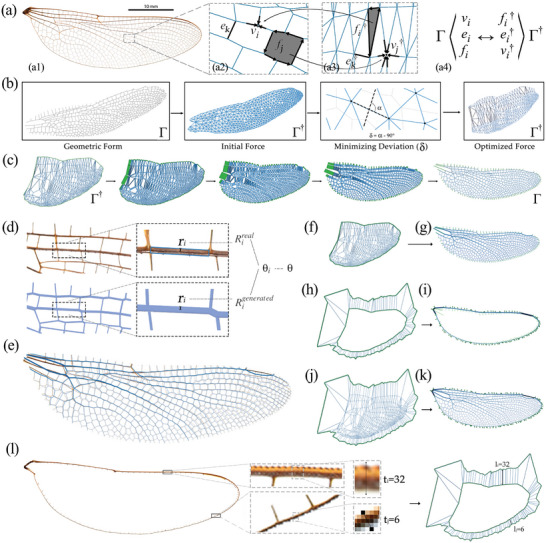
(a1) The structural network of the dragonfly wing: (a2) Maxwell's form diagram of the structure including vertices (*v*), edges (*e*), and faces (*f*); (a3) the dual reciprocal force diagram with its vertexes (*v*†), edges (*e*†), and faces (*f*†); and (a4) the corresponding components of the diagrams; b) a computational flowchart for the construction of the dual from the main network of the wing. c) combining force and form diagram of the wing using Minkowski sum; d) Comparison of the reconstructed form (blue) and the real wing (brown) with structural thickness, *r*: measured radius; *R*: normalized radius; θ: accuracy; θ = 1 − |*R* ^*real*^ − *R* ^*generated*^|; e) superimposition of the reconstructed and the real wing; f–k) combining the determinate force diagram of the main structure and the indeterminate force of the boundaries to complete the geometry of the wing with the boundary edges; l) measuring the pixel width and mapping to the external force diagram.

The structural geometry of the dragonfly wing consists of veins connected by surface patches. The projected geometry of the dragonfly veins or the skeleton of the wing on a 2D plane may represent a structural network made of bars and nodes from which Maxwell's reciprocal diagrams can be extracted (**Figure** **1**a1). Figure 1a2 shows the structural network of the wing and Figure 1a3 shows its dual reciprocal diagram with their related components highlighted in Figure 1a4. Based on Maxwell's reciprocity, the reciprocal dual diagram for the internal network of the wing may represent a possible equilibrium condition for the internal forces in the structure of the wing as follows: each node of the form diagram is reciprocal to a closed polygon of force in the dual diagram. That is, if the internal force in each edge connected to a node matches the length of its corresponding edge in the force polygon then the system will be in equilibrium. The structure's geometry and its reciprocal diagram will provide comprehensive learning data for the machine‐learning model in the later stages of the research.

The equation system for the mathematical representation of the dual diagram is indeterminate and can have infinite possible solutions. To overcome this problem, we divide the geometry of the wing into two parts by separating the internal network from the boundary edges. This means that the system of equations to solve for the dual can also be divided into the equations related to the main network and the equations pertaining to the boundary edges and their nodes. The key observation is that the internal network of the dragonfly wing has a unique dual/reciprocal diagram up to scaling (Figure 1b). In fact, the force equilibrium in the projected network of the structure of the wing excluding the boundary edges mainly consists of triangles representing a geometrically determinate network. Based on this dual diagram, we can conclude that the internal structural skeleton of the wing is a statically determinate system (Figure 1b). Visualizing the internal forces as diameters in the members of the wing provides a thickened network of the wing as illustrated in Figure 1c.

A surprising observation is that the network with member diameters defined by the geometric equilibrium of forces matches the 2D projection of the real structure of the wing with its member diameters with an acceptable range of tolerance (Figure 1d,e). This observation has four major outcomes: (1) the internal network of the wing has a unique dual reciprocal diagram that can show a determinate equilibrium of forces in the network providing additional learning data for machine learning together with the geometry of the wing; (2) the location of the primary and secondary members of the real wing matches the reconstructed structural network made by graphic statics; (3) the primary members in the form of the wing corresponds to a particular region in the force diagram including force triangles with larger edge lengths; this information is quite useful in the regeneration of the wing and the location of potential primary and secondary members; and (4) the determinate solution for the dual graph of the main network can be used to find a dual diagram for the boundary members by matching the diameters of the real wing and the static force equilibrium in the nodes (Figure 1f–k).

Before proceeding to the following stages of this research, the authors would like to state the following disclaimer. Undoubtedly, the mentioned observation does neither imply that the entire structure of the cellular core of the wing is in static equilibrium nor does it say that the static equilibrium is sufficient to describe the behavior of cellular core of wing or its loading conditions. We acknowledge that the biological and environmental loading conditions, including sophisticated loading patterns that led to the formation of the wing's geometry over the years, are quite complex, as it has already been investigated.^[^ ^44^, ^45^ ^]^ Nevertheless, our observation provides researchers with additional insight that the morphological configuration of the veins may suggest an in‐plane, static equilibrium of forces as a possible force equilibrium in the system. Furthermore, the geometric representation of forces can be used for the regeneration of lightweight structural design solutions for various boundaries inspired by the geometry of the wing; in this paper, we present selected experimental and computational studies on the structural performance of the 3D printed dragonfly‐inspired cellular cores that may be used in aeroplane wing or meta‐sandwich structures in load‐bearing structural applications.^[^ ^46^ ^]^

**Figure 2:**
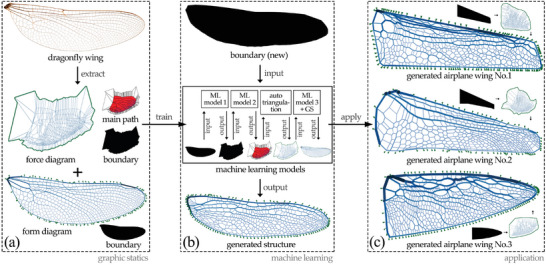
Workflow of this research: graphic statics, machine learning, and application. We propose to: a) extract the main features of the dragonfly wings using graphic statics; b) train machine learning models using the form and force diagrams to generate dragonfly wing structures; c) implement the trained models from step (a) to generate a determinate network for given boundary conditions of an airplane wing structures.

We further use machine learning methods to generate the structural networks by inputting the form and force diagrams of the dragonfly wing as the training dataset for the generation process (**Figure** **2**a). We identify the main regions in the force diagram that includes triangles with similar edge lengths and area. These regions correspond to the primary and secondary veins of the wing. We then reconstruct these regions using machine learning and triangulate those regions to get force polygons that complete the force diagram (Figure 2b). Subsequently, a vector‐based machine learning model is used to construct the determinate structural network of the wing as a dual for the regenerated force diagram. This machine learning model predicts edge lengths for the structural network that is close to the actual geometry of the wing. In this process, we use the force diagram to ensure that the resulting structural network generated by machine learning is in static equilibrium (Figure 2c).

The novelty of this work is to replicate the structural patterns of some natural species using reciprocal diagrams of graphic statics and machine learning methods. The out‐of‐plane performance of the dragonfly wing in nature motivated us to test this research's real‐world application in designing structures for airplane wings as a cantilever structure. Specifically, we investigate dragonfly‐inspired designs on the 1:120 scale, 2D extruded airframe of the Boeing 777 wing and could observe the improvement of the structural efficiency of the wings from the perspective of stiffness and weight. In this regard, the out‐of‐plane stiffness of the wing with respect to point load and distributed air pressure has been studied. Numerical and experimental results depict dragonfly design can improve out‐of‐plane stiffness by 25%, leading to lighter designs. In the following sections, we will provide detailed explanations of the methodology and findings of this research.

## 2 Results and Discussion

The following sections present the results of this study based on our proposed methodology. In this regard, our investigations and the related outcome can be divided into the following steps: (1) generating the geometry of the force equilibrium using reciprocal diagrams for the main network of the wing; (2) reconstructing the network from the calculated force diagram; (3) adding boundary members of the main network and constructing a determinate reciprocal diagram for the entire wing; (4) training machine learning models using the geometry of the force diagram to generate wing networks for a given boundary geometry; (5) designing, and numerically and experimentally testing the cellular core of airplane wing structures using our proposed method; and (6) applying the same methodology to generate networks for other species with convex networks.

### 2.1 Constructing a Reciprocal Diagram for the Main Network of the Wing

The member connections on a dragonfly wing include (T), (Y), and (+) shaped connections.^[^ ^47^ ^]^ Since the (T) joints in the projected network may represent zero‐force members, we adjusted the network slightly and changed them to (Y) joints. Thus, we assumed in this study that the main network of the dragonfly wing includes convex‐only polygons so that we can solve for its dual diagram. This assumption inherently induces some deviations from the network's original geometry, which is insignificant.

Figure S3 (Supporting Information) shows the process of the data preparation. The original dragonfly wing image is transformed into a vector‐based geometry with convex and non‐convex polygons using image processing techniques of edge detection in OpenCV^[^ ^48^ ^]^ (Figure S3a,b, Supporting Information). The non‐convex polygons are slightly adjusted to make convex polygons by an algorithm that iteratively updates the coordinates of the vertexes (Figure S3c,d, Supporting Information).

A dual reciprocal diagram for the geometry of the wing can be constructed as proposed by Maxwell.^[^ ^1^ ^]^ To achieve this goal, we call the dragonfly network the *form diagram* (Γ) that represents the form of a structural network and includes the length of members, and the locations where members are connected to the boundaries. Respectively, its reciprocal dual is the *force diagram* (Γ†) that consists of closed polygonal faces and can show an equilibrium configuration of forces in each node of the form diagram. These two diagrams are reciprocal, meaning that the vertices, edges, and faces of one diagram correspond to the faces, edges, and vertices of the other diagram. Moreover, the edges of the two diagrams are perpendicular. Given a form diagram, there can be infinitely many reciprocal force diagrams that represent the static equilibrium of forces in the members of the form diagram. The dimension of the possible dual networks is called the Geometric Degrees of Freedom (GDoF) of the dual network.^[^ ^49^ ^]^ Indeed, the degrees of static indeterminacy in the form diagram is equal to the GDoF of the force diagram. In the case of the internal network of a dragonfly wing, the dual network mainly consists of triangular faces forcing its GDoF to be one. This means that the force diagram is unique up to scaling and the form diagram of the wing is *statically determinate*.

To construct the unique force diagram for the internal network of the dragonfly wing, we use iterative methods developed in ^[^ ^50^ ^]^ (see Section A.4, Supporting Information). Each polygon *f*_*i*_ in the form diagram is transformed into a vertex $v_{i}^{†}$ in the force diagram, and all the adjacent vertices are connected by an edge (Figure S4a,b, Supporting Information). This gives the dual graph of the form diagram, which includes the topology of the force diagram with incorrect edge lengths. Then an iterative method is applied to adjust the position of each vertex $v_{i}^{{†}^{\prime }}$ and make its connected edges perpendicular to the edges of the polygon *f*_*i*_ (Figure S4c, Supporting Information). In this approach, in each step of the iteration, the edges of the force diagram are rotated to become perpendicular to the edges of the form diagram. The difference measured from the right angle 90° and the angle of the two corresponding edges α is defined as the deviation δ. The iteration process minimizes the value of δ to derive a solution of the force diagram within a predefined tolerance (Figure 1b).

### 2.2 Reconstructing the Wing's Main Network from its Reciprocal Dual Diagram

Once the force diagram is constructed for the original network, we can use it to reconstruct a new network of the wing. The dual relationship between these graphs allows us to indefinitely build one from the other using the iterative method of.^[^ ^50^ ^]^ Note that although the force diagram is geometrically determinate, its reciprocal dual, that is, the network of the wing, has GDoF larger than one. In fact, for the same force diagram, there are infinite reciprocal form diagrams that can be constructed with different edge lengths, all representing a statically‐determinate network in equilibrium (See section A.5, Supporting Information). Besides, the magnitude of the forces in the members stays intact, as only one force diagram represents the equilibrium. Therefore, to get the closest possible solution to the dragonfly wing, we need the original network's edge lengths and use them to reconstruct a statically determinate network similar to the wing. This edge length information will be used later in the machine‐learning process for regeneration purposes. The average deviations of the angles of corresponding edges for the form‐to‐force and the force‐to‐form processes are 2.64° and 0.64°, respectively.

With the force diagram, the members of the new structural network can be sized based on Maxwell's reciprocity: the magnitude of the force in each member of the structure is proportional to the length of its dual edge in the force diagram. We found that the ratio of the minimum and maximum edge lengths in the force diagram is approximately 1:10. We also measured the thickness of the real dragonfly wing in pixel, the minimum and maximum widths are approximately 6 pixels and 60 pixels. Thus we size the members of the generated network using the force diagram.

We assume a circular section for the generated network to compare with the real wing since the outer shape of the cross‐section of the actual members is circular.^[^ ^51^ ^]^ Note that the cross‐section of the real members is not uniform,^[^ ^52^ ^]^ and for this research, we only compare the outer diameter of the real and the generated members. First, the maximum and minimum diameters of the members of the real wing are measured. Then, the force diagram is scaled to match the maximum diameter (projected cross‐section in 2D) of the members in the real wing. Subsequently, the edges of the new form diagram are sized based on the magnitude of the force by relating them to their corresponding edge in the force diagram. Next, we compared the member thicknesses in the real and the generated form. Our results show a 91.9% match (Figures S8 and S9, Supporting Information), which concludes that sizing the members in the real wing may follow the static equilibrium of forces in the determinate graph of the wing. This sizing can be visualized by combining both form and force diagrams into a single diagram using Minkowski sum ^[^ ^41^ ^]^ as illustrated in Figure 1c.

### 2.3 Completing the Boundary Edges

As briefly discussed, finding the design forces in nature for which the wing has evolved to perform optimally is a challenging task. We also mentioned that finding a dual diagram for the entire wing network, including the boundary edges, is a complex problem since the network has many dual solutions. Nevertheless, we showed that the internal network of the wing, as a graph with bars and nodes, is statically determinate and has a unique dual diagram. The combination of the internal network and the boundary edges make a statically *indeterminate* system.

We have shown that the internal network of the wing with the member sizes proportional to the edge lengths of the reciprocal diagram matches the members in the real wing. We can use these results as deductive reasoning to find the thickness of the external members and complete a determinate dual diagram. As illustrated in Figure 1l, we introduce a set of external loads at the periphery of the wing called *virtual forces*. We find these loads such that if applied to the boundaries, they would produce the same internal forces proportional to the diameters of the members in the real wing. To find the magnitude of such forces, we measure the diameter of the actual boundary members and solve for an external loading that can create such internal forces in those members proportional to their diameter. Then, a separate force diagram is constructed for the boundary members and combined with the determinate force diagram of the internal network of the wing (Figure 1f–k). The resulting combined dual diagram is determinate and can be used to describe the geometry of the entire wing. Note that the entire wing geometry represents an indeterminate system, and our dual diagram can present an equilibrium condition among others where the internal forces match each member's diameters.

Figure 1e shows an example of the comparison of the generated dragonfly wing and the real dragonfly wing by measuring the radius of each member (Figure 1f). The structural thickness is predicted with an accuracy of 92.5%. Then, we proceeded with another 24 pieces of dragonfly wings. The results of all cases show an accuracy higher than 88.1%, while the overall average accuracy is 92.0%. The comparison of the reconstructed and the real dragonfly wings are illustrated in Figure S14 (Supporting Information), and the related statistics are shown in Table S2 (Supporting Information). These initial results lay the basis for the second phase of this research, which is related to the work's generative use of machine‐learning algorithms with no information on the initial topology of the network. The determinate force diagram of the wing with its convex polygons includes Information about the primary and secondary veins and their diameters. Thus, it will be used as valuable data to learn from and apply its design logic to similar cases.

### 2.4 Generating Structural Networks using Machine Learning Models

**Figure 3:**
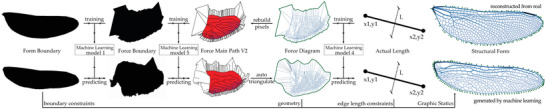
The Workflow of GAN+ANN models with automatic process to reconstruct the force geometry.

In the following phase, we develop a method using machine learning models capable of generating the entire structural form of the wing from a user‐input boundary with an intermediate product of the force diagram (**Figure** **3**). First, two image‐based machine learning models are trained, first of which generates the force boundary from the form boundary, and the rest generates the force main path from the force boundary. Then, an algorithm is developed to reconstruct the force diagram as vectors from the force main path image. Last, a vector‐based machine learning model is trained to predict the length of the corresponding edge in the form diagram for each edge in the generated force diagram. Therefore, the final structural form can be generated from the force diagram and the predicted length values by graphic statics method.

To build the training and testing dataset for the image‐based models, geometries in different stages are transformed into images (see Section A.9, Supporting Information). Inspired by the identification of the main veins by,^[^ ^8^ ^]^ a similar method is developed to extract the main path of the force diagram of the dragonfly wing (Figure S16, Supporting Information). In the main path image, the R channel with a value of either 0 or 255 represents the existence of regions of each main path, the B channel with a value of either 0 or 255 represents the existence of the middle point of each edge in each region, and the G channel represents the length of the corresponding edge in the force geometry if the value in the B channel shows an existing middle point in that pixel. If the values in the R, G, and B channels are all 0, the pixel is regarded as the Black channel, which represents the boundaries of the main path. Therefore, this force main path can store both the pixel‐based information of the boundaries of the main path, and the vector‐based geometric information of the force lines. Finally, a dataset of 25 pieces of dragonfly wings is collected, in which the first 21 pieces are used as the training set and the remaining four pieces are used as the testing set. To increase the size of the dataset, we apply image augmentation techniques to rotate the training images with ‐15, ‐10, ‐5, 0, 5, 10, and 15 degrees, thus there are 147 image pairs in the training set.

Then, since the data is in the format of images and each image is generated one by one in a clear order of an input and an output, image‐to‐image machine learning models called Generative Adversarial Networks (GANs) ^[^ ^53^ ^]^ are used to learn the mapping between each stage of the dragonfly wing data. Besides, to automatically generate the force geometry from the force main path, we develop a method that triangulates the regions in the predicted main path according to the recognized vertexes (Figure S19, Supporting Information). To be specific, the R channel is first separated and the skeleton geometry is extracted. Second, the middle points are recognized from the G and B channels. The main path boundaries are extracted from the black pixels, from which the external force geometry is reconstructed. Next, the internal vertexes are inferred from the skeleton geometry, middle points, and the main path boundaries. And finally, the internal triangulation is generated based on the internal vertexes, and the entire force geometry is combined from the internal and the external force geometries. The final geometry should be a series of closed polygons that together becomes a convex network to meet the requirement of our graphic statics method.

However, the force diagram can only represent the topological information of the structural form; the geometric information, including the edge lengths for the form diagram, is still missing. Therefore, another vector‐based machine learning model of Artificial Neural Network (ANN) ^[^ ^54^ ^]^ is proposed to predict the edge lengths of the form diagram using a dataset of the edge lengths extracted from the dragonfly wing geometries (see Section A.10, Supporting Information). It inputs the coordinates of the start and end points and the length of an edge in the force diagram, and outputs the length of the corresponding edge in the form diagram (Figure S20, Supporting Information). To be specific, the dual diagram of the force geometry is first generated by the graphic statics method. Then for each edge in the dual diagram, a vector (*x*_1_, *y*_1_, *x*_2_, *y*_2_, *f*) is generated, which represents the coordinates of the start and end points and the force magnitude (length for the corresponding edge in the force diagram). In addition, the corresponding edge length in the real form is found as the output of the ANN model. After the fine‐tuning of the hyperparameters and the training process, the ANN model can predict the actual edge length for each edge in the dual diagram, thus helping generate the structural form using the graphic statics method. Therefore, the machine learning models can predict all information needed to generate the structural form of the wing. This GAN+ANN workflow can first generate images of force diagrams, then extract the geometric information, and finally, predict the length constraints to generate the form diagrams.

### 2.5 Validating the Results

**Figure 4:**
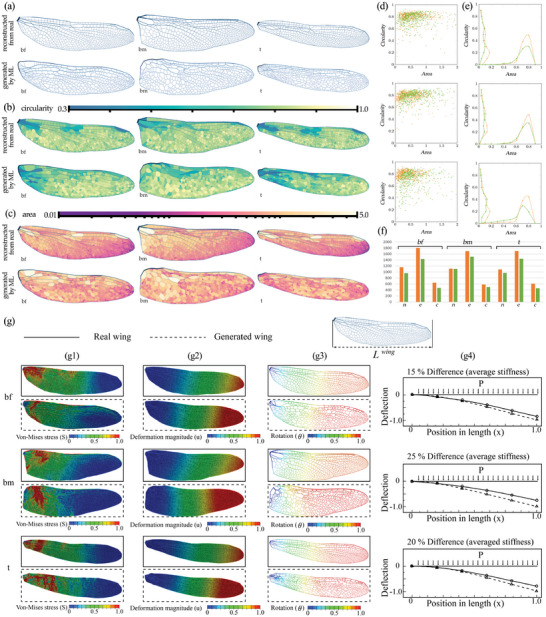
a) The real and the generated wings. The explanation of naming: b/t: bottom/top; m/f: male/female. (The virtual loads are not shown.) b) The visualization of the circularity measure on the wing polygons. c) The visualization of the area measure on the wing polygons. d) The plot of the area and the circularity of each polygon for the real wing (orange) and the generated wings (green). Top: sample bf; Middle: sample bm; and Bottom: sample t. e) The smooth curve of the area and the circularity of each polygon for the real wing (orange) and the generated wing (green). Top: sample bf; Middle: sample bm; and Bottom: sample t. f) the statistics of the numbers of nodes/vertices (v), edges (e), and faces (f), for the real wing (orange) and the generated wing (green). g) Mechanical properties of the real and generated dragonfly wings: (g1) Von‐Mises stress, (g2) Magnitude of displacement, (g3) Rotation, contour, and (g4) Deflection under a uniform pressure (values are normalized with respect to the maximum value found for the real‐wing).

To test the performance of the overall workflow, a testing dataset with three categories of dragonfly wings is used, including top and bottom wings for male dragonflies and bottom wings for female dragonflies. Figure S21 (Supporting Information) shows the samples of the three categories, in which the top wings of male and female are the same, while the bottom wings are different. Thus, the testing dataset contains the input (form boundary) and the output (structural form) of dragonfly wings in these three categories. **Figure** **4**a shows the comparison of the real and the generated dragonfly wings in the three categories.

To compare the similarity of the wing pairs, the evaluation method based on the area and the circularity of each cell in the wing pattern ^[^ ^8^ ^]^ is implemented. They represent the morphological features of each cell, and the statistics can show the overall similarity of the entire pattern. The area is computed from the geometry of the cell, while the circularity is computed by the ratio of the area of the cell to the area of a circle with the same perimeter of the cell. Figure 4b,c shows the visualization of the two measures, while Figure 4d,e shows the plot diagrams. In the scatter plot (Figure 4d), the scatters for the real and the generated dragonfly wings are generally close and overlapped. For the cells with similar areas, the circularity of the generated dragonfly wings is higher, while for the cells with similar circularity, the area of the generated dragonfly wings is higher. This phenomenon might be caused by the deviation in the graphic statics when generating the form diagram from the force diagram. The deviation makes the angles in the cells of the form diagram shifted compared with the designed angles in the force diagram. Meanwhile, the distribution map (Figure 4e) also proves the above observation. But generally speaking, the generated dragonfly wings are similar to the real dragonfly wings. We also determine mode shape, natural frequencies, and the stiffness of a real wing and a generated one under uniform pressure; numerical results demonstrate high similarities for the response of the two cellular patterns (Figure S41, Supporting Information). Our method successfully generates the structural form of the dragonfly wing from its form boundary.

To compare the ML‐assisted generated wing with the actual dragonfly wing, the average out‐of‐plane displacement of three wings under uniform pressure has been studied. In this comparison, the thickness of the generated wings is bio‐mimicked to be very similar to the actual wing. The adopted design methodology is capable of generating vein patterns, therefore veins are modeled accurately in the simulation. Although the patches enclosing the in‐plane space between veins may have different thicknesses, for a fair comparison we assume an average value for all patches ($\frac{t^{patch}}{L^{wing}}=4\times 10^{-4}$, *t* ^*patch*^ and *L* ^*wing*^ are patch thickness and wing's length, respectively). Results in Figure 4g shows that the out‐of‐plane stiffness of generated wings differ by about 20% from the real wings, which are acceptable since the similarity of structural properties was not the objective of the ML‐assisted design methodology. By analyzing stress contours, it can be understood that patches are experiencing more stress at the left part of the wing, which can be a part that initiates larger passive deformation in the wing. This is more obvious in the rotation contour that the right part of the wing has rigid displacement (uniform color from the middle to the tail). The deformation contour depicts that by mimicking thicknesses we can achieve a similar deformation pattern but softer in the generated wings.

### 2.6 Designing an Airplane Wing using the Proposed Method

**Figure 5:**
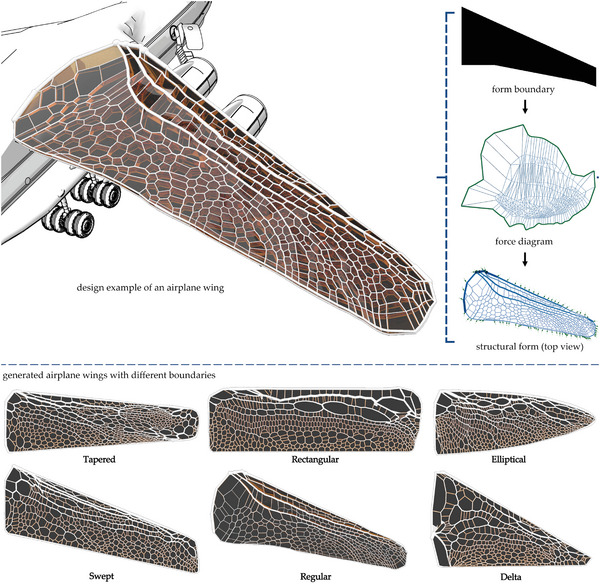
A broader application of generating an airplane wing using our method.

In the broader application of engineering and design fields, the design of a nature‐inspired structure can be achieved using our method. With the trained models, we can input the human‐defined boundary and ask the machine learning model to generate the structural form within the boundary. **Figure** **5** shows one of the applications of designing an airplane wing. The loading scenarios of the dragonfly wing and the airplane wing have some common similarity, that both bear the lifting force from bottom to top. Therefore, features from the dragonfly wing can be transformed to design an airplane wing. Besides, we learn from the dragonfly that the design needs a larger external force in the front compared to smaller external forces in the back. The generated force diagram matches this principle that the internal forces in the top are larger than that in the bottom. Also, the structural form contains similar features such as the main paths as the dragonfly wing. In addition, by modifying the 2D force diagram into 3D geometries with forces represented as inclined closed polyhedrons, the overall curvature in the section can be created, which matches the cross‐section of a real airplane wing. Figures S26 and S27 (Supporting Information) present more generated airplane wings with different boundaries.

Airplane wings are among structures that require to be stiff and lightweight simultaneously. Even a small improvement in achieving a higher stiffness‐to‐weight ratio leads to a considerable reduction of material and fuel consumption in the aerospace sector. For a long time, airplane wings are fabricated with straight ribs. Advances in manufacturing have enabled the fabrication of advanced materials and structures with complex architecture from nanoscale macroscale.^[^ ^7^, ^46^, ^55^, ^56^, ^57^, ^58^, ^59^, ^60^, ^61^, ^62^, ^63^ ^]^ However, obtaining a very stiff design for the airplane wing is a crucial challenge. We cannot guarantee to achieve the global‐optimum point in the cellular core of the wing design.^[^ ^64^, ^65^, ^66^ ^]^ In the current study, we utilized the developed ML‐assisted design methodology to introduce dragonfly‐inspired structures as the core of the airfoil wing, assuming that the dragonfly's wings generate a great lift force while being lightweight and stiff.^[^ ^45^, ^67^ ^]^

**Figure 6:**
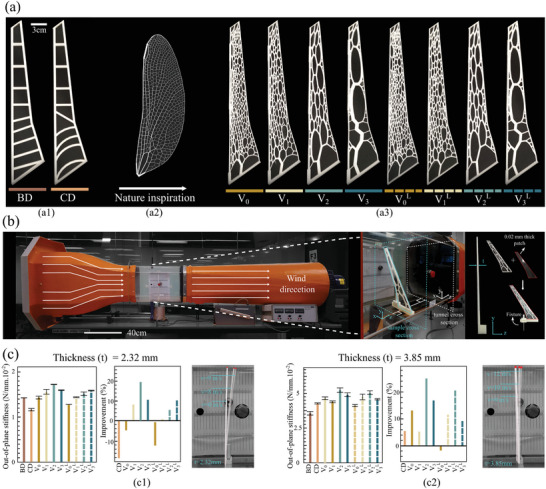
a) 3D printed samples for conventional designs (BD and CD) and dragonfly‐inspired designs (*V*_0_ to *V*_3_ and $V_{0}^{L}$ to $V_{3}^{L}$ L3), b) Wind tunnel setup; 3D printed samples are covered with 0.02 mm stencil sheets for conducting the test, and c) Out‐of‐plane stiffness properties of the 3D printed samples with (c1) 2.32 mm thickness and (c2) 3.85 mm thickness under uniform air pressure.

We generate four dragonfly structures with alternative subdivision densities correlated to their architectural features (i.e., number of constitutive struts), namely *V*_0_, *V*_1_, *V*_2_, and *V*_3_ designs (**Figure** **6**a). Besides, we have considered a minimum thickness limitation in our design to reflect the constraints of additive manufacturing technologies for the 3D printing of small features. Adopting 0.6 mm as the minimum strut thickness, manufacturable by common fused deposition modeling (FDM), we have also designed four samples (i.e., $V_{0}^{L}$, $V_{1}^{L}$, $V_{2}^{L}$, and $V_{3}^{L}$) with manufacturing constraints. Exploring the microstructure of the dragonfly wing demonstrates the existence of mainstream of thick struts (main veins) in the front of the wing. Interestingly by reducing subdivision density, the mainstream is getting more pronounced, while applying manufacturing constraints to the design vanishes the mainstream. Samples tested under point loads have height and weight of 7.8 mm and 30g, and those tested in the wind tunnel have a height of 2.32 and 3.85 mm and weight of 9g and 14.8g, respectively. All samples are designed with the same volume fraction of 0.38.

Mechanical properties studied here for the wing structure are out‐of‐plane, torsional, and in‐plane stiffness and maximum strength. Due to the importance of out‐of‐plane stiffness, it has been evaluated under both point load and uniformly‐distributed load (wind pressure). Torsional and in‐plane stiffness have been measured under point load. The maximum strengths of the 3D printed samples, made out of polylactic acid (PLA), is evaluated under out‐of‐plane two‐sided clamped deformation and tail rotation. ADMET eXpert 8612 axial‐torsion testers were used to carry out the out‐of‐plane, torsion, and in‐plane tests. SONY DSC‐RX100M4 camera was used to capture the videos of the experiments (more information can be found in Figures S42–S70, Supporting Information).

Initially, the developed bio‐inspired designs are studied under a simplified load case (i.e., point load) to better understand the effect of geometrical constraints, minimum beam width, and subdivision density on the structural performance of dragonfly‐inspire cellular core of the wing designs. For the baseline, two wing core patterns have been chosen. The first traditional design with straight ribs has conventionally been used in designs of aeroplanes, and the curved rib design has recently been suggested to improve the performance of the wing structures.^[^ ^64^ ^]^ This study compares all results to the wing with the traditional pattern (straight ribs) as a baseline. The experimental results for structural stiffness evaluated under a point load condition indicate that dragonfly‐inspired designs can improve out‐of‐plane and torsional stiffness of the wing compared to the basic design (BD), especially for $V_{2}^{L}$ and $V_{3}^{L}$ (Figure 6b; Figure S66a, Supporting Information); however, the basic design performs very well under an in‐plane loading condition. It is found that reducing the number of features and adopting the minimum thickness limitations improve the strength of the structure, which may emanate from manufacturing defects for samples with high subdivision density and very thin struts (Figure S65a, Supporting Information). In addition to improving the strength of $V_{2}^{L}$ and $V_{3}^{L}$ designs compared to BD, the ultimate structural failure section occurs at the tip of dragonfly designs (Figure S66b, Supporting Information). It is worth mentioning that airplane wings in service are under a distributed load and are clamped to the airplane fuselage, leading to maximum shear force and moment at the fixed supports. Therefore, failure at the tail found in the dragonfly‐inspired designs is an advantage compared to the failure at the front of the basic and curved rib designs. Finite element studies on the bio‐inspired designs also reveal a similar trend for the out‐of‐plane, torsional, and in‐plane stiffness of the designed wings (Figure S49–S54, Supporting Information).

To further justify the applicability of the dragonfly‐inspired cellular core of the wing designs, we have evaluated the performance of 3D‐printed samples under constant air pressure in the wind tunnel. As designed samples are 2D extruded and do not have airfoil, the out‐of‐plane distributed loading is the most effective measure for evaluating their performance as airplane wings (Figure 6b,c) of the results of the wind tunnel tests show that dragonfly‐inspired design without manufacturing limitation considerations can perform significantly better than the basic design, specifically for the samples with the higher height (up to 25% improvement in out‐of‐plane stiffness for *V*_2_ design in comparison to the basic design). In our study, *V*_2_ design demonstrates the highest out‐of‐plane stiffness under pressure. Since the lift force on airfoils is significantly higher than drag force (the lift‐to‐drag ratio is commonly in the range of 10–20 ^[^ ^68^ ^]^) and the smallest dimension of the wing is its height, out‐of‐plane stiffness is the most critical stiffness among the others. It is worth mentioning that the previously‐reported curved rib design with a higher thickness (h = 3.85mm) also outperforms the basic design in out‐of‐plane stiffness. In addition to the stiffness, the *V*_2_ design with lower height has higher natural frequencies, which implies less risk of resonance under dynamic loads or higher flapping frequencies for designing flying micro‐robots (Figure S54, Supporting Information).^[^ ^19^, ^69^, ^70^, ^71^ ^]^

The developed biomimicry approach presented in this study can design the cellular core of alternative lightweight wings to improve their structural performance. The airfoil shape that sandwiches the bio‐inspired cellular cores is assumed to be unchanged; since the wing's aerodynamic performance relies on the airfoil's geometry,^[^ ^72^, ^73^, ^74^, ^75^ ^]^ the dragonfly‐inspired reticulated core has a negligible effect on the aerodynamic performance. In summary, experimental results are presented in **Table** **1**.

**Table 1:** Table Comparison of the stiffness results of all cellular core samples with respect to the basic design (BD). Cases (1–3) represent experimental test results of the core wings under out‐of‐plane, torsional, and in‐plane point load, respectively. Cases (4,5) are identified as experimental testing of the core wings with 2.32 and 3.85mm heights under the out‐of‐plane a uniform air pressure distribution in the wind tunnel

Stiffness improvement (%) of cellular cores compared to the basic design (BD)										
		CD	V_0_	V_1_	V_2_	V_3_	V${}_{0}^{L}$	V${}_{1}^{L}$	V${}_{2}^{L}$	V${}_{3}^{L}$
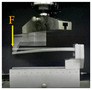	*Case (1)*	−2.5	5	1	2	1	−9.5	5.5	24.5	21.5
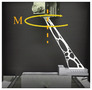	*Case (2)*	−5	−15	−12	1	6	−20	−12	5	15.5
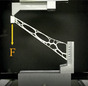	*Case (3)*	−14.5	−29	−31	−27	−26	−29	−26	−8	−18
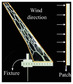	*Case (4)*	−18	−4	8	20	10	−10	1	6	9
	*Case (5)*	5	13	5	25	17	−2	13	21	9

The numerical and experimental results suggest that biomimicry of the dragonfly pattern for designing the core of airplane wing can have advantages in terms of mechanical performance. In this respect, dragonfly possesses long and relatively rigid wings that, through a high rate of flapping, it bears high pressure.^[^ ^45^, ^76^, ^77^, ^78^ ^]^ Through veins and stems, dragonfly wing form very stiff structures; mimicking such structure can be advantageous for long and cantilevered structures like commercial airplanes' wing. Besides biomimicry, the adopted design methodology based on graphic statics assume that all struts are under the same axial stress. Although this approach neglects bending moments in the struts, it provides a very well‐connected pattern with proper thicknesses that prevent weak hinged parts through the wing. In addition, the virtual load has some similarities with real load cases on airplane wings (Figure S7, Supporting Information). The adopted methodology also offer flexibility in terms of defining subdivision density for creating new cellular structures, a parameter that can be utilized to control the stiffness of cellular structures ^[^ ^58^ ^]^ As we know from experiments, the generated wings with smaller subdivision density usually perform better with higher stiffness, since the distribution of the structural weight tends to be concentrated more on the main structural members.

Last, we implemented our method as a web tool that can be accessed online (Figure S33, Supporting Information). Users can generate wing structures and export digital models through the web page.^[^ ^79^ ^]^ Besides, similar structures such as cantilever roofs and floors (Figure S40, Supporting Information) can also be generated using the same algorithm, which means our method has an impact on the design industry by providing a convenient machine learning tool for structural design.

### 2.7 Testing other Species with Convex Networks

**Figure 7:**
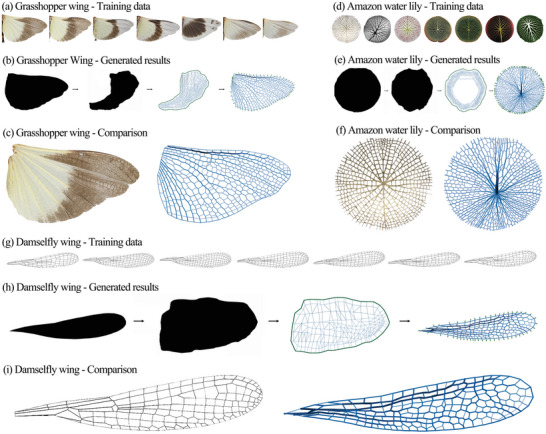
a) Training dataset of the grasshopper wing. b) Generated results of testing data of the grasshopper wing by machine learning. c) Comparison of the real grasshopper wing and the generated structural form. (The virtual loads are not shown.) d) Training dataset of the Amazon water lily. e) Generated results of testing data of the Amazon water lily by machine learning. f) Comparison of the real Amazon water lily and the generated structural form. (The virtual loads are not shown.) g) Training dataset of the damselfly wing. h) Generated results of testing data of the damselfly wing by machine learning. i) Comparison of the real damselfly wing and the generated structural form. (The virtual loads are not shown.)

Besides the training dataset for the dragonfly wings, we have also collected the training and testing data for other species to test whether our graphic statics and machine learning method works in generating patterns in other species as a generally applicable method. **Figure** **7** shows the details of the datasets and the results of the species (grasshopper wing, Amazon water lily, and damselfly wing). For each test, seven pieces of the image are collected, six of which become the training dataset while the rest one is used as the testing case.

The grasshopper wing is selected as the first experimental object since it is similar to the dragonfly wing but previous researchers fail to generate it using their generative methods for the dragonfly wing.^[^ ^8^ ^]^ To be specific, the dataset contains seven pieces of the grasshopper wings (Figure 7a). The GAN+ANN workflow (Figure 7b) is similar to the original workflow for the dragonfly wing. Figure 7c shows the result comparison of the real grasshopper wing and the generated structural form, with the analytical plots shown in Figure S23 (Supporting Information). The generated pattern has fewer members than the real pattern, especially in the domain with a small area and small circularity. But the overall distribution, especially for the main veins, is accurate. Our method performs as expected with a smaller training dataset in grasshopper wings.

Besides insect wings, some plants also have unique patterns in their rhizomes, for example, the Amazon water lily. The rhizomes of the amazon water lily serve as a supporting structure to hold its leaves, while the leaves provide buoyancy to make the amazon water lily float on the water. Therefore, the structure of the Amazon water lily might have similar structural properties as the dragonfly and the grasshopper wings, and it is worth exploring. To be specific, we have also collected a small dataset of seven pieces of amazon water lilies (Figure 7d). Figure 7e shows a similar workflow of the case of the Amazon water lily as the case of the grasshopper wing. Figure 7f and Figure S24 (Supporting Information) show the comparison of the result and the analytical plots. However, in the case of the Amazon water lily, the generated pattern has more members than the real pattern, especially in the domain with a small area and small circularity, and more veins were generated, accordingly. The overall distribution is less accurate than the case for the grasshopper wing, but still falls into our expectations.

Similarly, to finalize our experiments, we have lastly tested our method in damselfly wings (Figure 7g). The workflow (Figure 7h) and the result comparison (Figure 7i; Figure S25, Supporting Information) further prove the generality of our method. From the above observation, the conclusion can be reached that our graphic statics and machine learning method can be applied to generate structures by learning from natural species.

## 3 Conclusion

In this research, we showed that the geometric configuration of a 2D dragonfly wing skeleton can be used in the design of the cellular core of airplane wing structures using artificial intelligence. We investigated the use of reciprocal diagrams of graphic statics proposed by Maxwell combined with machine learning methods to analyze the graph network of the dragonfly wing and reconstruct similar networks for other boundary geometries; we present the potential application of the developed framework for designing lightweight and stiff wing structure in aerospace vehicles. Our research shows that the network of a dragonfly has a dual/reciprocal diagram that is unique and geometrically determinate. This property of the force diagram implies that the projected graph of the internal network of the wing can be treated as a determinate structural network and has a singular state of equilibrium. Surprisingly, if the members are sized according to the magnitude of this unique force diagram, their diameter matches the diameter of the members in the real wing. The results are significant since the force diagram of the wing is a new attribute that has not been well explored in generating a similar network for bio‐inspired structural systems. The correlation between the diameter of the real and generated wings sheds light on information about the primary and secondary members of the wing. The primary members are connected to vertices that are reciprocal to larger force triangles in the force diagram. These triangles can be identified and used as the force polygons of the primary members.

This new observation about the geometry of the force diagram and the magnitude of the internal forces in the geometry of the cellular core of wing allows us to design high‐performance wing structures for various boundary geometries using machine‐learning, which can successfully generate structural patterns for any chosen boundary geometry similar to the real dragonfly wing.

Our machine learning‐enabled generative method takes advantages of both image‐based and vector‐based neural networks. The image‐based neural network receives the input boundary image and generates the output force diagram. At the same time, the vector‐based neural network predicts the geometric information of the graph and generates the final structural form using reciprocal diagrams of graphic statics. The behavior of both generated and real wings under a uniform out of plane pressure depicts the same deformation and stress pattern. The difference in average stiffness between them is as low as 20 percent, which is remarkable since optimizing the mechanical properties was not the objective of the proposed generative approach.

We conducted mechanical experimentation on 3D printed dragonfly‐inspired samples as the cellular core of a wing for a scaled‐down Boeing 777 wing frame. Our experimental results showed that the developed methodology can lead to dragonfly‐inspired wing designs with up to 25% higher out‐of‐plane stiffness compared to the conventional wing designs. Combining the introduced method with other optimization methods can push further this envelope to achieve stiffer yet lighter solutions.

Our method can generate the entire structural pattern from an image of the wing boundary with no prior information about the location of the primary members. Designers can input a customized boundary and generate similar structural networks through the developed interactive platform. Moreover, our proposed generative approach provides additional information about the thickness of primary and secondary members by relating the design to the geometric equilibrium of forces in its reciprocal diagram. Our methodology is extendable to a wider range of species, including the grasshopper wing and the Amazon water lily that consists of convex polygons. Thus, our approach in design can be adapted to create structural forms, and their associated form diagrams, inspired by the other biological structures of insects and plants merely by inputting the boundary geometry. Here, we chose to investigate dragonfly wings since the data related to the wing is more abundant than the other species.

The ultimate objective of this research was to pave the road for further research in linking machine‐learning models with design principles derived from natural systems and applying such an approach in generating lightweight and efficient structures in various scales. Our proposed methodology, however, has limitations that need to be addressed in future steps. (i) Our current dataset of the dragonfly wings and other species is limited, and a more extensive dataset is required to be collected to increase the accuracy of the generated results. (ii) the current method is limited to 2D reciprocal diagrams; thus, the results are limited to 2D structural networks. Future research can consider 3D/polyhedral graphic statics in generating a 3D structure of the wing with folded plate geometry. (iii) The scope of this study was limited to the morphological properties of the dragonfly network and mainly generates similar configurations without considering the out‐of‐plane loading scenarios as a parameter in design generation at the beginning of the design process. (iv) The mechanical performance after each round of generation can also be fed back into the design process to relate the bending stiffness and the degrees of subdivision. (iv) The vein network of the wing was considered as the primary structure in our work, while the surface patches in the real wing restrain the kinematic degrees of freedom of the structure. The role of these patches and, thus, their contribution should be considered in the subsequent development steps.

## Conflict of Interest

The authors declare no conflict of interest.

## Author Contributions

M.A. devised the project and the main conceptual ideas and directed the project. H.Z. worked out the technical details for the generation of the structural forms using the dragonfly wing patterns. M.H. provided methodology for the mathematical methods of the project. A.H.A. directed and devised the project pertaining to the numerical simulation and physical experiments for bio‐inspired cellualr structual application. H.M. performed the numerical calculations and physical experiments on the generated wings. M.A. and A.H.A. supervised the findings of this work. All authors discussed the results and H.Z. and H.M. wrote the manuscript with support from M.H., A.H.A., and M.A.

## Supporting information

Supporting Information

Supplemental Video 1

## Data Availability

The data that support the findings of this study are available from the corresponding author upon reasonable request.
