# Supplementary material for: Dragonfly‐Inspired Wing Design Enabled by Machine Learning and Maxwell's Reciprocal Diagrams
Source: Adv Sci (Weinh). 2023 Apr 29;10(18):2207635. doi: 10.1002/advs.202207635 (PMC10288228; doi:10.1002/advs.202207635)
Supplement: Supplementary file 1 — Supporting Information [file ADVS-10-2207635-s002.pdf]

Dragonfly-Inspired Wing Design Enabled by Machine Learning and Maxwell's Reciprocal Diagrams

Hao Zheng, Hossein Mofatteh, Marton Hablicsek, Abdolhamid Akbarzadeh*, Masoud Akbarzadeh*

H. Zheng, M. Akbarzadeh
Polyhedral Structures Laboratory, Department of Architecture, Weitzman School of Design, University of Pennsylvania, Philadelphia, PA, 19146 USA.

H. Zheng
Department of Architecture and Civil Engineering, City University of Hong Kong, HKSAR, China.

M. Akbarzadeh
General Robotic, Automation, Sensing and Perception (GRASP) Lab, School of Engineering and Applied Science, University of Pennsylvania, 3330 Walnut St, PA, 19104 Philadelphia.
Email Address: masouda@design.upenn.edu

H. Mofatteh, A.H. Akbarzadeh
Department of Mechanical Engineering, McGill University, Montreal, QC, H3A 0C3 Canada.

A.H. Akbarzadeh
Advanced Multifunctional and Multiphysics Metamaterials Lab (AM^3L), Department of Bioresource Engineering, McGill University, Montreal, QC, H9X 3V9 Canada.
Email Address: hamid.akbarzadeh@mcgill.ca

M. Hablicsek
Mathematical Institute, Leiden University, Leiden, 2333CA The Netherlands.

A Supporting Information

A.1 The geometry of dragonfly wings

In the case of the dragonfly wing (Figure S1)[1], its 2D pattern can be regarded as a structural form whose edges have unique structural thicknesses. The morphological research shows its overall geometry contains multiple closed polygons as cells [2, 3, 4, 5]. Besides, its joint [6] can be basically categorized as three shapes: (Y), (T), and (+) shapes [7, 8]. In this research, we intend to construct Maxwell's reciprocal diagram for the geometry of the dragonfly wing and report the properties of these dual/reciprocal diagrams and draw conclusions by physical and numerical analyses.

A.2 Algebraic solution of Graphic Statics

Two-dimensional graphic statics was developed and practiced in the late nineteenth century [9, 10, 11, 12]. In 2D graphic statics, the geometry of the structure and its equilibrium are represented by two diagrams,

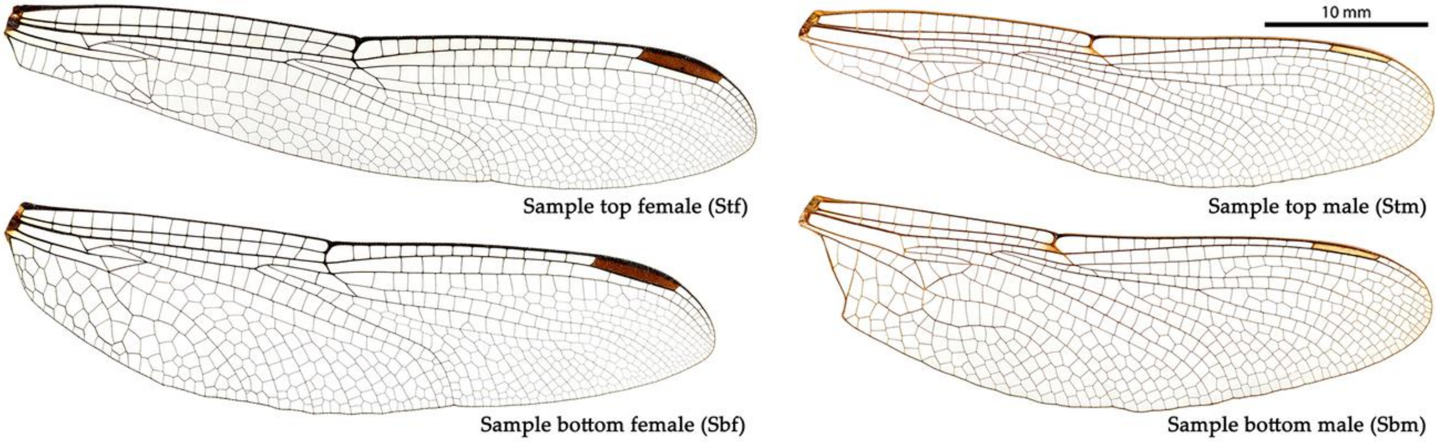

Figure S1: Dragonfly wing samples and their patterns.

.

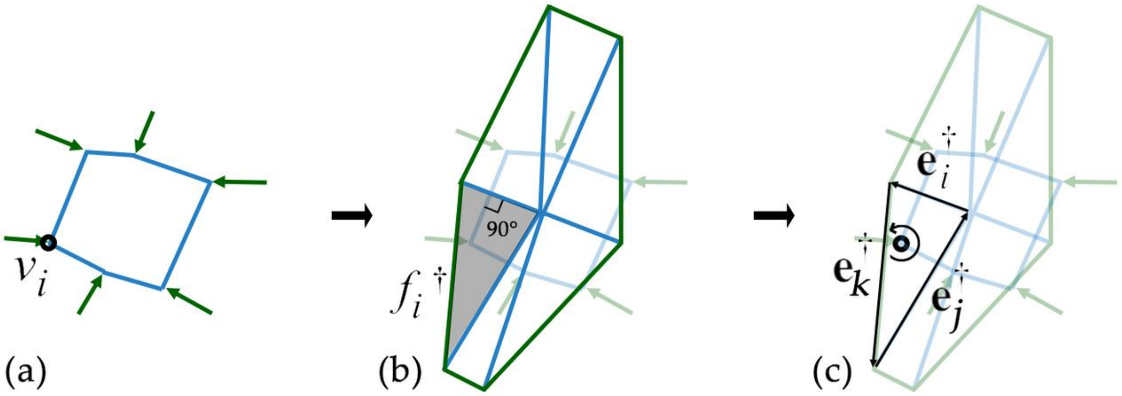

Figure S2: Algebraic solution: building equations.

called the *form* and the *force* diagrams. The form diagram includes the location of the supports and the applied loads, while the force diagram represents the equilibrium and the magnitude of the forces by closed polygons. These two diagrams are *reciprocal*: vertices, edges, and faces of one diagram correspond to faces, edges, and vertices of the other, furthermore, corresponding edges of the two diagrams are perpendicular [13]. The elements of the dual diagram are usually denoted with the superscript †.

This reciprocal relationship is illustrated by Figure S2. In fact, Figure S2a shows a form diagram, which contains six internal edges and six external forces (edges). In Figure S2b, the proposed edges are presented as the dark blue lines, which are perpendicular to the edges in Figure S2.

An algebraic framework was presented in [14] to generate a dual diagram from the primal diagram. In fact, for each polygon ($f_i^\dagger$), a group of equations can be derived based on its edges ($e_i^\dagger$) (Figure S2c). Indeed, since the face ${f_i}^\dagger$ is a closed polygon, the sum of its edge vectors ${\mathbf{e}_j}^\dagger$ should be zero. Hence, we obtain a vector equation

$$\sum_{e_j^\dagger} {\mathbf{u}_j}^\dagger q_j = \mathbf{0} \tag{1}$$

where the sum runs over the edges $e_j^\dagger$ of the face $f_i^\dagger$, $\mathbf{u}_j^\dagger$ denotes the unit edge vector of the edge $e_j^\dagger$, and the q_j are the variables representing the lengths of the edges $e_j^\dagger$. Writing these equations around every face $f_i^\dagger$ provides a linear equation system for the edge length vector $\mathbf{q}$ which can be described by a $[2v \times e]$ matrix, called the equilibrium matrix $\mathbf{A}$:

$$\mathbf{A}\mathbf{q} = \mathbf{0}. \tag{2}$$

If the equilibrium matrix, $\mathbf{A}$ is of full rank, then the only solution to Eq. 2 is the zero vector $\mathbf{q} = \mathbf{0}$. In

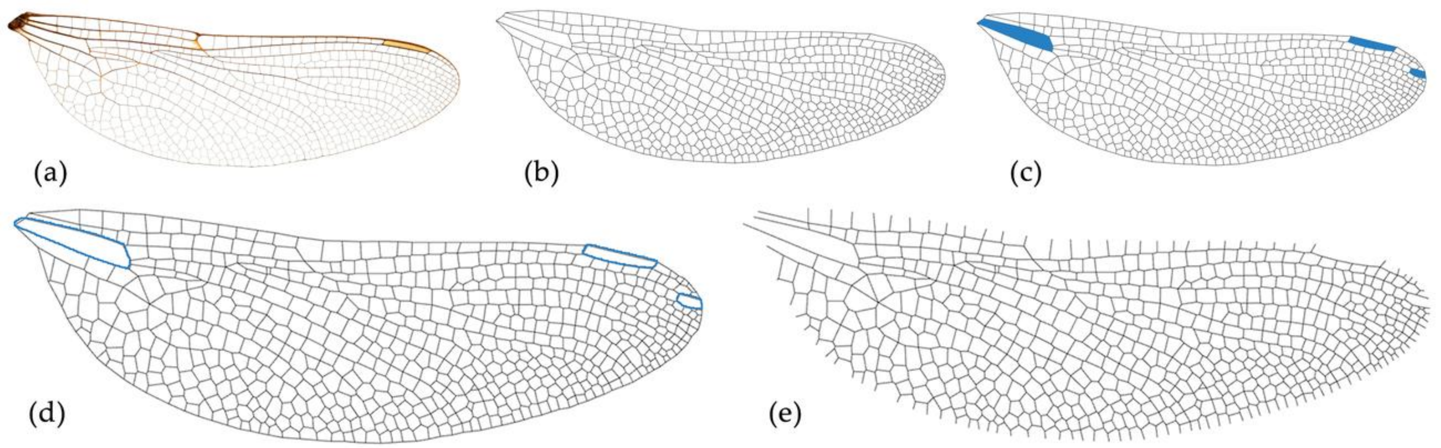

Figure S3: Data preparation: (a) original dragonfly wing image; (b) vectorized form; (c) modifying non-convex cells (3 out of 573 (0.52%) cells); (d) modified convex form; and (e) internal form.

this case, the force diagram collapses to a point. If the equilibrium matrix, $\mathbf{A}$ is not of full rank, then there are infinitely many solutions to Eq. 2.

A.3 Dimension of The Solution Space and GDoF of The Dual

The dimension of the solutions $\mathbf{q}$ satisfying Eq. 2 is equal to the dimension of the right nullspace of the equilibrium matrix, $\mathbf{A}$. I.e. if we have r independent equations, the dimension of the right nullspace is equal to $e - r$. The r is the number of independent equations of the equation system 2 which is equal to the rank of the equilibrium matrix, $\mathbf{A}$.

This provides a definition of the Geometric Degrees of Freedom ([15]) of the dual diagram that measures the dimension of dual networks with parallel edges but different edge lengths. In fact, the dimension of the right null space of the equilibrium matrix, $\mathbf{A}$, equals the GDoF of the dual diagram.

For example, for a single node with e applied loads as a primal diagram, the equilibrium matrix is of shape $[2 \times e]$. As a result, the dimension of the solution space is $e - 2$, because $r = 2$, and the two linear equilibrium equations are independent. The dual diagram is a single polygon with $e^{\dagger}$ number of edges, hence, its GDoF is equal to $e^{\dagger} - 2$. In the case of $e = 3$, i.e, in the case when the dual diagram is a single triangle, the GDoF is one, meaning that the only way to alter the dual diagram without breaking the duality is scaling.

A.4 Constructing a Dual/Reciprocal Diagram for the Dragonfly Wing

The projected geometry of the internal members of the dragonfly wing on the 2D plane is transformed into a polyhedral network consisting of convex cells that can be studied with graphic statics.

Since we do not have a clear understanding of the external force of the dragonfly wing, the first step is to exclude the boundary edges of the dragonfly wing and consider the internal network of the edges. Figure S3 shows the process of the data preparation. The original dragonfly wing image (Figure S3a) is transformed into the vector-based geometry with convex and non-convex polygons (Figure S3b) using image processing techniques of edge detection in OpenCV. The non-convex polygons (Figure S3c) are slightly adjusted to make convex polygons (Figure S3d) by an algorithm that iteratively updates the coordinates of the vertexes. By removing the boundary edges, the internal form (Figure S3e) is generated.

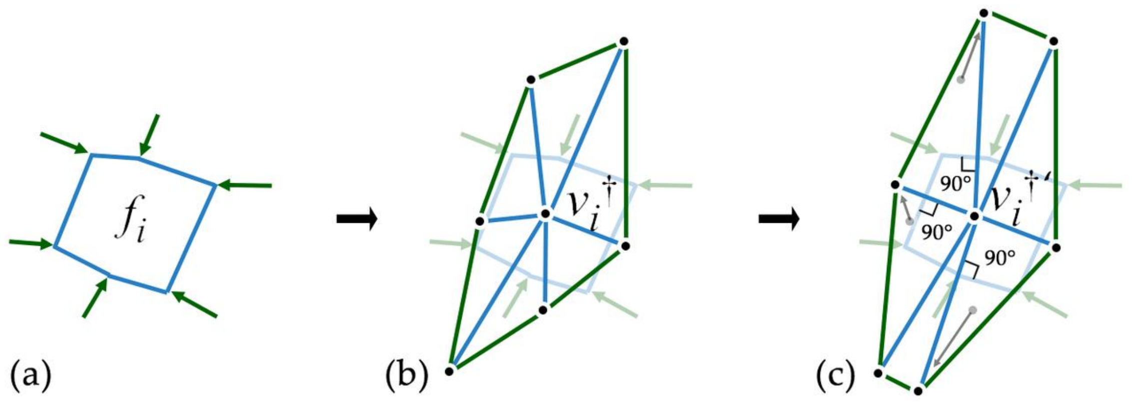

Figure S4: Geometric solution: iterative method to update the positions of vertices.

The simplified version of the dragonfly wing contains only geometric information, it is a representative form diagram of the dragonfly wing. Using the methods of graphic statics, its corresponding force diagram can be generated (Figure 1A in the main manuscript).

Finally, by mapping the force magnitude to the structural thickness, the corresponding structural form can be re-generated. The first goal of this research is to compare our generated dragonfly wings with the real dragonfly wings, and show whether the force distribution in the real dragonfly wing follows the rules of reciprocal diagrams. Once proven, the force diagram can explain the intrinsic logic of the design of the dragonfly wing by nature.

In the case of the internal network of a dragonfly wing, the dual diagram mainly consists of triangles, forcing the GDoF to be one. This means that the dual diagram is unique up to scaling.

A.5 Iterative solution

We used an iterative method as provided in PolyFrame software [16] that can generate the geometries of the force diagram with a deviation (tolerance). The algorithm starts by constructing the graph of the force diagram with incorrect edge lengths. Each polygon (f_i) in the form (Figure S4a) is transformed into a vertex ($v_i^{\dagger}$) in the force (Figure S4b). Then the iterative method is applied to optimize the position of each vertex ($v_i^{\dagger'}$) to make the corresponding edges perpendicular (Figure S4c). The difference between the vertical angle (90°) and the angle of the two corresponding edges (α) is defined as the deviation (δ). The optimization process works by a loop to minimize the value of δ (Figure 1b in the main manuscript). With this geometric method, an approximate solution of the force diagram can be generated.

To prove the consistency of the form and force in the dragonfly wing, experiments were executed taking the dataset of the dragonfly wings as examples. First, using the methods of graphic statics, the force diagram can be generated from the form diagram of the dragonfly wing (form-to-force) (Figure S5). Then, the form diagram can be re-generated from the generated force diagram using the same method (force-to-form) (Figure S6).

Figure S7 shows the distribution of the deviation of the form-to-force and the force-to-form generation. The maximum deviations for the form-to-force and the force-to-form processes are 46.64° and 20.78°, while the average deviations for the two processes are 2.64° and 0.64°. Most of the members have a deviation smaller than 1°, and there are decreasing numbers of members as the deviation increases. Only a few members have a deviation larger than 20° in the form-to-force process and 10° in the force-to-form process. Therefore, most of the members in the force diagram are accurately generated according to the corresponding form diagram, with the meaning of the internal force magnitude.

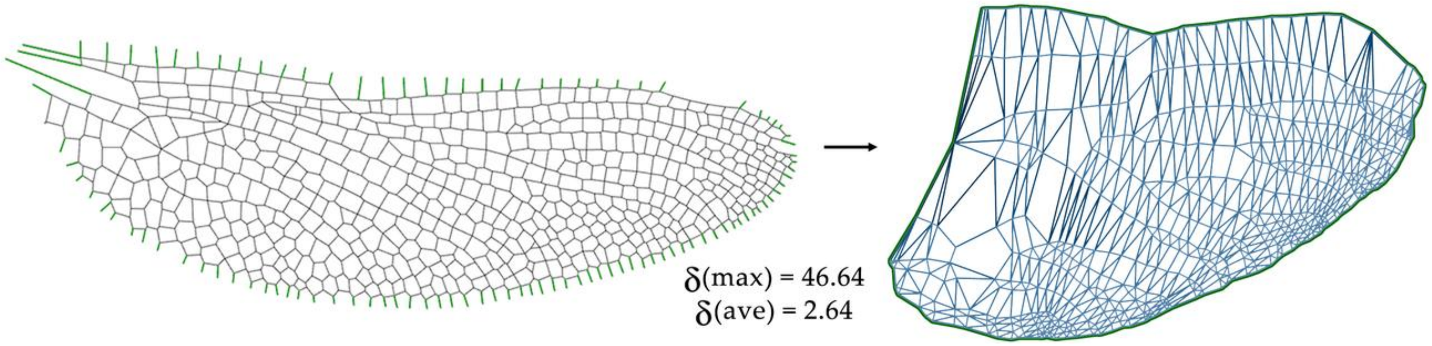

Figure S5: The maximum and average form-to-force deviations in degrees (δ) in the generation of force from the form. (The virtual loads are highlighted as green.)

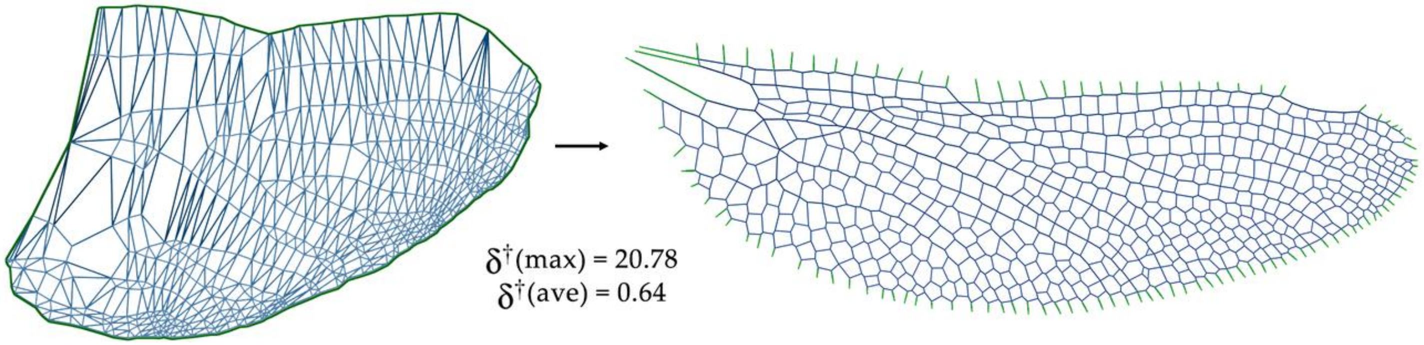

Figure S6: The maximum and average force-to-form deviations in degrees ($\delta^{\dagger}$) in the generation of form from the force. (The virtual loads are highlighted as green.)

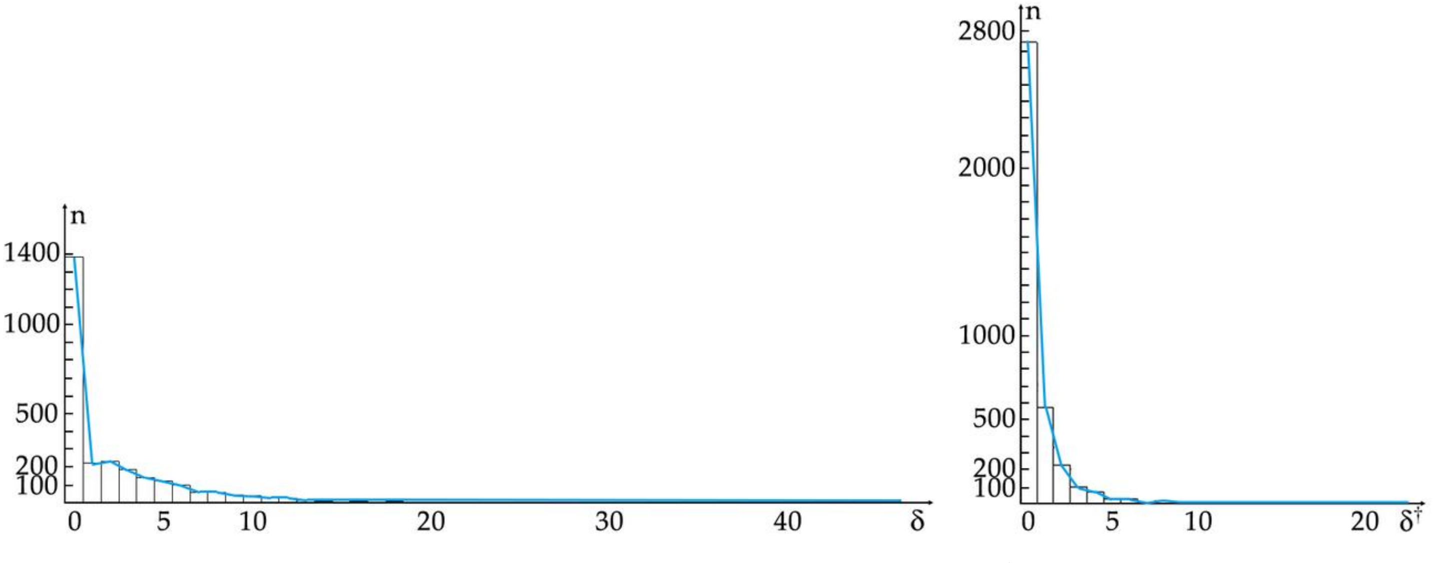

Figure S7: The distribution of two deviations. δ: form-to-force deviation in degrees. $\delta^{\dagger}$: force-to-form deviation in degrees. n: number of members.

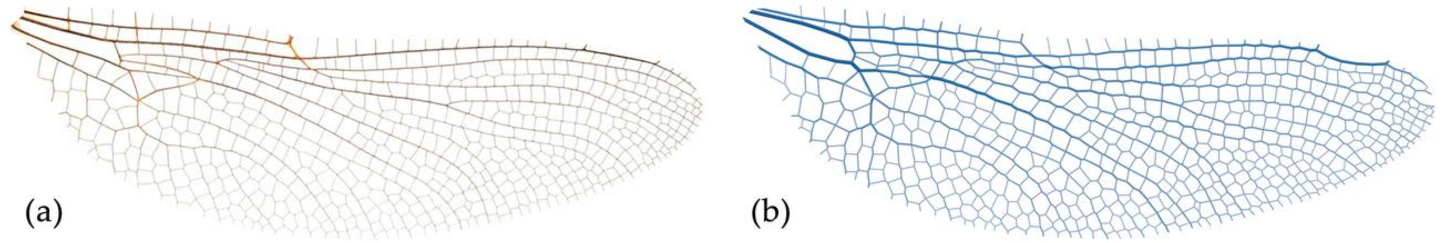

Figure S8: Internal structure: (a) real; and (b) generated. (The virtual loads are not shown.)

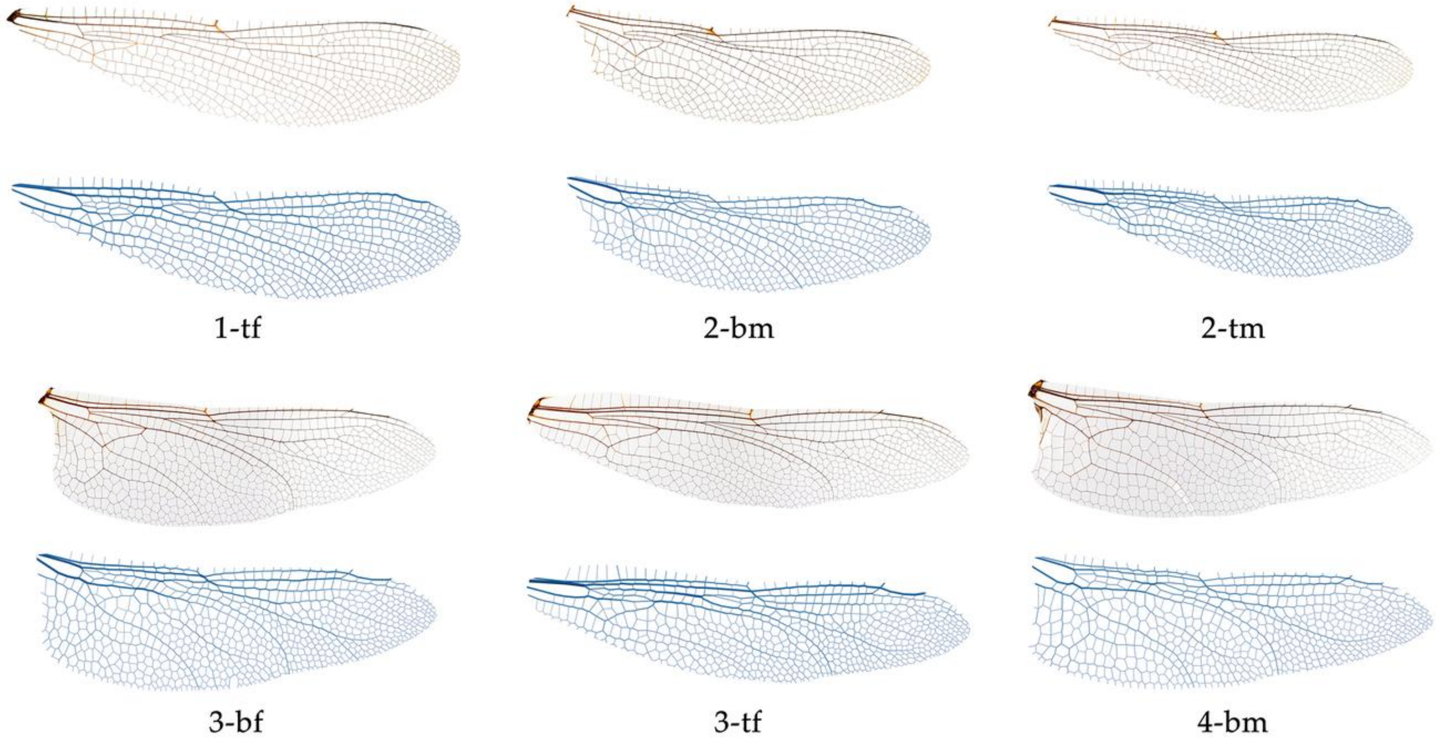

Figure S9: Comparison of the original and the generated structural thickness (internal only). (The virtual loads are not shown.)

A.6 Internal structural members

With the force diagram, the internal force for each form member can be obtained, thus the structural thickness can be inferred, which is proportional to the force magnitude. We first measure the thickness of the real dragonfly wing in pixels, the minimum and maximum widths are approximately 6 pixels and 60 pixels. We found that the ratio of the minimum and maximum force magnitude is also approximately 1:10, thus we remap the force magnitude into the range of 3 to 30, and generate the structural pipes with the radius of the remapped values. Therefore, the predicted internal structure is generated. Figure S8 shows the comparison of the real internal structure and the generated internal wing. Figure S9 shows the generated and the real internal wings in our testing dataset, and Table S1 shows their statistic values of the deviation and the accuracy.

In order to compare the similarity of the real and the generated internal wing, the accuracy measure (θ) is defined as the following process (Figure 1A). First, the radius (half of the structural thickness) of each structural member is extracted as r_i, and the normalized radius R_i is calculated (Equation 3), thus the value range of R_i is 0 to 1, which also strengthens the contribution of large members in measuring accuracy. Second, for each member in the real and the generated dragonfly wing, the accuracy θ_i is calculated based on the absolute error of these two values (Equation 4). Last, the overall accuracy θ is the weighted average

Table S1: Deviation and accuracy table for Figure S9: δ: form-to-force deviation in degrees; $\delta^{\dagger}$: force-to-form deviation in degrees; and θ: accuracy.

Sample	δ(max)	δ(ave)	% (<5º)	$\delta^{\dagger}$(max)	$\delta^{\dagger}$(ave)	% (<5º)	θ
1-tf	52.26	2.37	0.83013	21.54	0.57	0.98245	0.922349
2-bm	44.76	2.72	0.78754	17.44	0.69	0.97179	0.922321
2-tm	42.04	2.57	0.82237	42.68	0.69	0.97446	0.935846
3-bf	55.61	2.81	0.78835	38.69	0.68	0.98546	0.891284
3-tf	35.35	3.13	0.76083	30.45	0.68	0.98846	0.923929
4-bm	38.43	3.01	0.76544	29.68	0.70	0.98057	0.917173
Average	44.74	2.77	0.78833	30.08	0.67	0.97624	**0.918817**

of all θ_i based on the volume V_i of each member (Equation 5).

$$R_i = \frac{r_i - min(r)}{max(r) - min(r)} \tag{3}$$

$$\theta_i = 1 - |R_i^{real} - R_i^{generated}| \tag{4}$$

$$\theta = \frac{\sum_{i=1}^{N} \theta_i \cdot V_i}{\sum_{i=1}^{N} V_i} \tag{5}$$

According to the statistics, in the force-to-form generation, all the six testing wings have an average deviation of 2.77°, and 78.83% of the members have a deviation smaller than 5°. In the form-to-force generation, the average deviation is 0.97°, and 97.62% of the members have a deviation smaller than 5°. The overall accuracy is 91.9%, which shows a high similarity between the generated and the real internal dragonfly wing.

A.7 External structural members

With the above experiments on the internal force and form, we further investigate the external force and form of the dragonfly wing. The purpose of adding the external forces is to represent the structural thickness of the boundary members, thus we regard the external force pattern as a set of compression forces acting from the boundary. Although the actual external force pattern might be different and complicated, this modification can construct the entire force diagram as a compression-only force pattern, thus the structural thickness of the entire wing can be predicted.

Since the external force cannot be extracted directly from the form diagram, we measure the thickness of the external edges in the structural form of the wing, and map the pixel counts to the lengths of the corresponding edges in the force diagram (Figure S10). Each edge in the force should be perpendicular to the boundary of the form, thus both the lengths and the directions can be inferred from the original form image. With this method, the external force diagram can be generated with correct length values.

With the external force diagram (Figure 1h in the main manuscript), the entire force diagram (Figure 1j in the main manuscript) can be obtained by merging the internal (Figure 1f in the main manuscript) and external force diagram. By transforming the entire force diagram back to the structural form, the structural thickness of the entire wing can be inferred, thus the predicted dragonfly wing is complete with both internal and external members (Figure 1k in the main manuscript).

Figure S11 shows one example of the comparison of the real dragonfly wing and our generated dragonfly wing. Using our graphic statics method, the structural thickness is accurately predicted with an accuracy of 92.5%. Figure S12 shows the plot of the normalized radius (R_i) of the real and the generated dragonfly wings. The distributions of the thickness are highly similar, which further shows a high accuracy of the prediction.

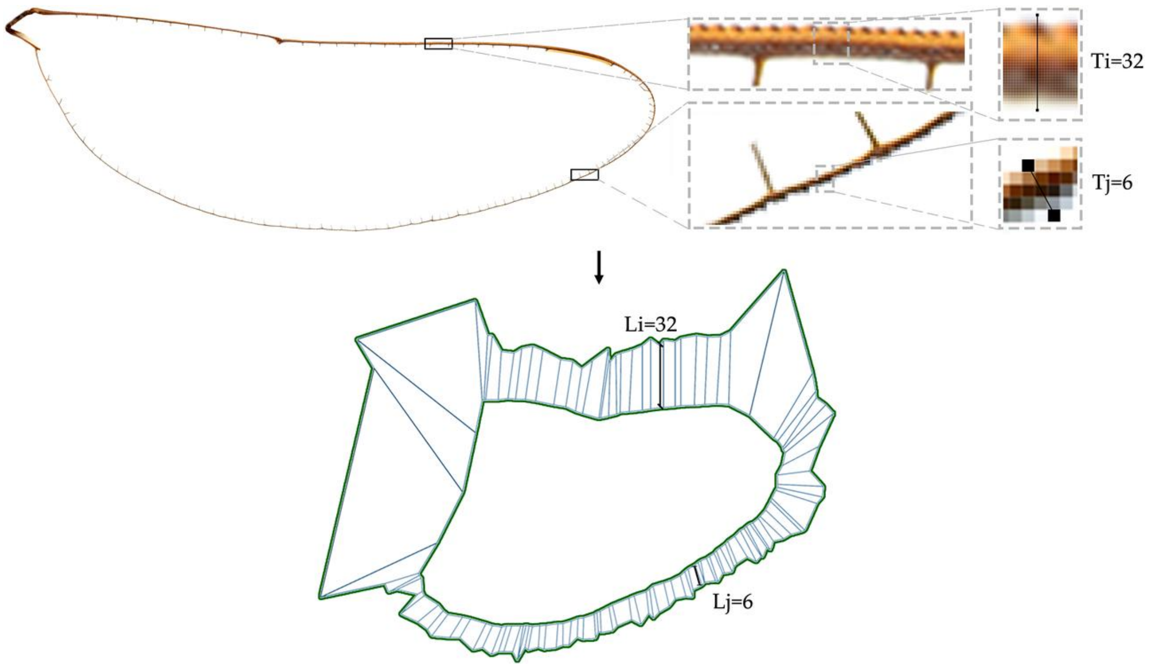

Figure S10: Boundary thickness measurement based on the center perpendicular pixel coordinates, and the mapping to the external force diagram. T: the thickness in pixels; and L: the length in units.

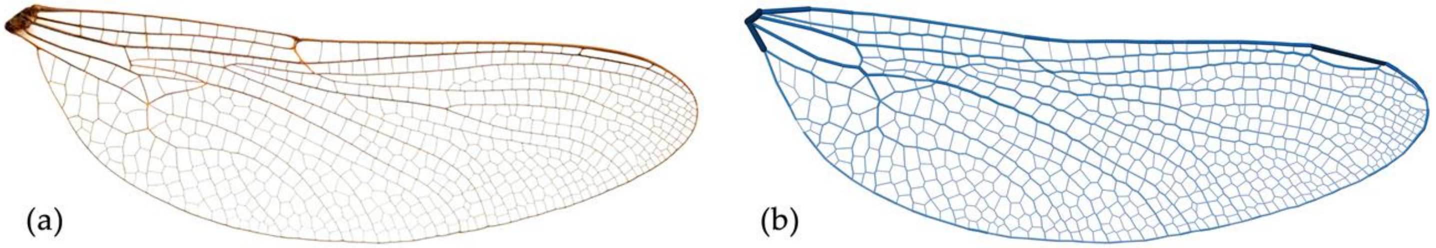

Figure S11: Forms with structural thickness (a) real; and (b) generated. (The virtual loads are not shown.)

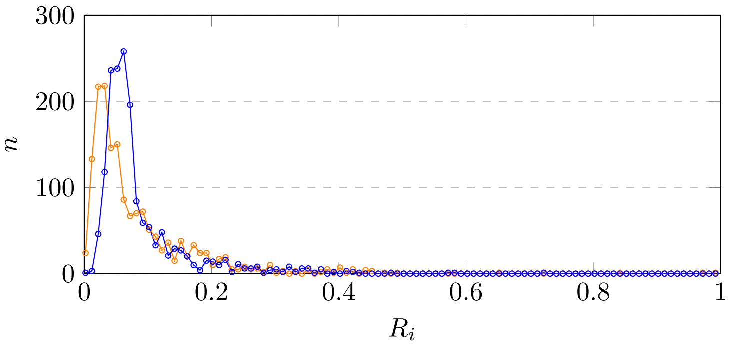

Figure S12: The plot of the distribution (number of members n) of the normalized radius (R_i) for the real dragonfly wing (orange) and the generated dragonfly wing (blue).

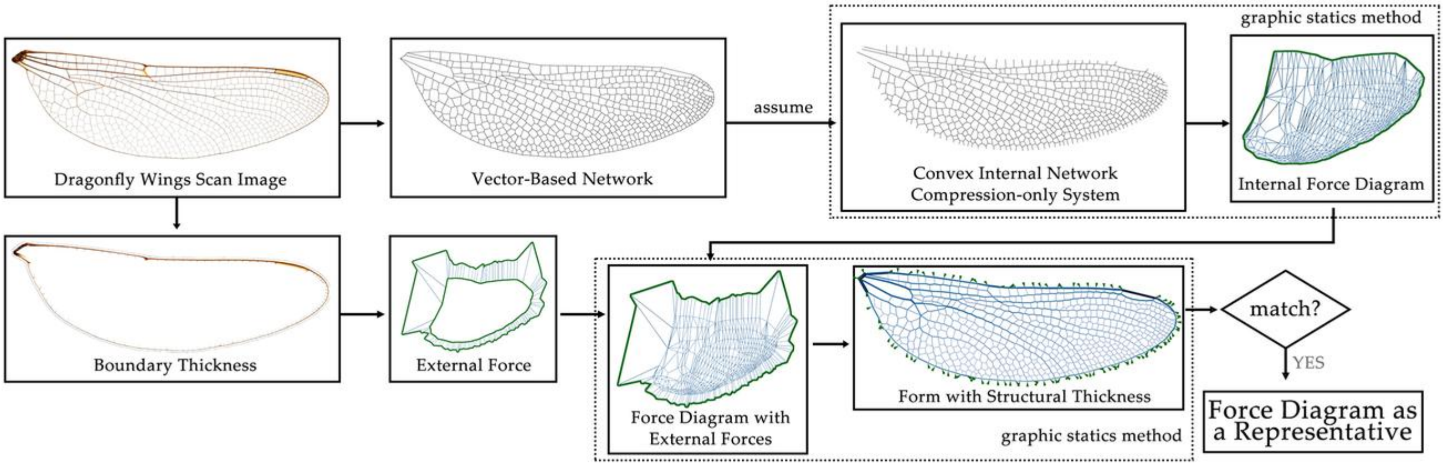

Figure S13: Flow chart of applying graphic statics in generating the form and force of the dragonfly wing.

Lastly, we conclude the workflow as Figure S13 shows. First, the structural thickness is partially removed from the original dragonfly wing image, with only the geometry of the wing left. Then, our graphic statics method transforms the geometry of the form into the geometry of the force. By mapping the edge lengths in the force geometry to the structural thickness of the edge in the form geometry, the wing is generated again with the predicted structural thickness. The accuracy of the comparison of the real and the generated dragonfly wings is high, which shows the evidence that: 1) graphic statics method can be used to analyze the dragonfly wing structures; 2) the intrinsic logic of the structural property of the dragonfly wing can be represented as the force diagram.

A.8 Results of the graphic statics

Using our graphic statics method, we proceeded with another 24 pieces of dragonfly wings collected from John Tann [1] to find evidence of whether the accuracy is still high for other cases. The comparison of the generated and the real dragonfly wings are shown in Figure S14 with the statistics in Table S2.

Considering the entire wings with both the internal and external members, it shows similar results compared with the internal-only testing. The form-to-force generations for all 24 cases have an average deviation smaller than 3.3°, and the overall average deviation is 2.79°. In the force-to-form generation, the average deviation for each case is smaller than 1°, and the overall deviation is 0.73°. And all cases show an accuracy higher than 88.1%, and the overall average accuracy is 92.0%. Therefore, through the comparison of more pieces of dragonfly wings, the high accuracy of the comparison of the real and the generated dragonfly wings shows the evidence that: 1) the methods of graphic statics can be used to analyze the dragonfly wing structures; 2) the intrinsic logic of the structural characteristics of the dragonfly wing can be represented as the force diagram.

From the experiments of the graphic statics, the conclusion can be reached that the force diagram with a convex pattern is a representative of the structural form of the dragonfly wing, which reveals the design logic of the internal force patterns. Therefore in this section, the force diagram is used as the main training material to learn and apply the design logic of the dragonfly wing from nature.

A.9 Main path extraction

Pre-processing needs to be applied to the dataset to strengthen the learnable features. Previous research identifies the main veins of insect wings by identifying the horizontally continuous members as the main veins, from which the secondary wing patterns are tiled to each area separated by the main veins [17].

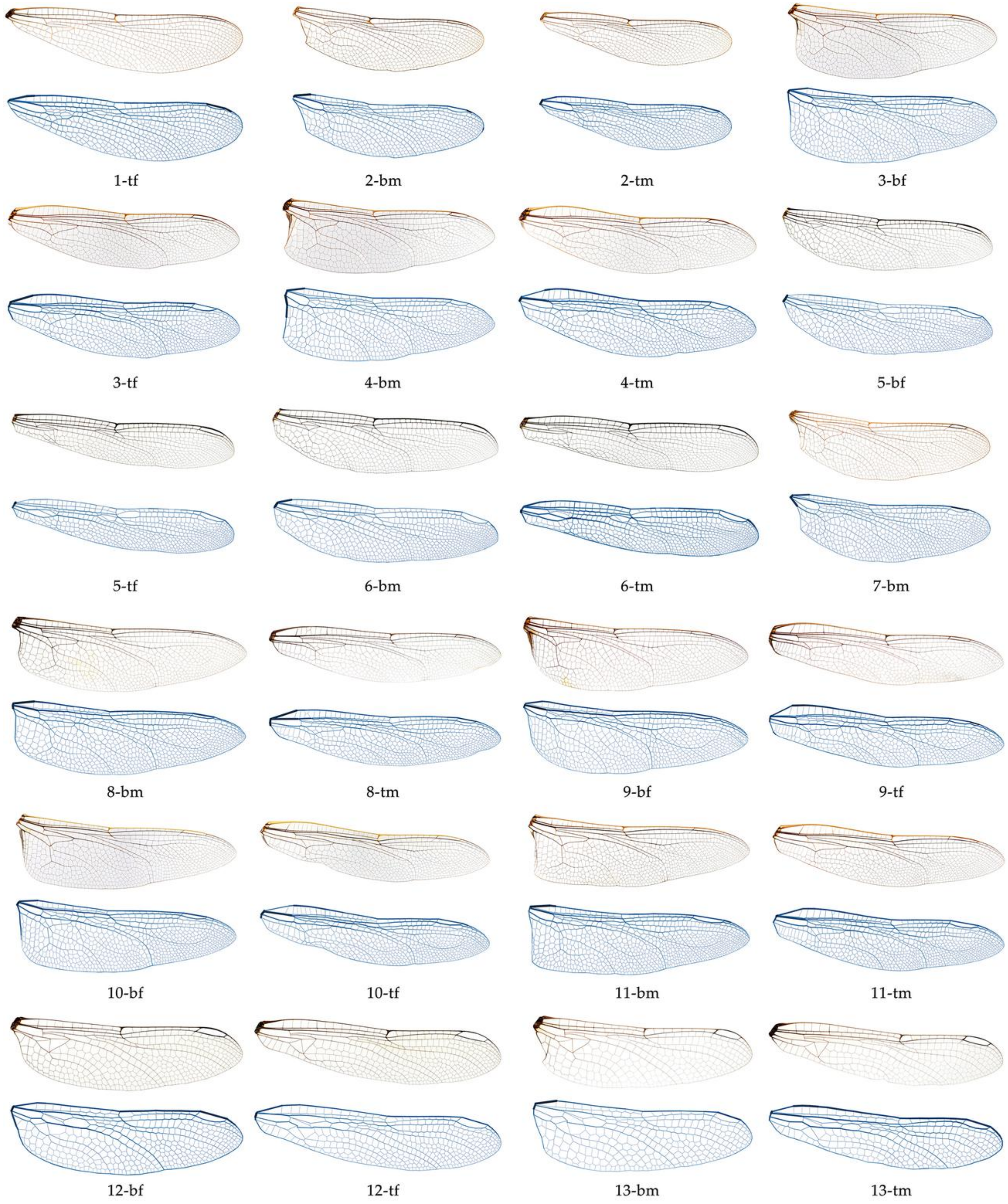

Figure S14: Comparison of the original and the generated structural thickness. (The virtual loads are not shown.)

Table S2: Deviation and accuracy table for Figure S14. δ: form-to-force deviation in degrees. $\delta^{\dagger}$: force-to-form deviation in degrees. θ: accuracy.

Sample	δ(max)	δ(ave)	% (<5º)	$\delta^{\dagger}$(max)	$\delta^{\dagger}$(ave)	% (<5º)	θ
1-tf	52.26	2.37	0.830133	23.11	0.59	0.981198	0.928593
2-bm	44.76	2.72	0.78754	16.89	0.68	0.971799	0.935975
2-tm	42.04	2.57	0.822371	43.52	0.69	0.974462	0.93833
3-bf	55.61	2.81	0.78835	39.83	0.71	0.976597	0.901853
3-tf	35.35	3.13	0.760836	32.45	0.74	0.973422	0.932262
4-bm	38.43	3.01	0.76544	31.96	0.75	0.96969	0.918975
4-tm	55.01	3.08	0.769002	25.7	0.69	0.975466	0.933478
5-bf	56.61	2.8	0.790818	22.98	0.71	0.971765	0.934398
5-tf	58.88	2.8	0.798701	29.23	0.73	0.968781	0.929365
6-bm	85.4	2.7	0.795924	38.76	0.69	0.976645	0.940173
6-tm	41.53	3.24	0.781451	40.95	0.87	0.973387	0.911666
7-bm	48.28	2.83	0.786388	26.26	0.76	0.96688	0.927764
8-bm	27.71	2.45	0.812821	30.98	0.58	0.980793	0.907822
8-tm	42.67	2.65	0.811239	23.43	0.62	0.980288	0.930594
9-bf	45.76	2.91	0.775819	36.79	0.78	0.970803	0.881393
9-tf	51.55	3.16	0.765987	59.71	0.91	0.963	0.901833
10-bf	50.87	2.63	0.799039	25.26	0.72	0.974135	0.915933
10-tf	40.58	2.9	0.777516	54.73	0.73	0.969521	0.89709
11-bm	56.17	2.89	0.779755	29.63	0.73	0.972338	0.9047
11-tm	47.78	2.82	0.783315	28.59	0.71	0.972112	0.934333
12-bf	58.43	2.64	0.817714	59.46	0.79	0.970899	0.905025
12-tf	46.87	2.66	0.823344	84.98	0.82	0.965004	0.944747
13-bm	52.52	2.52	0.821301	95.77	0.89	0.964482	0.907044
13-tm	45.36	2.71	0.816527	26.88	0.71	0.974257	0.911623
Average	49.18	2.79	0.7942	38.66	0.73	0.9724	**0.91979**

Inspired by the workflow of identifying the main veins, we develop a similar method to extract the main path of a force diagram of the dragonfly wing (Figure S15). The reason for this process is to simplify the force diagram and generate an intermediate geometry showing a stage between the boundary of the force and the entire force diagram. Another representation of the force main path is to combine the middle points in the force geometry as pixel values in the main path image rather than the color-coding (Figure S16).

In the first representation of the main path, the external force geometry is directly identified as the main path, while the internal force geometry is proceeded as the following workflow: 1) starting from the left root point, as clockwise, the script traverses all horizontally continuous lines from each point on the boundary of the internal force geometry until it reaches a point that is previously reached or it reaches a point on the boundary; 2) all the lines that are traversed together split the internal force boundary into several areas; 3) for each area, it contains polygons from the entire force diagram, the average area of all the polygons in the area is mapped as values in the R channel of an image, the number of lines in the area is mapped as values in the G channel of the same image, while the main paths are marked in the B channel of that image; 4) the force diagram with main paths is then generated as a color-coded image (Figure S15b) showing a simplified version of the entire force diagram image (Figure S15a). With this main path extraction process, we generate an intermediate image between the force boundary image and the entire force diagram image, which helps increase the efficiency and accuracy in the following machine learning process.

In the second representation of the main path (Figure S16), the R channel with a value of either 0 or 255 represents the existence of regions of each main path, the B channel with a value of either 0 or 255 represents the existence of the middle point of each edge in each region, and the G channel represents the length of the corresponding edge in the force geometry if the value in the B channel shows an existing

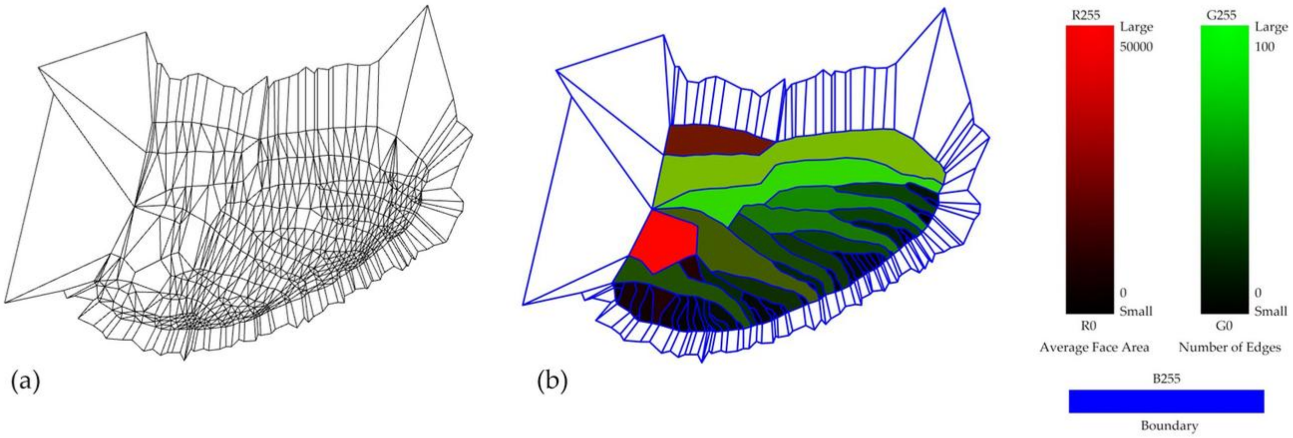

Figure S15: Extracting main path of the entire force: (a) original entire force diagram; and (b) force diagram with main paths.

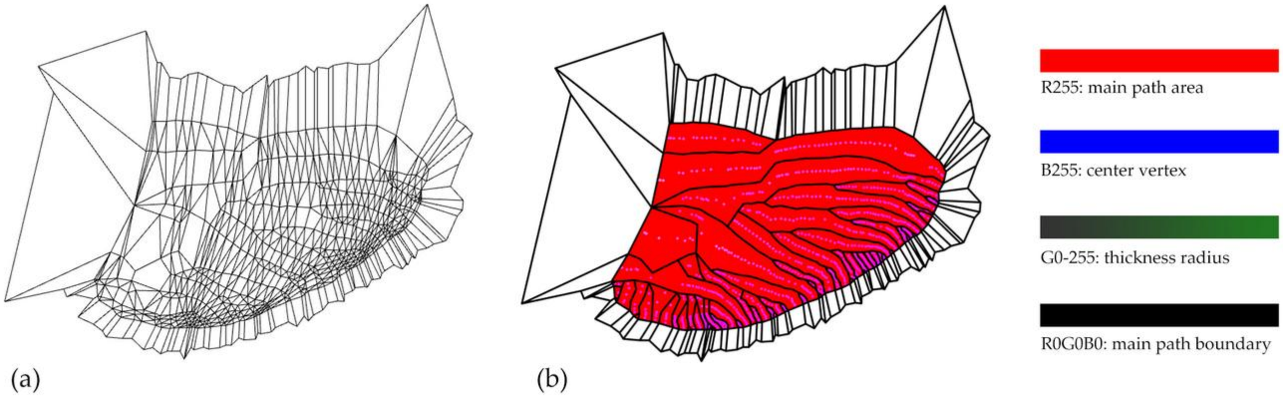

Figure S16: Main path combining the vertexes information: (a) original entire force diagram; and (b) force diagram with main paths and pixel values.

middle point in that pixel. If the values in the R, G, and B channels are all 0, the pixel is regarded as the Black channel, which represents the boundaries of the main path. This force main path can store both the pixel-based information of the boundaries of the main path, and the vector-based geometric information of the force lines.

A.10 Image-based machine learning

In the next step, we apply machine learning techniques to learn the mapping between each stage of the dragonfly wing data, and generate the force diagram from the form boundary. From the perspective of designers, the goal is to generate a structural form, which follows the same logic as the dragonfly wing, from the user-defined boundary of the form.

Figure 3 in the main manuscript shows the method we develop. Initially, we developed two methods. For the first method, the entire process starts from training three machine learning models, in which the first model learns the mapping between the form boundary and the force boundary, the second model learns the mapping between the force boundary and the force main path, and the third model learns the mapping between the force main path and the force diagram. However, in the first method, the geometries of a force diagram need to be reconstructed from the image manually to maintain the precision. Thus, the force geometry can be directly generated and proceeded into the structural form. The first method produces more visible information for a human to understand, while the second method generates abstract information for the machine to rebuild the geometry. Since the force diagram is representative of the structural form and it contains enough geometric information, the process in this step ends with the generation of the force diagram, and the following process regarding the generation of the structural form from the force diagram is introduced in the following sections.

Technically, the data format for the machine learning process is image-based, thus the image-to-image machine learning technique, Generative Adversarial Network (GAN) [18, 19], is applied in the learning and generating tasks. The reason why we use GAN is that, the data in each stage is represented as images. First, the form boundary and the force boundary are regions covering areas in a 2D plane, thus 2D images can best reflect the features of the boundaries. Then in the force main path and the force diagram, colors are used to represent the features of the interior geometries of the force, thus color-coded images can also best reflect the features. Meanwhile, the workflow is to generate those images one by the other with a clear order of an input image and an output image, therefore, image-to-image machine learning models, especially conditional GANs, are expected to work properly. Besides, compared with other image-based models such as diffusion models [20], GAN models are easier to train and perform better in a small dataset. While diffusion models require an image and a prompt input to generate output images, GAN models only require inputting an image, which better matches the workflow of our task.

In the neural network structure of the Generator in GAN, an input image is proceeded into an output image with the same size using convolutional, residual, and deconvolutional layers. Another neural network, the Discriminator, works to distinguish the image generated by the Generator from the ground truth image. The Generator feeds forward the generated result to the Discriminator, while the Discriminator feeds back the loss and gradient to the Generator. Thus the Generator is trained to generate the fake images closer to the ground truth, while the Discriminator is trained to tell the fake image better apart. The two networks are "competing" with each other, thus this system is called "adversarial".

Figure S17 shows the dataset used in the training process. We have proceeded with the dataset of 25 pieces of dragonfly wings, and there are five images for each piece (form boundary, force boundary, force main path version 1, force main path version 2, and force diagram). We use the first 21 pieces as the training set and the left 4 pieces as the testing set. To increase the size of the dataset, we apply image augmentation techniques to rotate the training images with -15, -10, -5, 0, 5, 10, 15 degrees.

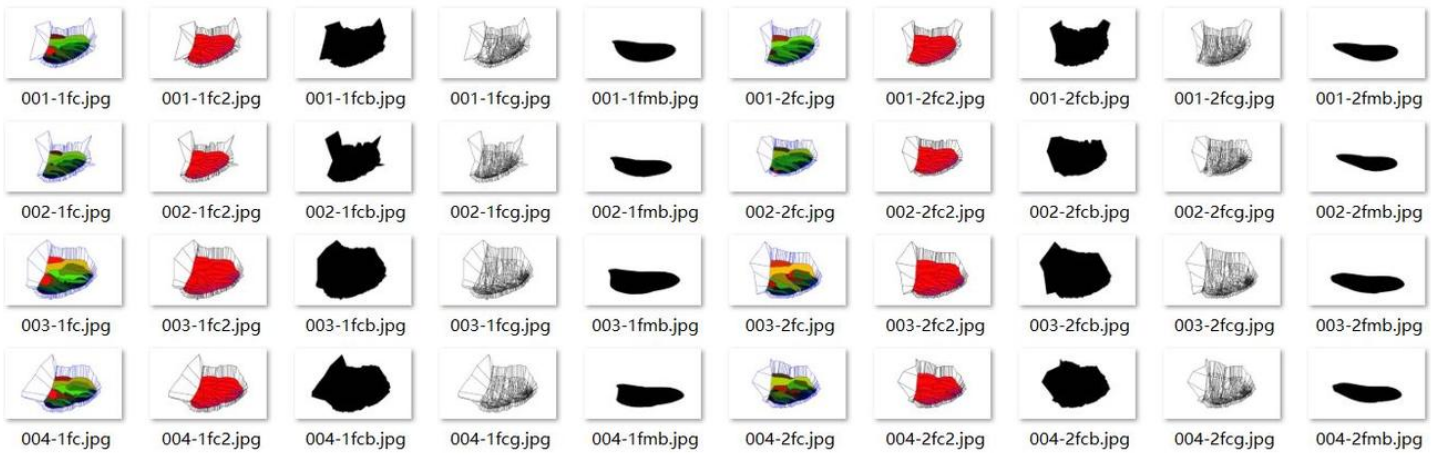

Figure S17: Examples of the machine learning model 1, 2, 3, and 5 dataset (25 pieces * 5 images * 7 rotating copies, 1000*600 pixels) (21 pieces as training set, 4 pieces as testing set).

In the training process, we use the same set of hyperparameters for all three models. The width of the images is 1000 pixels, and the height of the images is 600 pixels. The learning rate is constantly 0.0002 for the first 140 training epochs, and it is decreasing to 0 for the rest 60 training epochs. The training time cost for each model is 7.49 hours for a Tesla V100 computing GPU. Figure S18 shows the loss of the Generator (G) and the Discriminator (D) during the training process of the three models. The changing of the peaks and valleys indicates that G and D are competing with each other, and updating the parameters. This phenomenon is a signal showing that the training was successful.

Besides, for the machine learning method 2, a post-processing is applied to the generated image to reconstruct the force geometry (Figure S19). To be specific, the R channel is firstly separated and the skeleton geometry is extracted using sknw [21] in Python. Second, the middle points are recognized from the G and B channels. The main path boundaries are extracted from the black pixels, from which the external force geometry is reconstructed. Next, the internal vertexes are inferred from the skeleton geometry, middle points, and the main path boundaries. And finally, the internal triangulation is generated based on the internal vertexes in Rhino [22], and the entire force geometry is combined from the internal and the external force geometries. The final geometry should be a series of closed polygons that together becomes a convex network to meet the requirement of our graphic statics method.

A.11 Vector-based machine learning

The force diagram can only represent the topological information of the structural form, the geometric information (edge lengths for the form diagram) is still missing. Therefore, in order to learn and predict the edge lengths, we propose another vector-based machine learning model of Artificial Neural Network (ANN) (Figure S20). In the workflow of ANN, the dual diagram of the force geometry is first generated by the graphic statics method. Then for each edge in the dual diagram, a vector $(x1, y1, x2, y2, f)$ (Figure S20a) is generated, which represents the coordinates of the start and end points and the force magnitude (length for the corresponding edge in the force diagram). In addition, the corresponding edge length in the real form is found as the output the ANN (Figure S20b). Once trained, the ANN can predict the actual edge length for each edge in the dual diagram, thus helping generate the structural form using the graphic statics method.

The ANN [23] dataset contains 86412 pieces of data from 43206 edges in the dragonfly wing dataset, from which 82806 pieces are used as the training dataset and 3606 pieces are used as the testing dataset. All values are normalized into the range of 0 to 1 for better training so that we use Sigmoid function (Eq. 6) as the activation function and the mean squared error (MSE) function (Eq. 7) as the loss function.

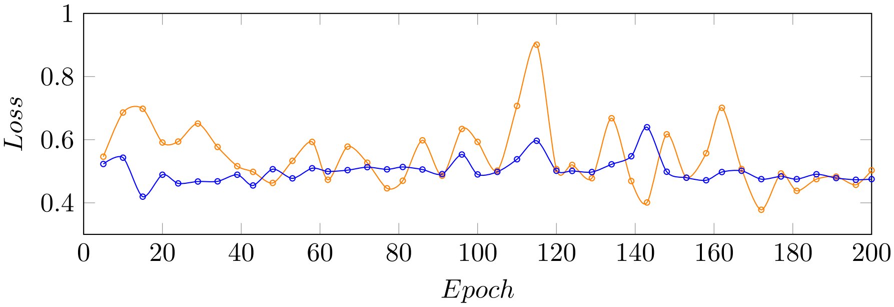

(a) Loss of model 1.

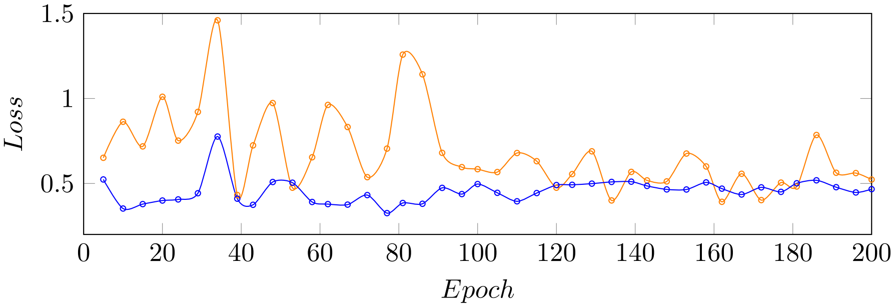

(b) Loss of model 2.

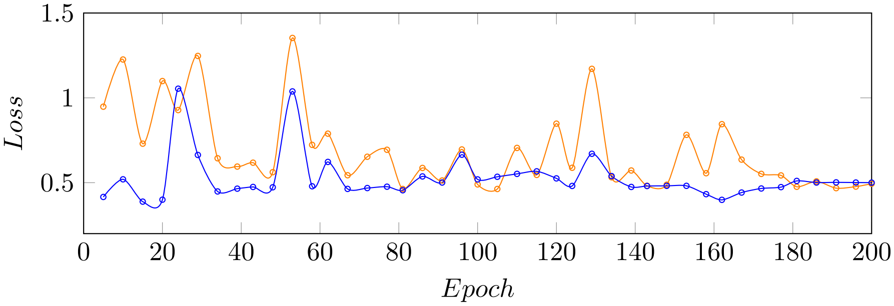

(c) Loss of model 3.

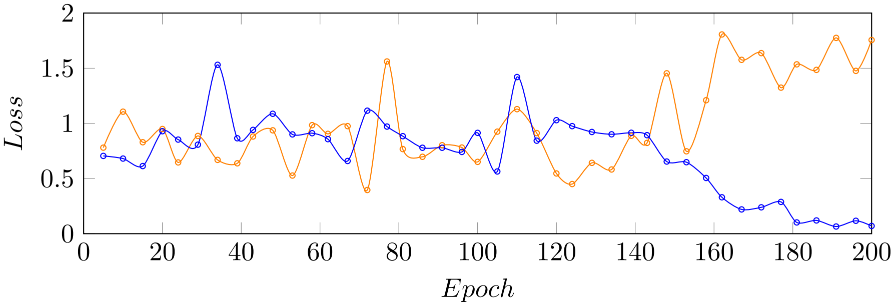

(d) Loss of model 5.

Figure S18: The loss of the Generator (orange) and the Discriminator (blue).

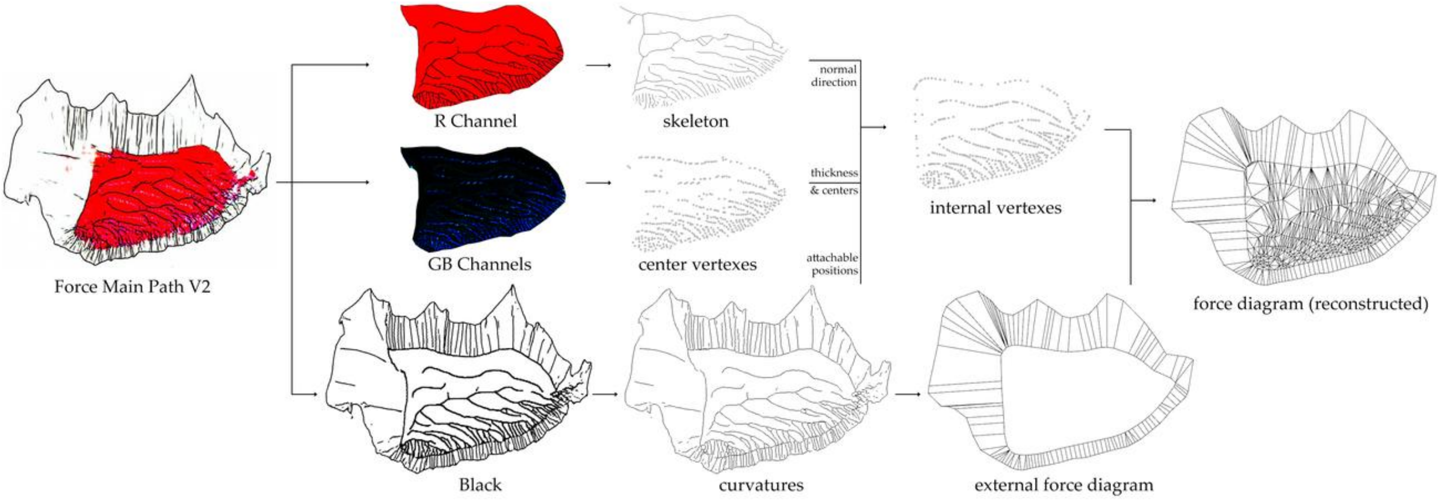

Figure S19: Post-processing to generate the force geometry of machine learning method 2.

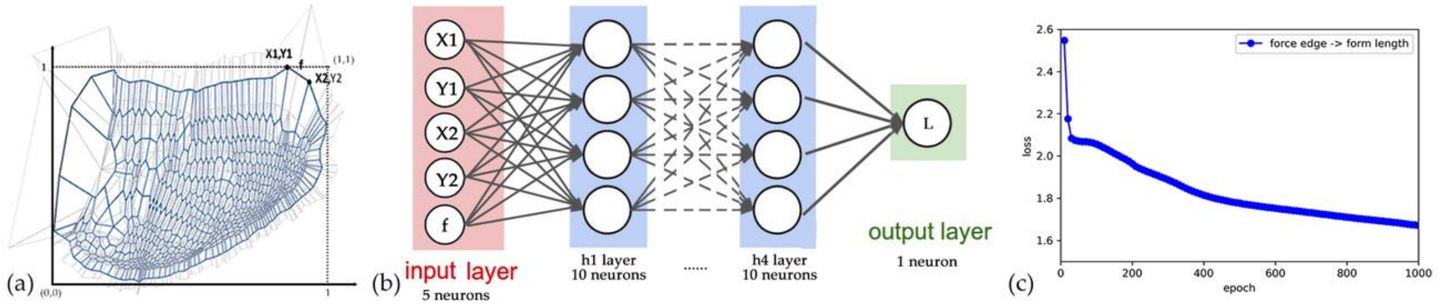

Figure S20: Details of the ANN: a) ANN dataset preparation; b) ANN structure. c) the loss history of the training process.

$$\hat{y} = Sigmoid(w * x + b) = \frac{1}{1 + e^{-(w*x+b)}} \tag{6}$$

$$Loss(y, \hat{y}) = \frac{1}{n} \sum_{i=1}^{n} (y_i - \hat{y}_i)^2 \tag{7}$$

With the training dataset above, in order to find the neural network settings with highest accuracy, the hyperparameters are fine-tuned for the training process while comparing the performance of each combination of hyperparameters. In each experiment, we use 5-Fold Cross-validation of the training dataset to evaluate the accuracy. Also, since the value of the output data is between 0 to 1, we apply the measurement [24] to compare the relative accuracy.

First, we compare the accuracy of other machine learning models with our ANN model (Table S3). In the initial hyperparameters of our ANN model, the number of neurons for each hidden layer is set as 10, according to a previous study on the suggestion of the ratio between the input and the hidden neurons [25]. The results show that the ANN model performs best with the accuracy of 97.15%.

Second, we adjust the number of layers to compare the accuracy of each ANN model (Table S4). It shows a highest accuracy of the model with 5 layers (4 hidden layers). Thus, the 5-layer ANN model performs best in our predictive task.

Third, Table S5 shows the comparison of the training results with different optimizers. According to the 5-fold cross-validation, we select Adam optimizer since it shows a highest accuracy with a fixed number of training epochs.

Table S3: Comparison of the accuracy of different machine learning methods.

	5-Fold Cross-validation Accuracy (%)
DecisionTreeRegressor	94.89
BaggingRegressor	95.64
RandomForestRegressor	95.64
ExtraTreesRegressor	95.63
LinearRegressor	97.01
**ANN**	**97.15**

Table S4: Comparison of the accuracy of ANNs with different numbers of layers.

	5-Fold Cross-validation Accuracy (%)
2-layer ANN	96.91
3-layer ANN	96.93
4-layer ANN	96.98
**5-layer ANN**	**97.15**
6-layer ANN	97.08
7-layer ANN	96.83

With Adam optimizer, we further change the learning rate and compare the performance of each model (Table S6). When the learning rate is set to 0.001, it results in a highest accuracy. Therefore, we set our ANN model with 5 layers and Adam optimizer with the learning rate of 0.001.

Therefore, the finalized ANN structure contains 4 hidden layers (5 layers in total) with 10 hidden neurons for each layer, 5 input neurons ($(x1, y1, x2, y2)$ for the coordinates of the vertexes and (f) for the edge length in the force diagram), and 1 output neuron (the edge length in the form diagram) (Figure S20a and b). We apply the entire set of the training data to train the final model. The loss history shows a convergence of the training (Figure S20c). The training process takes a CPU-only laptop several minutes to complete, with an accuracy of 97.8% for the testing dataset. Therefore, with the predicted edge lengths, the structural form is generated.

A.12 Experiment on the real loading scenario

From above experiments, we can find the capability of machine learning in generating the force diagram. Our force diagram is an abstract of the loading scenario, and from the perspective of a form finding tool for designers, it provides a solution with a certain degree of accuracy. However, as we mentioned before, the actual loading scenario is not exactly correct for the force diagram, the entire dragonfly wing might be a compression-tension-mixed situation, in which some members are bearing compression forces but some are in tension. One of the preconditions of our graphic statics method is that the entire system is either fully in compression or fully in tension. Although our final result shows the match of the generated structural form and the real structural form, the actual loading scenario is still unclear. Therefore, besides the success

Table S5: Comparison of the accuracy of ANNs with different optimizers.

	5-Fold Cross-validation Accuracy (%)
Adadelta	75.58
Adagrad	92.56
**Adam**	**97.15**
Ftrl	92.65
Gradient Descent	96.33
RMSProp	96.92

Table S6: Comparison of the accuracy of ANNs with different learning rates for Adam optimizer.

	5-Fold Cross-validation Accuracy (%)
0.0001	96.96
**0.001**	**97.15**
0.01	94.64
0.1	77.54

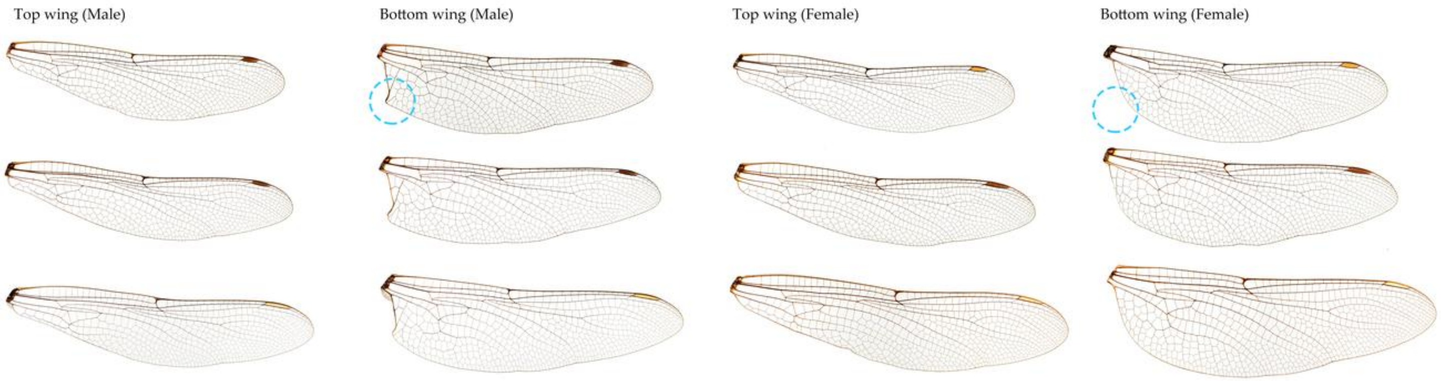

Figure S21: Categories of different dragonfly wings.

of our method as a form finding tool, we want to open the discussion of the actual loading scenario to see whether we can reach a conclusion of the real force pattern.

Therefore, we design the following experiment to explore the loading scenario (Figure S22). First, the dragonfly wing not only contains the internal funicular members, but also contains the membrane and the boundary ring, which can only bear tension forces. Therefore, we generate the triangulation of each cell in the form diagram as an abstraction of the membrane. Second, since we do not know whether each internal member is actually in compression or in tension, besides the compression-only force diagram for the internal members, we assume that the internal members might also bear tension force. Also, as the reacting forces for the compression-only internal forces, the boundary members of the dragonfly wing should also bear tension. Thus, the internal members, the triangulation of the membrane, the boundary ring, and the reacting forces as additional geometries can be merged as a tension-only form, which works together with the compression-only internal form.

With this new tension-only form, we apply our graphic statics method to generate the force diagram, under the condition that the reacting forces should be the same as the corresponding members in the internal force diagram, thus we use the force boundary of the internal force diagram as the boundary constraint for the tension-only force diagram. This operation ensures the reacting forces from the compression-only form and the tension-only form match with each other, while the rest members in the tension-only form can be transformed into the corresponding force diagram. However, noted that the Geometric Degree of Freedom (GDoF) [15] of the tension-only form diagram is 956 (larger than 0), which means the structure is very indeterminate and there are an infinite number of force diagrams that can be generated and satisfy the graphic statics rules. Therefore, the generated force diagram only represents one of the solutions, and the actual situation can be different and more complex.

Last, we map the edge lengths in the force diagram to the structural thickness in the form diagram, and generate the structural form for the tension-only part. Then we combine the two structural forms together and generate the entire structural form, which shows a mixed condition of the tension and compression patterns. However, when we compare the combined structural thickness with the real structural thickness, they clearly do not match. To verify this phenomenon, we further also develop an algebraic solution to calculate the tension and compression status after removing the virtual loads. The result also shows a

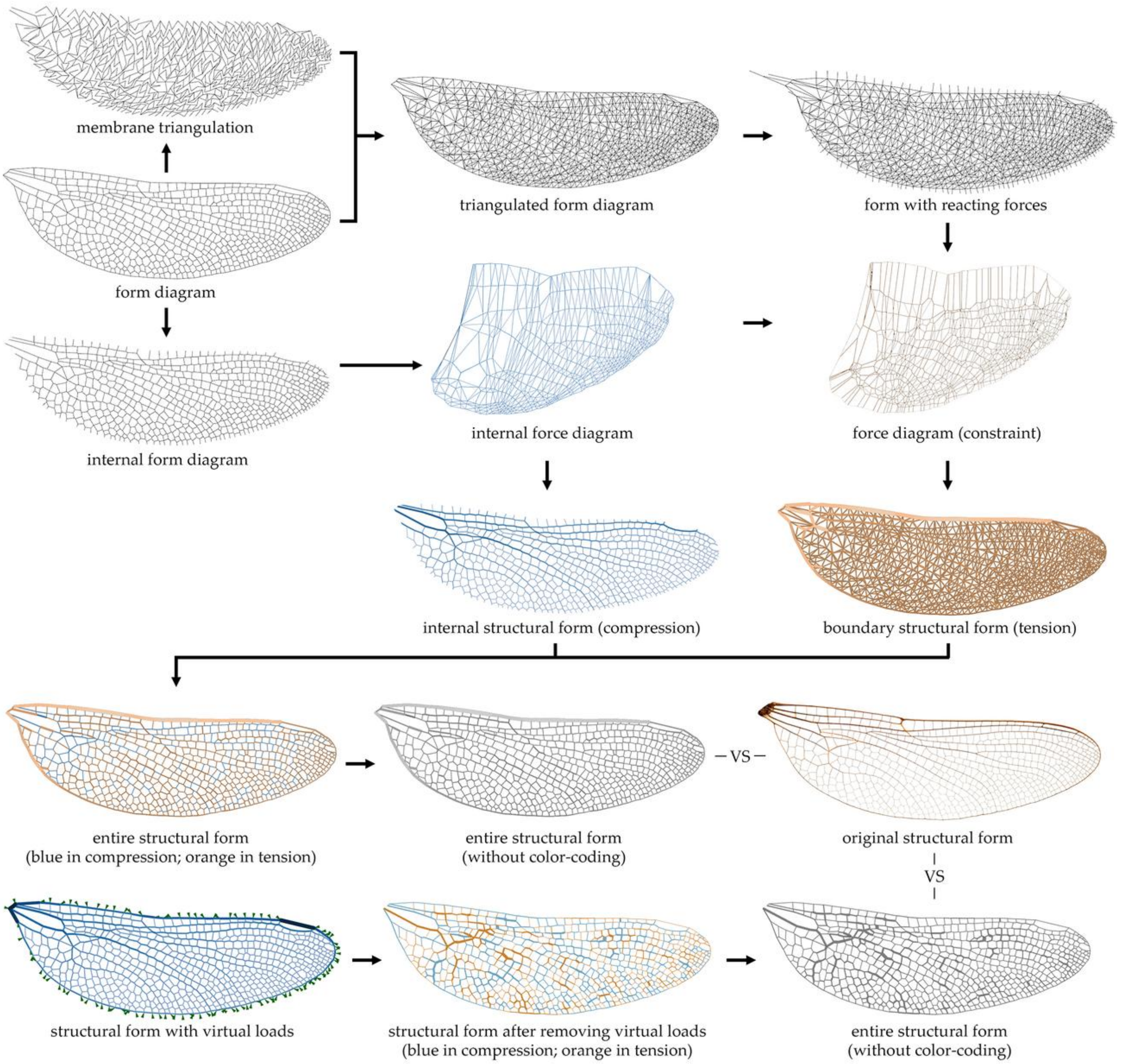

Figure S22: Exploring the actual loading scenario of the dragonfly wing. We abstract the membrane and the boundary ring as triangulated members, which can only bear the tension force. By merging the tension-only membrane and boundary ring with the compression-only internal structure, the combined structural form shows a complex compression-and-tension pattern. Further, we develop an algebraic solution to calculate the tension and compression status after removing the virtual loads. It also shows a complex compression-and-tension pattern.

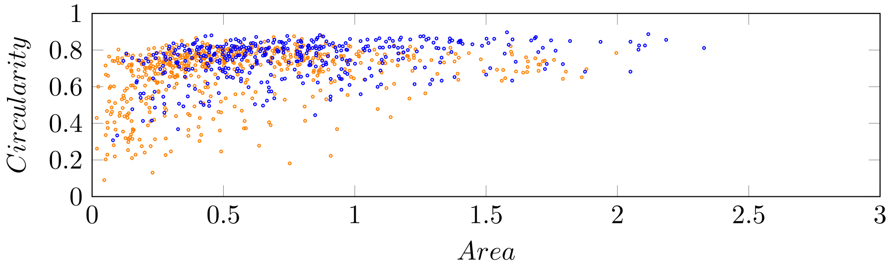

(a) Scatter plot for the grasshopper wing.

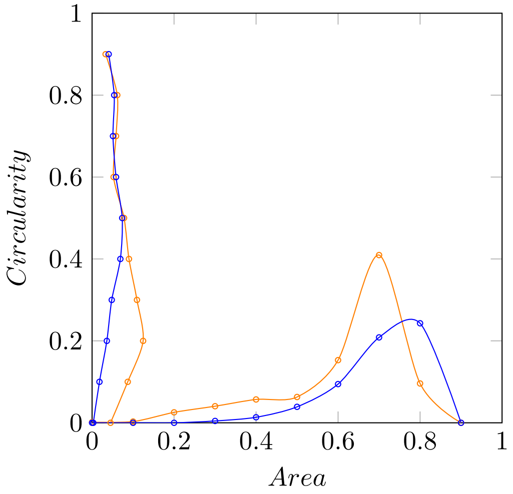

(b) Distribution map for the grasshopper wing.

Figure S23: The plot of the area and the circularity of each polygon for the real grasshopper wing (orange) and the generated grasshopper wing (blue).

mixed condition of the tension and compression patterns. And the generated structural thickness does not match the real wing.

Therefore, we regard this result as a conclusion that the actual loading scenario and the force pattern are more complex than we thought, it is hard to identify the compression-and-tension status of each structural member in the dragonfly wing. Therefore, we conclude that the actual force diagram with the real loading scenario is uncertain and unable to be learned by machine learning. The previous force diagram which abstractly represents the structural thickness for machine learning is suitable to reveal the morphological properties of the dragonfly wing, as that our method is a form-finding tool for designers.

A.13 Result comparison of other species

See Figure S23, S24, and S25.

A.14 Web-based design tool

We develop a web-based tool that accepts user inputs and feedbacks generated structures. The web tool is implemented as an online resource and open for designers to visit as a web page. Meanwhile, a local server proceeds with the input data, generates the output structure, and sends the model file to be displayed on the web page. With this web tool, designers can easily adjust the input parameters and boundary conditions, and obtain the structure model online, without going through the complex local computing process.

To be specific, Figure S28 shows the workflow of the web tool, which contains the frontend and backend. It follows four steps: 1) The user inputs the boundary and parameters in the web page, and HTML and

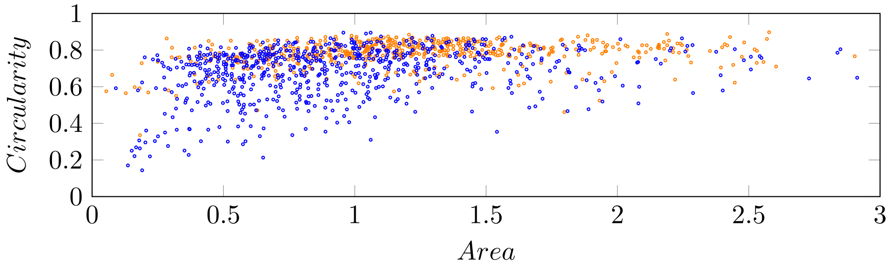

(a) Scatter plot for the amazon water lily.

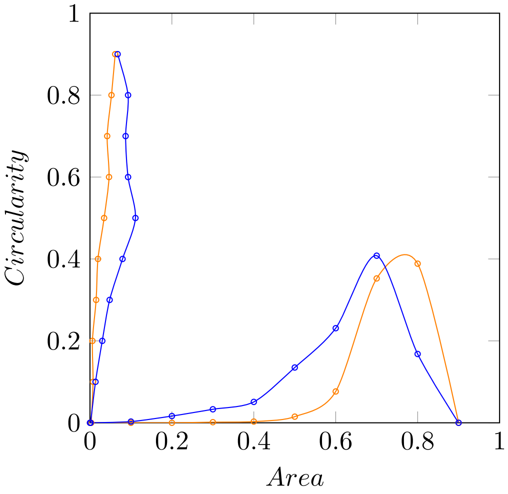

(b) Distribution map for the amazon water lily.

Figure S24: The plot of the area and the circularity of each polygon for the real amazon water lily (orange) and the generated amazon water lily (blue).

Javascript [27] transform the data from Canvas [28] into a formatted database; 2) The server stores and proceeds the data [29, 30], and updates an indicator file; 3) The geometric components [22, 31] and the machine learning components [32, 33] in the server work together to generate the structural model as CSV file, and send it back to the web page; 4) the web page [34] displays the model, and allows the user to generate and download the STL file.

First, the web page should receive the input boundary and parameters from the user. Figure S29 shows the icons, names, ranges, and buttons in the "Input Control Panel". The boundary is defined as a closed polygon of several vertexes. The user can select the number of vertexes and adjust the positions of each vertex by dragging them on the canvas. The boundary vertexes are transformed into numeric values based on their coordinates. Noted that, the first line in the boundary is defined as the anchor position, thus additional marks and geometries are drawn and shown to the user to identify the anchor.

Besides, the "Input Control Panel" also includes parameters that are defined as numeric values. A set of six parameters can be input from the user and sent to the server. To be specific, "Subdivision Density" defines the density of the structural members. By increasing it, more members will be generated with a longer time cost. "Sharpness" defines the upper and lower bounds of the length constraint for each edge. Increasing it will give more freedom to the geometric generation process in graphic statics. "Length Constraint Multiplier" defines the relaxation of the edge length constraints. Increasing it will cause more rectangular cells than circular cells. "Boundary Constraint Magnitude" defines the magnitude of the boundary constraint to the form. Increasing it will make the structure attach closer to the boundary. "Iterations" defines the number of iterations in the geometric generation process in graphic statics. Increasing it will generate a more accurate structure but with a longer time. "Total Length of the Wing" defines the size of the generated model in millimeters. Last, the user can select the machine learning model

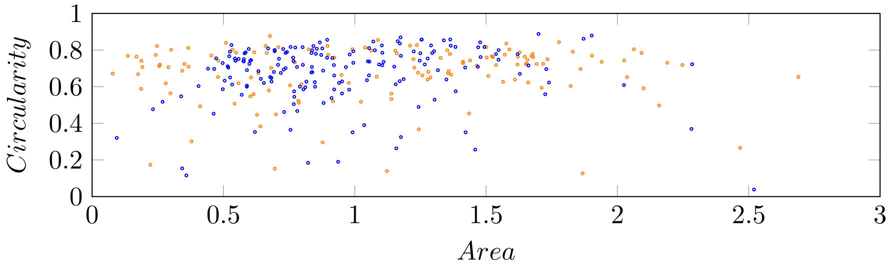

(a) Scatter plot for the damselfly wings.

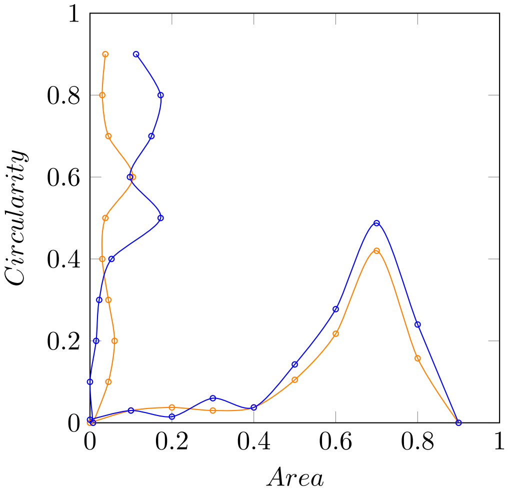

(b) Distribution map for the damselfly wings.

Figure S25: The plot of the area and the circularity of each polygon for the real damselfly wings (orange) and the generated damselfly wings (blue).

of different species, the default is set as the dragonfly wing model.

Noted that, the web page will restore the input parameters when the user successfully submitted the last time, thus the user can more easily adjust the parameters. There is a button "Default Settings", by clicking it, the input parameters will be set as the default values. If a new user does not understand the meaning of each parameter, he/she can move the mouse cursor to the button of each parameter to see its name and click the "help" or "about" button to see the detailed instructions.

When finishing adjusting the six input parameters, the user can click the "Submit" button in the "Compute" panel to send the first set of input parameters to the server. Figure S30 shows the algorithm in the backend and the frontend of the server after the user submits the input parameters. First, in order to avoid duplicated submissions from multiple users before the server completely responds to the current request, an indicator value in the server is firstly loaded by the web page. If it indicates that a request is being proceeded, a warning message will be shown to the user when he/she clicks the "Submit" button, and no values will be submitted to the server. Besides, the entire generation process in the server usually takes three to seven minutes. The web page will be automatically refreshed every 10 seconds until it receives the result from the server.

When the server finishes the generation process, it will send back a CSV file to the web page, which contains the geometric information of the generated structure, the graph information for implementing the Minkowski sum, and the numeric information of the FEM analysis. The geometric information contains the coordinates of the start and end points of each edge, as well as its corresponding force magnitude. The graph information stores the connectivity matrix of the form and the force diagrams. The FEM analysis result contains the deformation magnitude for each edge in the structure.

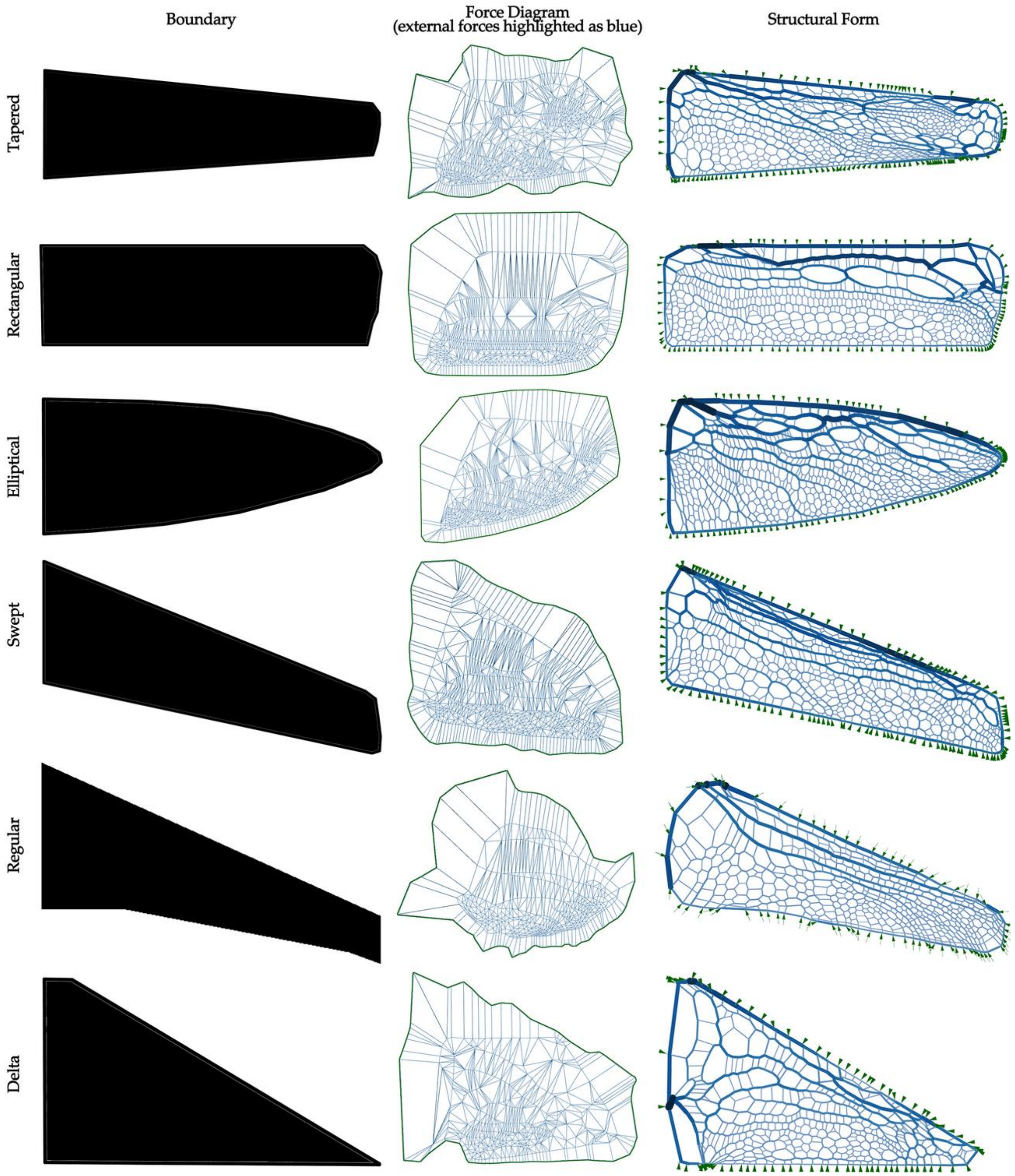

Figure S26: Boundaries [26], force diagrams, and form diagrams of the generated airplane wings. (The virtual loads are highlighted as green.)

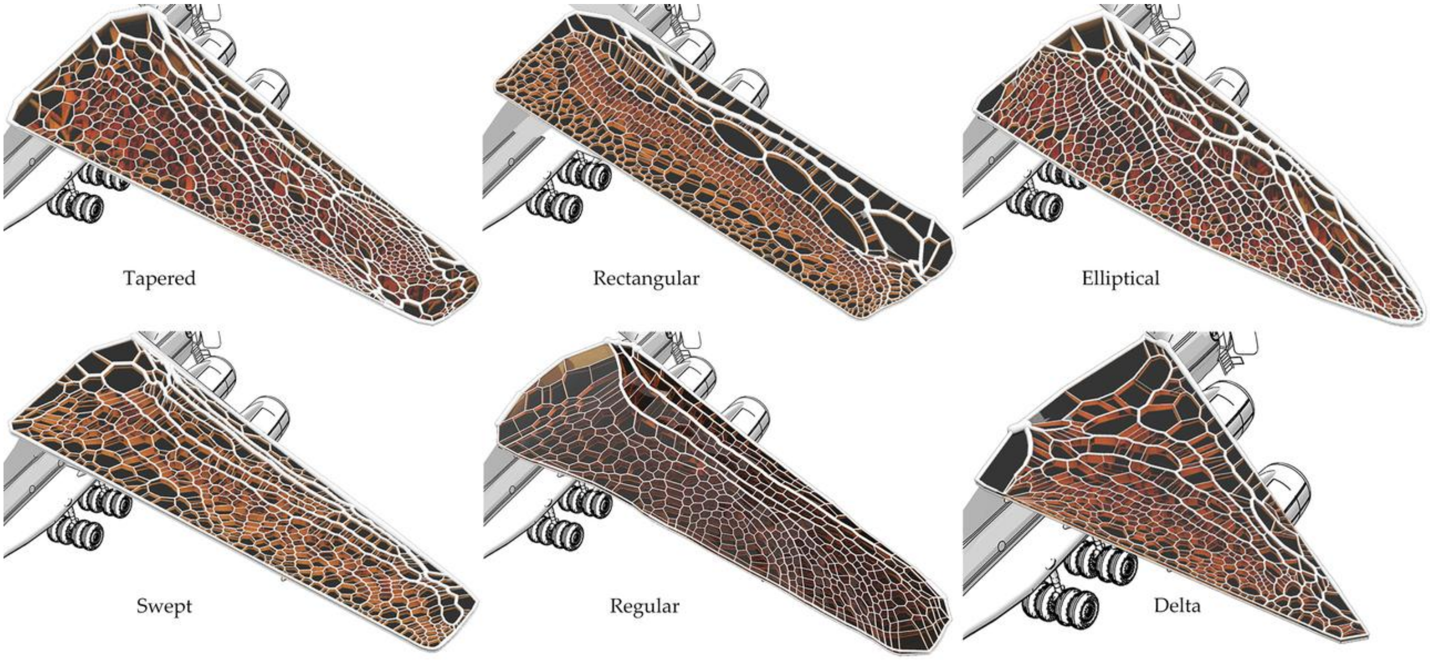

Figure S27: The renderings of the generated airplane wings.

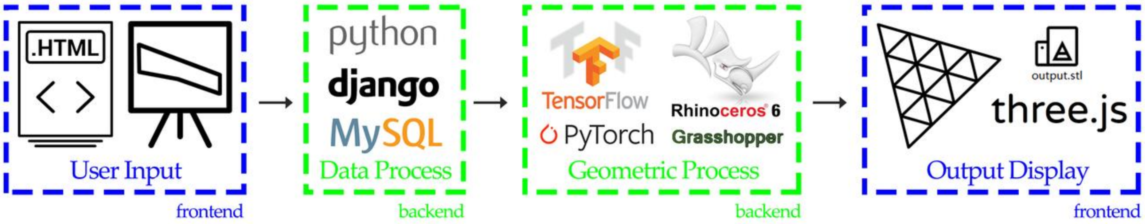

Figure S28: The workflow and data flow of the web tool.

Icon	Name and range	Icon	Name and range
	Input Boundary (canvas input)		Subdivision Density (0.01-1.00)
	Sharpness (0.00-1.00)		Length Constraint Multiplier (0.01-2.00)
	Boundary Constraint Multiplier (0.01-9.99)		Iterations (10000-30000)
	Total Length of the Wing (10-50000)		Machine Learning Model (selection)
	Default Settings (button)	Submit	Submit (button)
help	Instruction on how to use (button)	about	Developer information (button)

Figure S29: The control parameters and functional buttons in the user input panel and the compute panel.

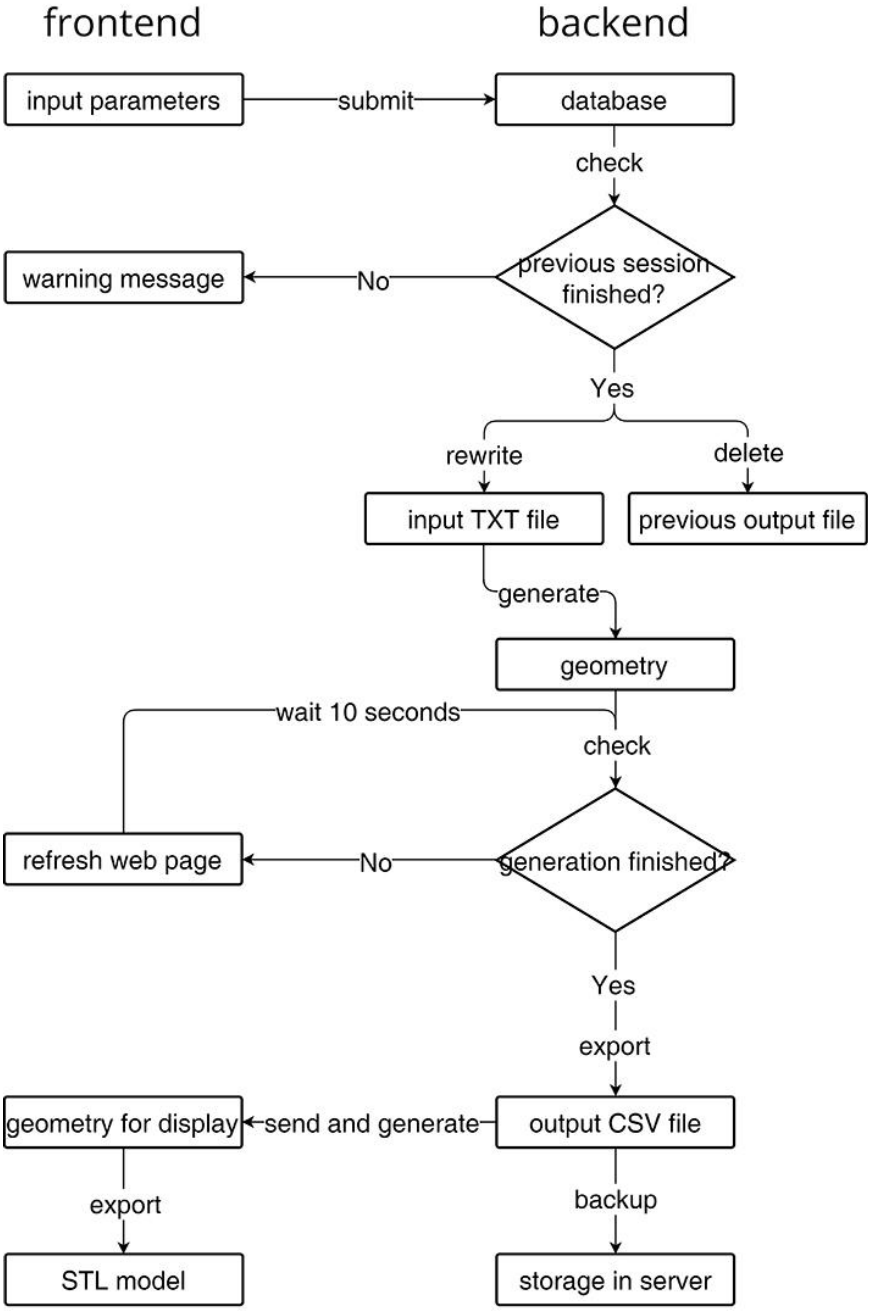

Figure S30: The workflow in the backend and the frontend of the server after the user submits the input parameters.

Icon	Function	Icon	Function
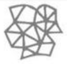	View the generated model	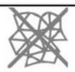	Remove the generated model from the scene
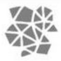	View the Minkowski Sum	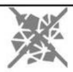	Remove the Minkowski Sum geometry from the scene
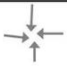	Show the external forces	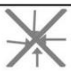	Hide the external forces
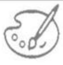	Show color-coding	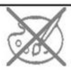	Hide color-coding
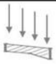	Turn on the FEM result under the load of self-weight	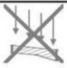	Turn off the FEM result under the load of self-weight
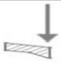	Turn on the FEM result under the point load on the farthest vertex	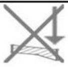	Turn off the FEM result under the point load on the farthest vertex
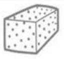	Material of the Wing (selection)	FEM	Request the recomputation of the FEM results from the server

Figure S31: The functional buttons in the output control panel and the FEM control panel.

Next, when the web page receives the data file, the user can choose a different display mode in the "Output Control Panel" and "FEM Control Panel" (Figure S31) to turn on or off the generated model and the Minkowski Sum. Also, the user can turn on or off the FEM results under the load of self-weight or under the point load in the farthest vertex.

In the case of the normal display mode, the web page regenerates the structural members according to the information from the file and the second set of the user input parameters of the minimum radius and the maximum radius. An additional transparent box geometry is shown to indicate the anchor of the structure. The user can control the camera with the mouse in the main display window to better view the generated model. The generated 3D model is displayed on the web page with pre-set lighting environment. However, to reduce the computational load from the local device, the shadow is represented as a series of static geometries on the ground with gray lines. The color setting for the main geometry keeps constant with that in PolyFrame. In the display control panel, the user can also change to turn on or off the display of the external forces.

In the case of the Minkowski Sum mode, the web page reads the user input of the Minkowski Sum indicator (MSI) and calculates the corresponding status in the form-to-force transformation. The graph information in the feedback file contains the following items: 1) the coordinates of the vertexes in the form diagram; 2) the index of neighbor cells of each cell in the force diagram; 3) the index of the shared edges in the neighbor cells of each cell in the force diagram; 4) the index of edges in each cell; 5) the coordinates of the start and end points of each edge in the force diagram. By scaling the cells in the force diagram with the MSI value and moving them to each corresponding vertex in the form diagram, each edge in the force diagram will become an area with thickness. Therefore, with a gradually-changed MSI value from 0 to 1, the areas shift from the edges in the form diagram to the edges in the force diagram, thus showing the transformation between the form and force. Still, the user can turn on or off the external forces in the Minkowski Sum mode.

For the FEM analysis, Karamba [35] is used to calculate the deformation of edges based on the user input of the span and the material of the structure. Applicable materials include steel, wood, concrete, and

Icon	Function	Icon	Function
STL	Export the current scene as an STL model	1	Restore the pre-generated sample 1 from the server
CSV	Download the vector-based data file to save the result	2	Restore the pre-generated sample 2 from the server
CSV	Upload the vector-based data file to restore the result	3	Restore the pre-generated sample 3 from the server

Figure S32: The functional buttons in the file manager panel.

aluminum, and the material property is embedded in Karamba. We provide two types of loading: 1) self-weight loading for all vertexes; 2) point loading for the farthest vertex to the anchor with the magnitude of half of the self-weight. In the CSV data file, the FEM analysis part includes the following information for each edge: 1) coordinates (x,y,z) for the start and end points (deformation in Z axis included); 2) color-coding value (R,G,B). The user can adjust the multiplier in the frontend to increase or decrease the deformation magnitude, and view the color-coded edges and color scales. When the user changes the material setting or the structural thickness, a recomputation request can be sent to the server and the FEM results will be updated in round ten seconds.

In addition, to better help users restore the previously generated results, in the “File Manager” panel (Figure S32), users can download the data as a CSV file and save it on their local computer. If the user inputs an email address when submitting a request to the server, when the computation is finished, the server will send an email to the user with the CSV data file attached. By uploading the file to the web page, users can restore the input parameters, the output structure, the Minkowski Sum geometry, and the FEM analysis results. Restoring the previous result only requires the local computer from the user, thus it is an offline process and does not require a connection to the server. Also, the user can export and download the STL file for 3D printing or other purposes.

Therefore, with this web page implementation http://www.ai-gs.com/frontend/DFW-GH.html [36], users even without much knowledge of machine learning and graphic statics could easily generate lightweight and high-performance structures within given boundaries. Figure S33 shows the web page with control panels unfolded, and Figure S34 shows the web page with control panels folded. The user can freely decide to fold or unfold each panel to better balance the UI and the model display.

Next, several cases are generated and shown using our web tool. The user can either submit multiple requests to our backend server to generate structures with different input-related parameters or adjust the output-related parameters in the frontend to view and export results.

In the first case (Figure S35), structures with different input boundaries are generated. The user can select the number of control points in the input boundary, and drag the points to adjust their positions. Even if the user inputs an invalid boundary such as crossing curvatures, our geometric script will merge it into a pixel-based black-and-white image, and send the image to machine learning models. In addition, the user is not required to input a wing-like boundary. As long as the anchor is on the left, any cantilever structure can be generated with the input boundary, which contains the features of dragonfly wings.

The second case shows one example of controlling other input parameters such as the subdivision density (Figure S36). Among the input parameters, the subdivision density is the most important one since it directly controls the complexity of the generated structure. A smaller subdivision density can significantly simplify the structure while keeping the features of dragonfly wing patterns. In our recent research, we

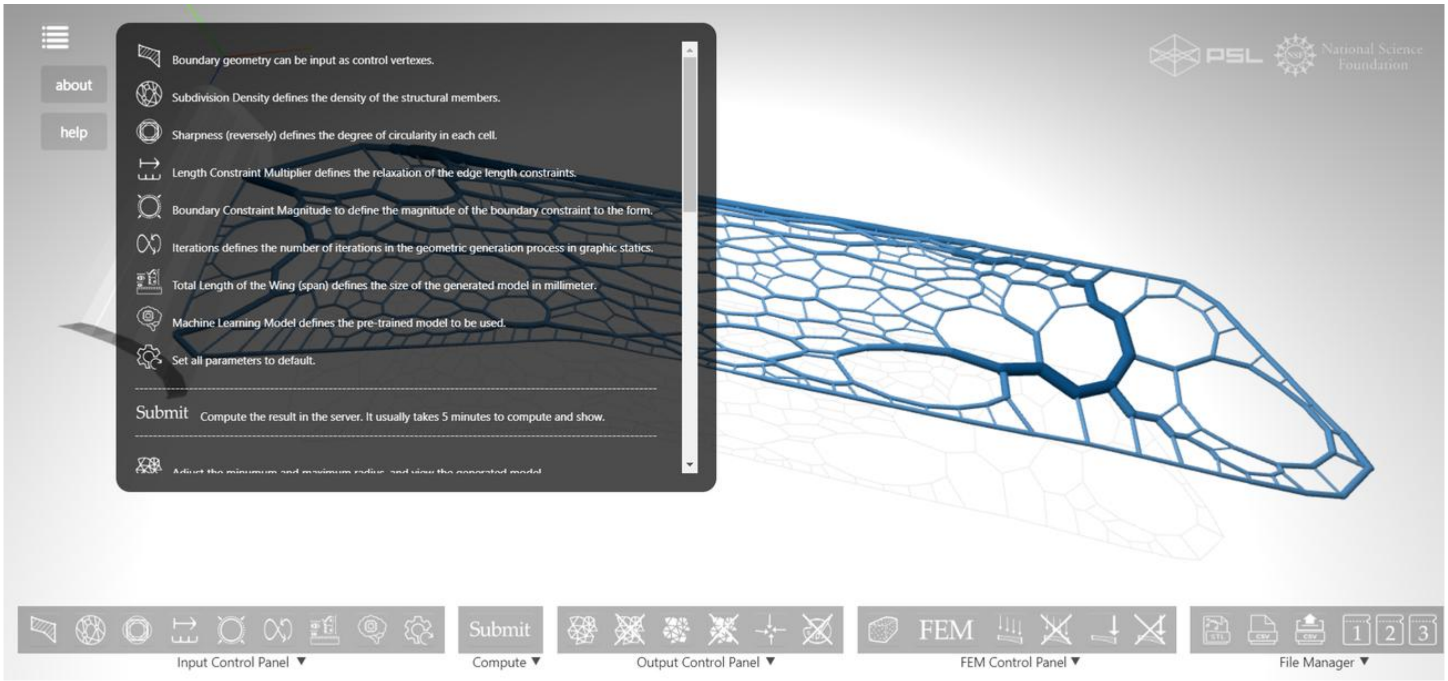

Figure S33: The implemented web page with panels unfolded.

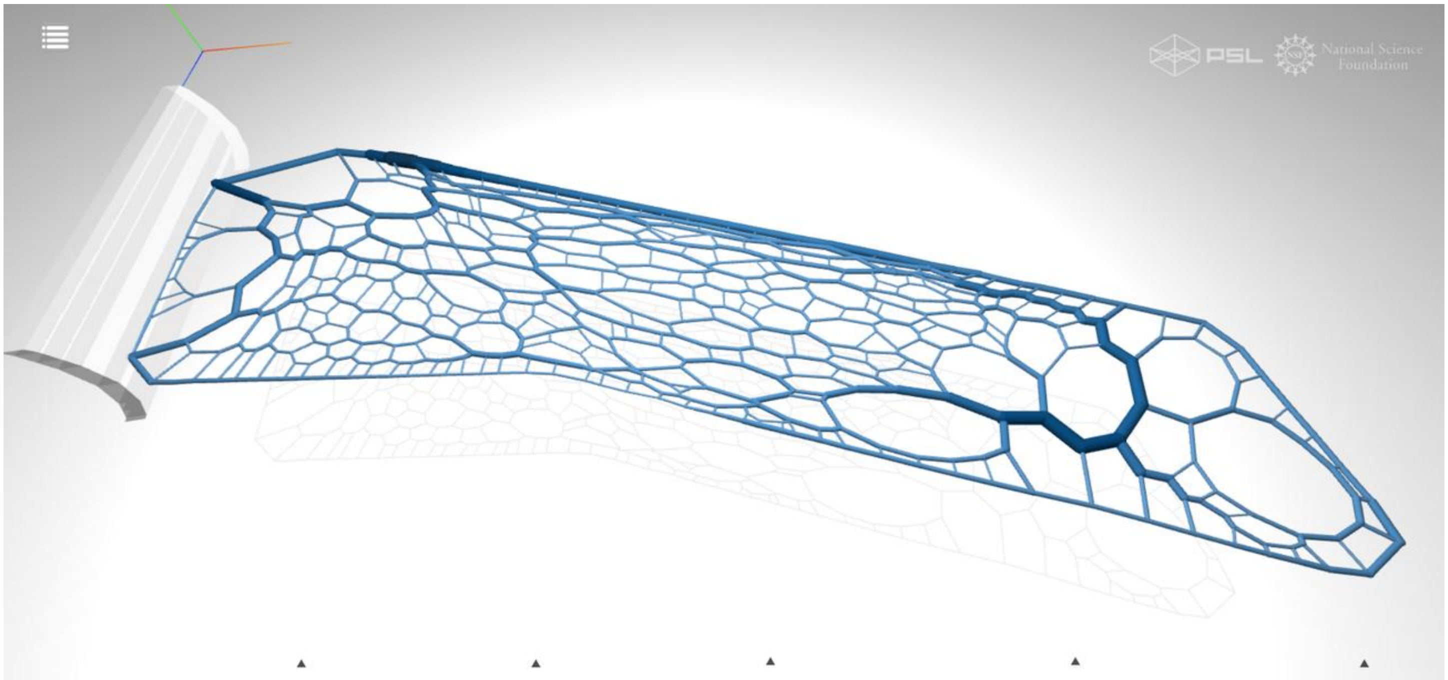

Figure S34: The implemented web page with panels folded.

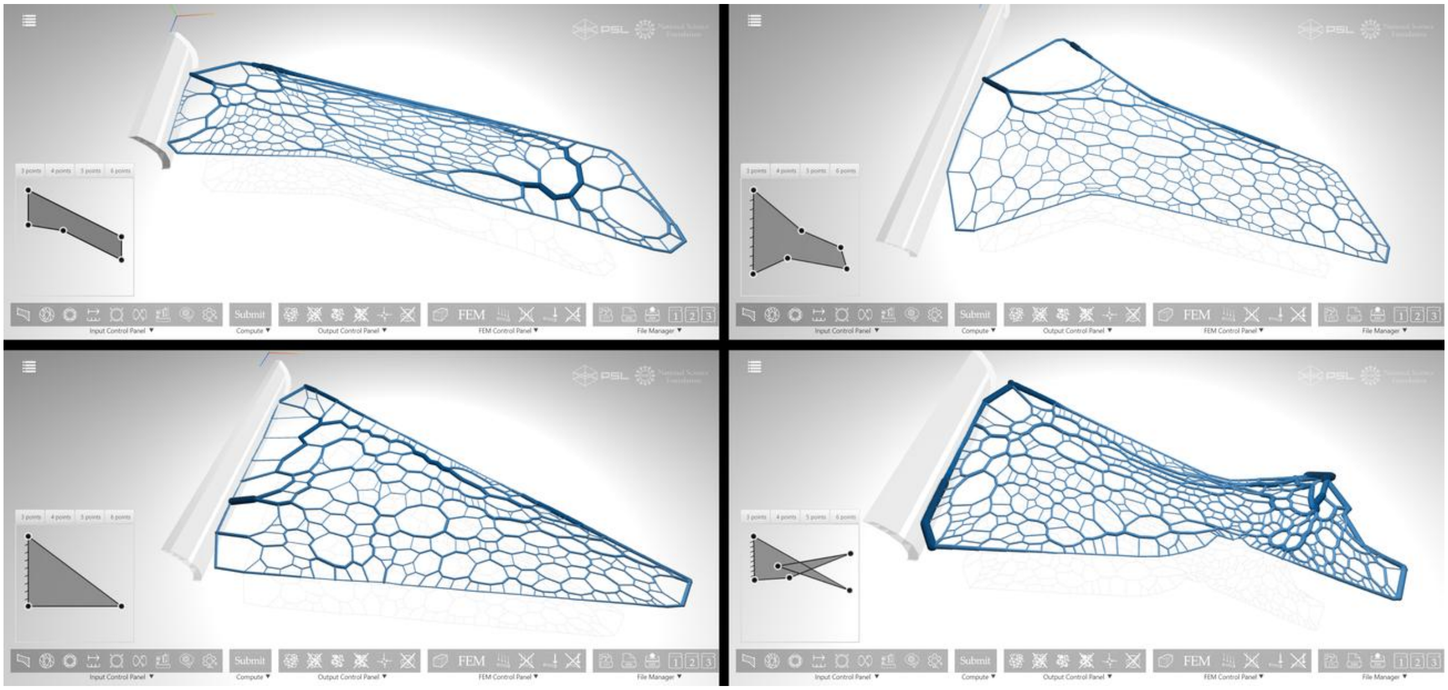

Figure S35: The generated structures with different user-input boundaries.

found that simplifying the structure to a certain degree would increase the structure performance, and make it easier to fabricate in the real world. Thus, the user can consider the fabrication ability and adjust the subdivision density. Other parameters such as sharpness and iterations can also greatly affect the generated result, but changing the input parameters requires a re-computation from our server and it usually takes around five minutes to respond, including the time cost for machine learning, geometric algorithm, data generation, data loading and web display.

Besides changing the input parameters, the user can also adjust the output display modes and the related indicators to show or hide the generated structures and the analytical results. Figure S37 shows the case of Minkowski Sum in different stages with different values of the indicator. The indicator defines the position in percentage of the current Minkowski Sum in the form-to-force transformation. The result is closer to the form diagram with a smaller value of the indicator, while it is closer to the force diagram with a larger value of the indicator. The user can adjust the value of the indicator and view the smooth transformation from the form to the force.

Also, Figure S38 shows the FEM results under different loading scenarios and materials. As mentioned, in the geometric mechanism of our server, we provide the FEM results of the deformation under the self-weight or a point load. The user can decide to show none/one/both of them by clicking the corresponding buttons in the output control panel, or hide the main structure to better compare the FEM results. The color-coded scale is also shown on the right of the web page when the corresponding FEM result is shown. The scale includes the minimum and maximum values of the percentage of deformation compared with the span, and the real values of the deformation in millimeters. When keeping the deformation multiplier constant, the user can also directly compare the FEM results from different structures.

The final case shows the application of our web tool in exporting the result to other platforms for various purposes. In the normal display mode, the user can adjust the minimum and the maximum radius to control the range of the thickness for edges, and export the structure as an STL model (Figure S39). The exported STL model can be imported to a variety of platforms, including modeling software for further analysis and 3D printing software for digital fabrication. Therefore, our web tool completes the logic of loop by accepting the user input and exporting the generated result back to the user.

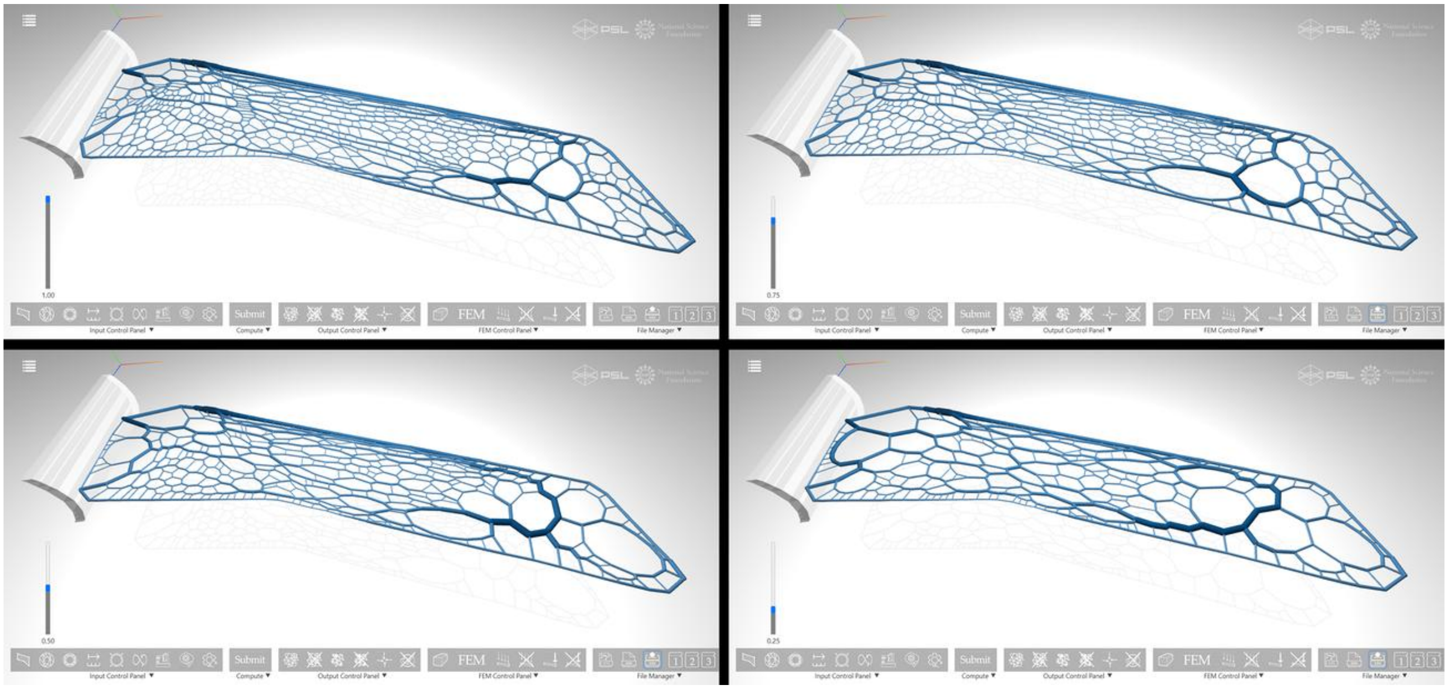

Figure S36: The generated structures with different subdivision densities. Top left: SD=1.0. Top right: SD=0.75. Bottom left: SD=0.5. Bottom right: SD=0.25.

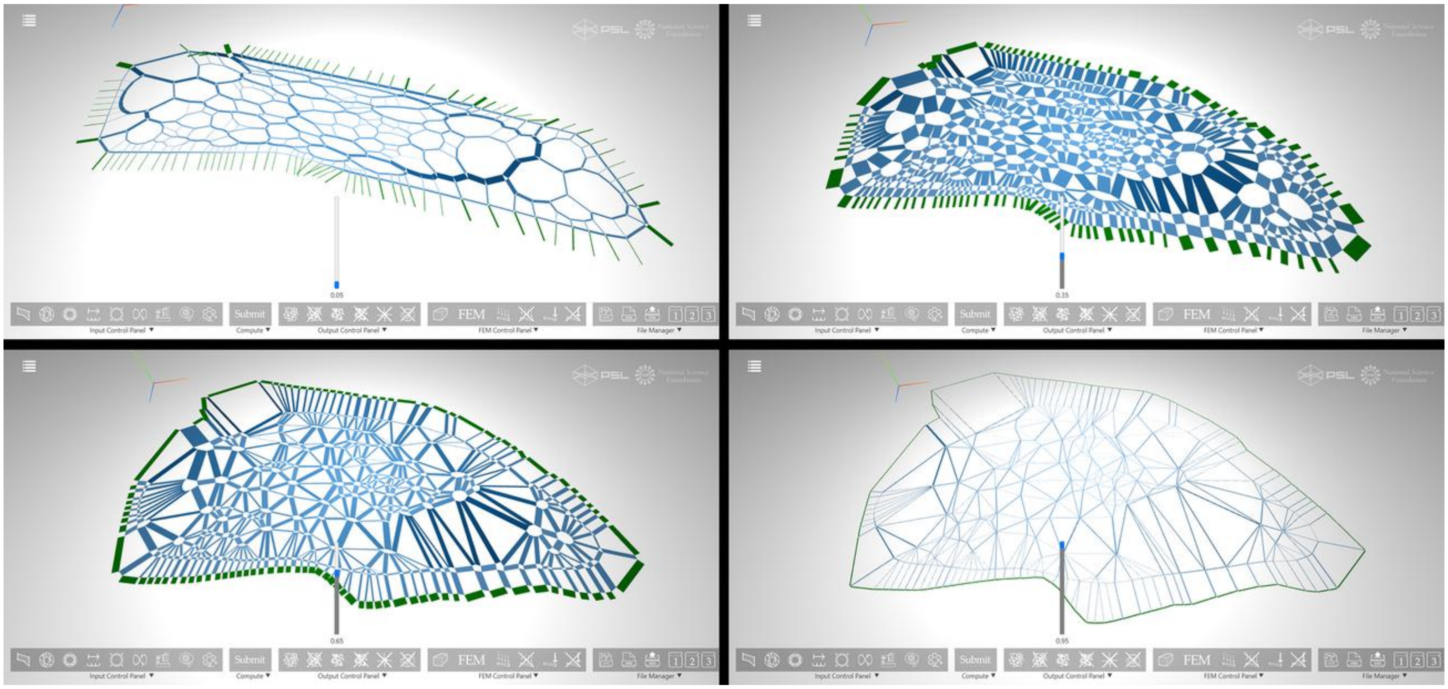

Figure S37: The generated Minkowski Sum with different stage indicators. Top left: MSI=0.05. Top right: MSI=0.35. Bottom left: MSI=0.65. Bottom right: MSI=0.95.

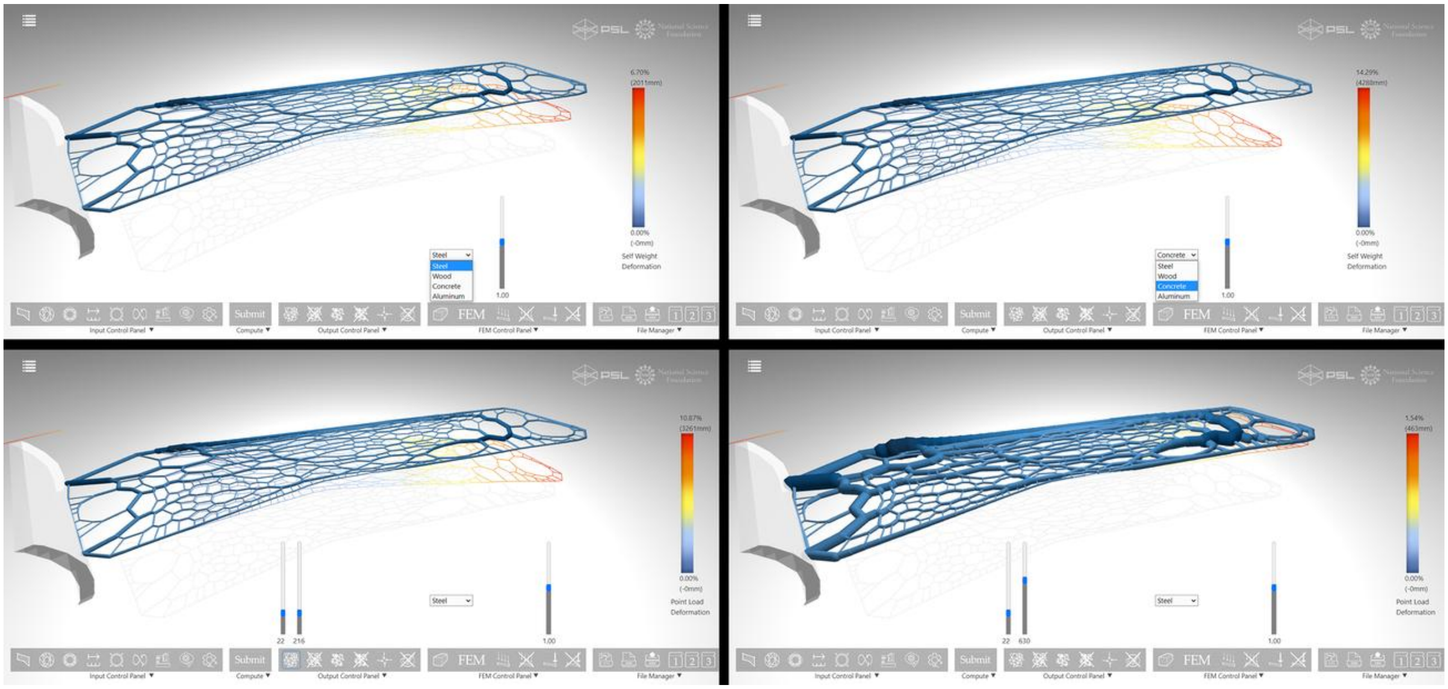

Figure S38: The FEM analysis results with different loading scenarios, materials, and structural thickness. Top left: self-weight load with steel material. Top right: self-weight load with concrete material. Bottom left: point load with steel material. Bottom right: point load with steel material and larger structural thickness.

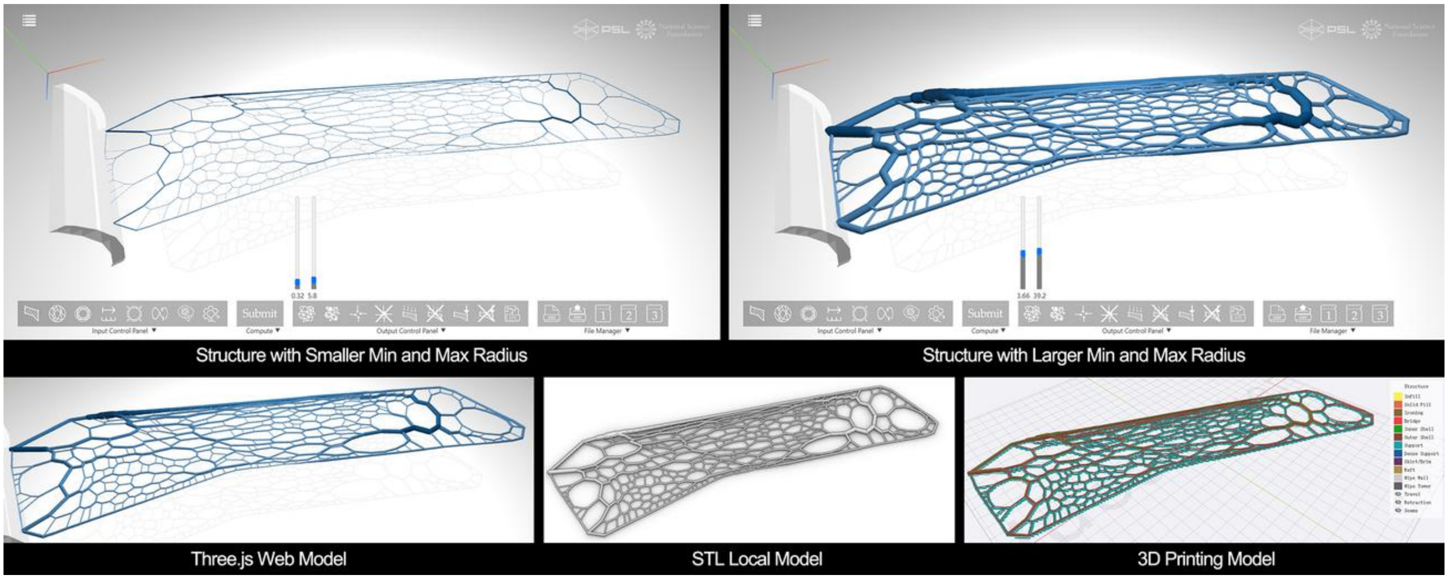

Figure S39: Top: Structures with different minimum and maximum radii. Bottom: Exported STL model and its 3D printing preview.

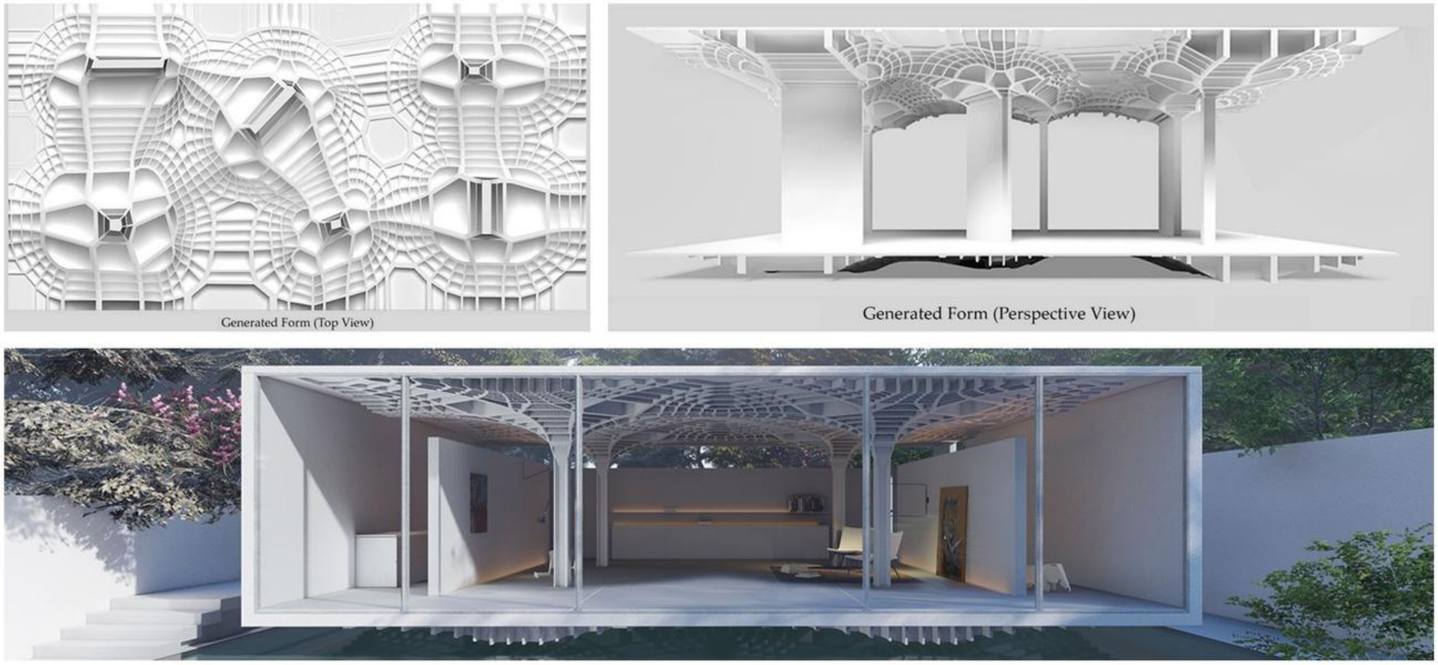

Figure S40: An example of cantilever structures that could be generated by our method.

A.15 Design application examples

See Figure S40.

A.16 Comparing the real and the generated wings

To compare the ML-assisted generated wing with the actual dragonfly wing, mode shapes, natural frequencies, and out-of-plane stiffness of two wings under uniform pressure have been studied. Our introduced designed method is capable of generating vain patterns, therefore veins are modeled accurately in the simulation. Although the patches, which enclose the in-plane space between veins may have different thicknesses, for a fair comparison of the performance of veins' pattern we assume an average value for the patch ($\frac{t^{patch}}{L^{wing}} = 4*10^{-4}$, t and L are patch thickness and wing's length, respectively). Results in Figure S41a show that more veins are participating in the transferring load (more red veins in the stress contour) in the generated wing, which leads to less red part in the deformation contour. According to the results, the generated pattern is 18% stiffer than the actual dragonfly wing (Figure S41c). Mode shapes and natural frequencies are also important properties of the wing. Mode shapes of the actual and generated design are very similar, which means generated wing is very similar to the actual wing in terms of behavior. However, generated patterns with higher stiffness and better mass distribution have significantly higher natural frequencies (more than 30%).

A.17 Designing the structural testing samples

In the design of the testing samples for the loading and wing tunnel tests, we extract the boundary from an airplane wing, and generate our wing structures within the same boundary (Figure S42). In our generated wings, there are two parameters that act to generate 8 different wings. They are: 1) the subdivision density, which ranges from 0 to 1 and defines the percentage of nodes remaining in the force diagram. Therefore, a smaller value of the subdivision density will reduce a larger number of structural members, which are re-generated based on the remained nodes in the force diagram. 2) range of the structural thickness, which defines the lower and upper bounds of the thickness of the structural members.

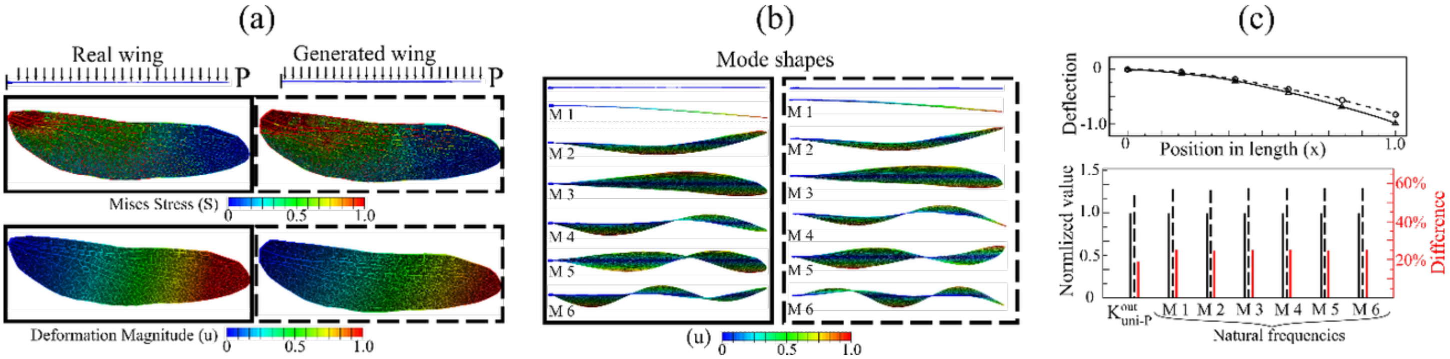

Figure S41: Mechanical properties of the real and generated dragonfly wings: (a) Von-Mises stress, and magnitude of displacement contour under uniform pressure (values are normalized to the maximum value in results of the real-wing), (b) Mode shapes, and (c) Deflection of the wings under uniform pressure and normalized out-of-plane stiffness and natural frequencies (dash lines and solid lines represent generated and real wings).

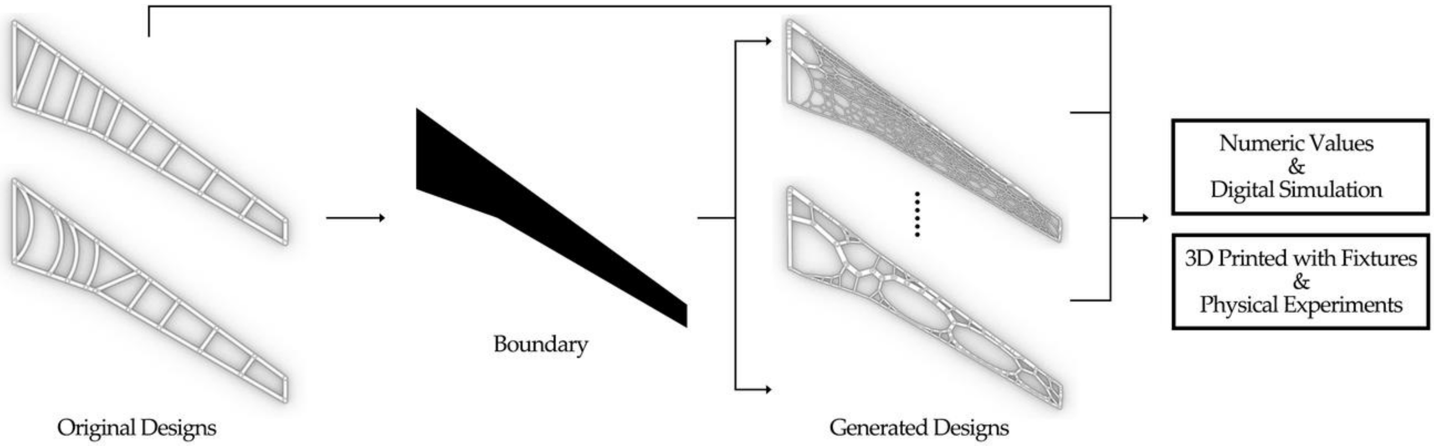

Figure S42: The design process of the airplane wings for testing.

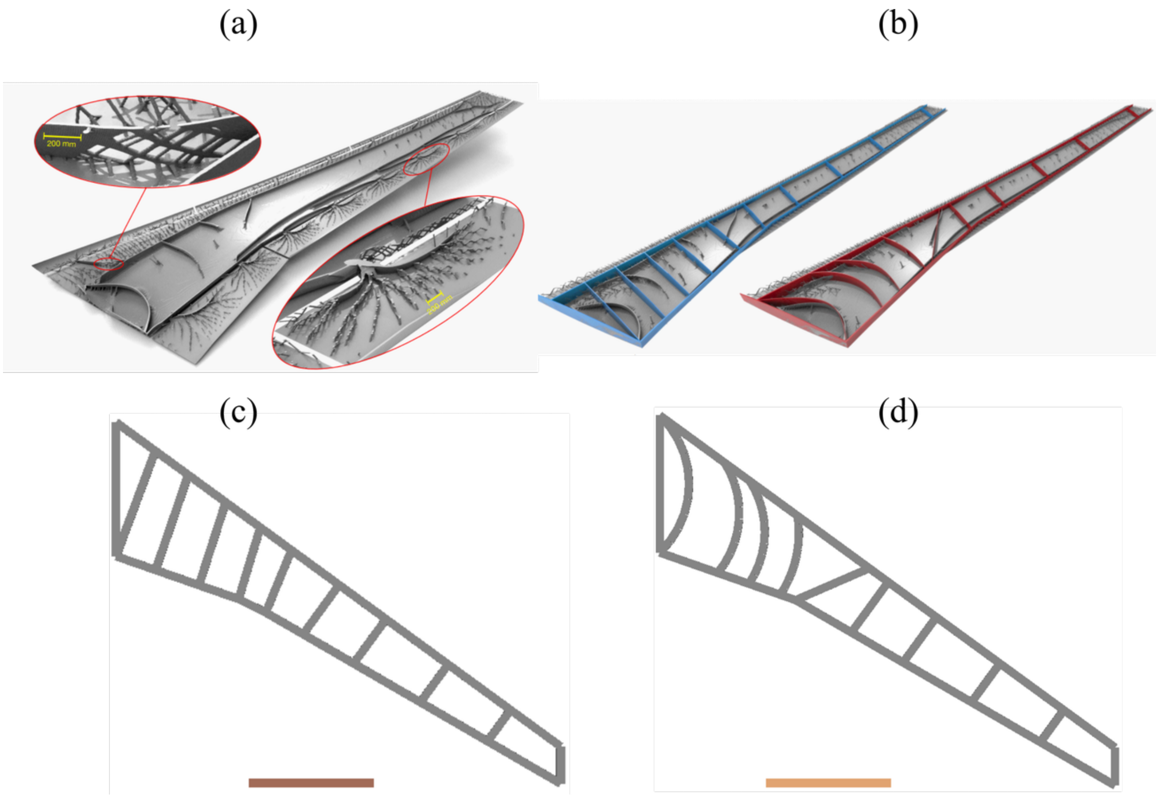

Figure S43: Aircraft wing design, (a) Giga-voxel morphogenesis optimized design, (b) conventional (BD) and curved rib (CD) designs, (c) and (d) 2D pattern of conventional and curved rib designs.[(a) and (b) are reprinted by permission from Nature [37], copyright 2017.

A.18 Basic design (BD) and curved rib design (CD)

For decades, the design of aircraft wing structures has been based on utilizing straight ribs (herein named Basic Design (BD)) to stiffen the wing. Recent advances in manufacturing enable the realization of wings with more complex internal architectures. From Giga-voxel-resolution computational optimization one another design with curved ribs has been obtained. These two designs have been chosen as a baseline for comparison (Figure S43).

A.19 Dragonfly inspired wing

Machine learning along with graphic statics is used to generate new designs for the core of the wing. Two design features have been implemented. First, the subdivision density is applied. This feature allows reducing details of the design (reducing the number of trusses) significantly, which reduce manufacturing complexity (especially for fabrication by assembling). Second, the minimum and maximum truss thickness are constrained to allow overcoming manufacturing limitations on small scales. Four different bio-inspired cellular structures have been designed to investigate the effect of the subdivision density, which reduces in V0, V1, V2, and V3 designs, respectively (Figure S44).

Complied with the minimum thickness of struts manufacturable by the adopted 3D printing technology (i.e. 0.3mm minimum thickness for fused deposition modeling (FDM)), we also modify those designs presented in Figure S45 based on the manufacturing constraints. Although these modified designs compromise the compatibility with the designs of actual dragonfly patterns, they reduce the manufacturing defects in the as-built samples compared to as-designed ones. For instance, the V0 design with the numerous number of beams with a limitation of $t/L = 0.0044$, has lost the main streams of the dragonfly pattern, which consists of thick elements, disappears.

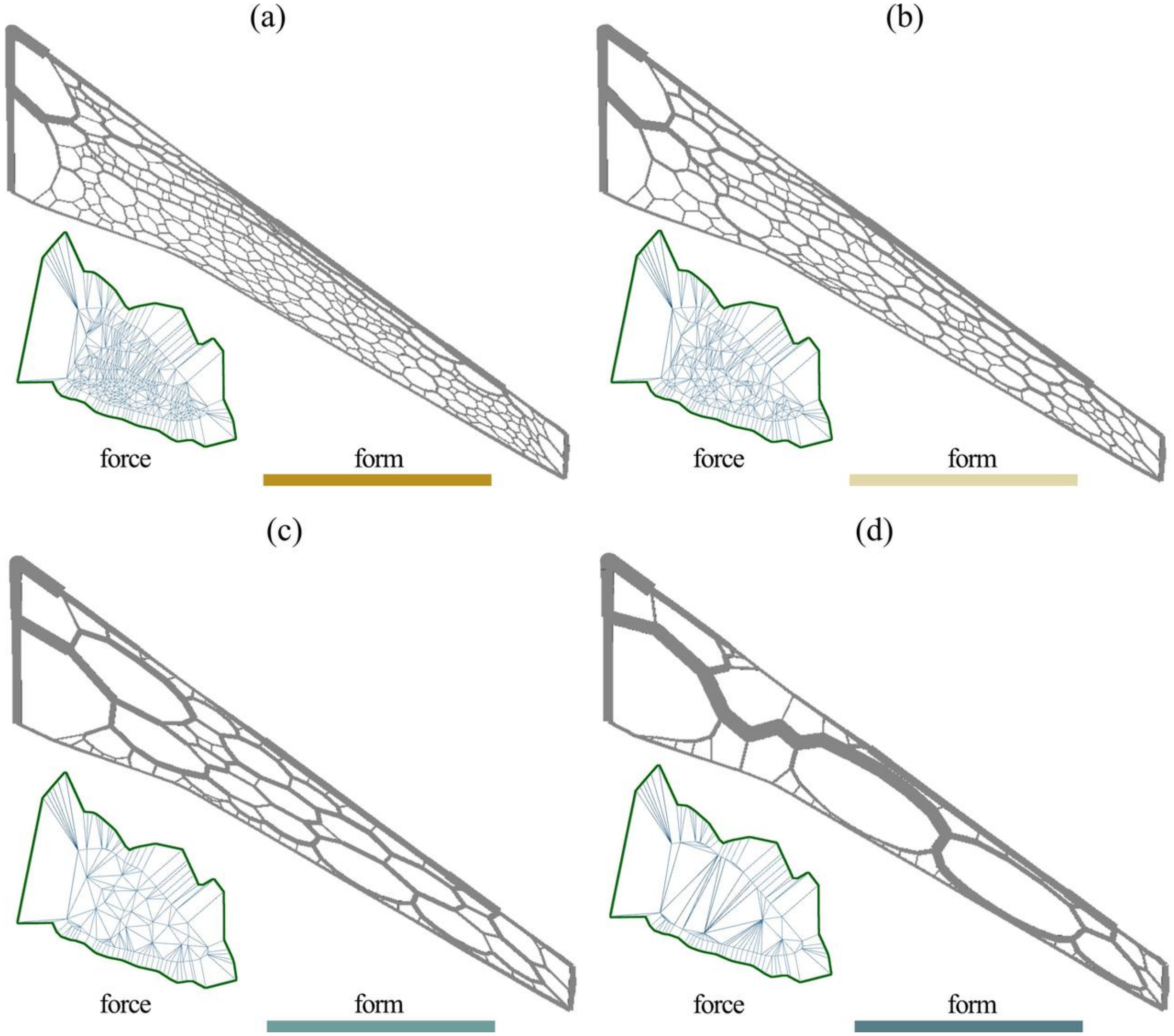

Figure S44: Aircraft wing design base on dragonfly wing, (a),(b),(c) and (d) are designs with subdivision density = 1.0, 0.5, 0.1, and 0.01, respectively.

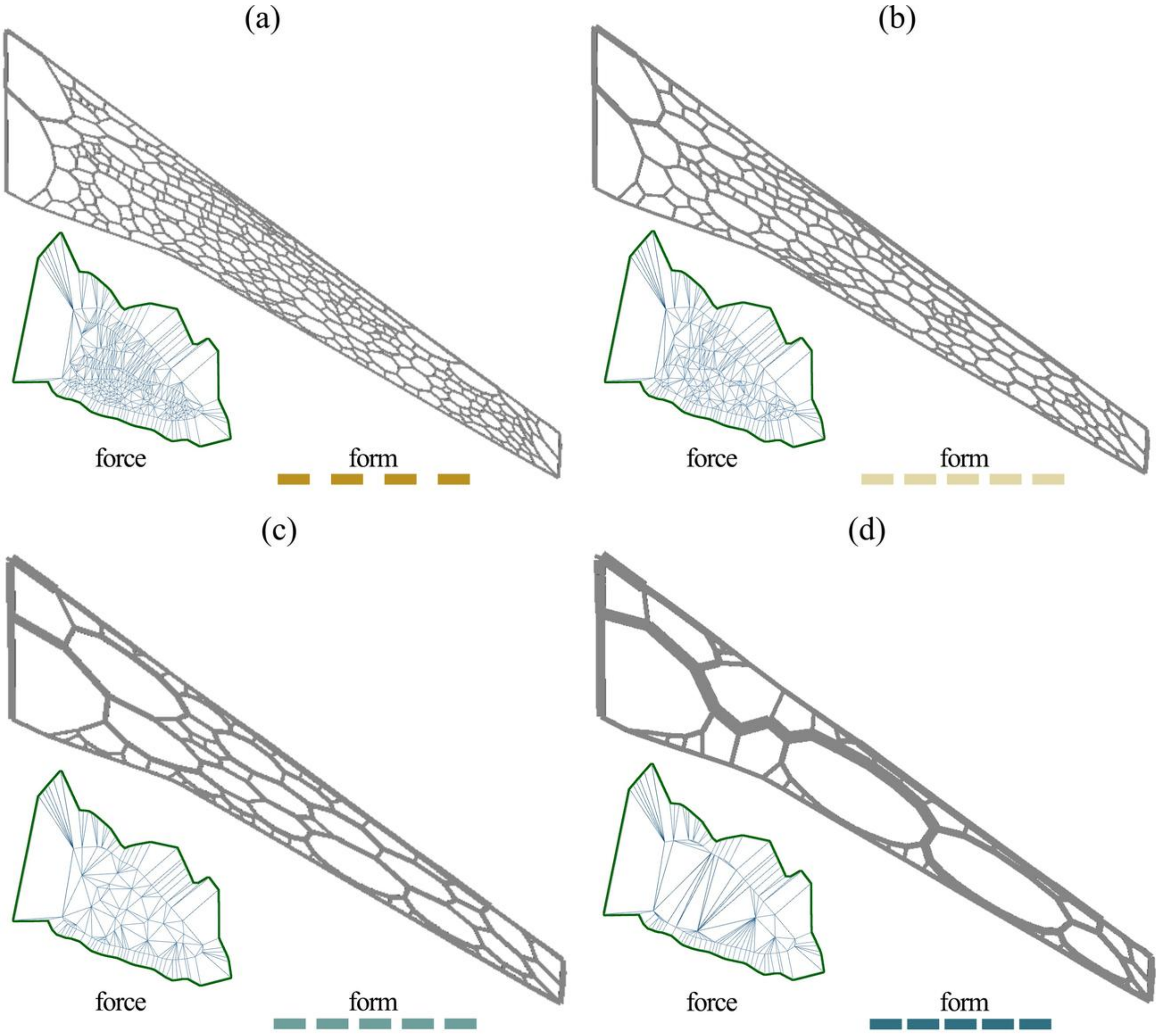

Figure S45: Aircraft wing design with thickness limitation of manufacturing process, (a), (b), (c), and (d) are designs with minimum and maximum structural thickness = V0 [0.3,0.6], V1 [0.3,0.8], V2 [0.3,0.94], and V3 [0.3,1.0], respectively.

A.20 Topology optimization

Topology optimization is a powerful tool for designing a structure with optimized property with respect to specific load conditions. Abaqus CAE 2019 optimization tool is used to obtain the optimized design for specific load cases. According to the fact that the wing needs a frame, the boundary domain has been considered constant and mid-part optimized for minimum compliance. We consider that the wing is clamped, where it is attached to the airplane body (Figure S46). To reduce complexity and obtain 2D extruded pattern we assumed a shell structure for the wing in the optimization task. In optimization, we assumed that the height of the wing in the z-direction is small (the value of $h/L = 0.033$ is considered to be sure results with shell elements are valid). Before optimization, the whole domain is discretized by 56000 triangular quadradic shell elements. The airplane wing is exposed to two main forces, which are lift and drag. In design, it is aimed to maximize lift force, while minimizing drag forces. Due to the asymmetric design of the wing frame, lift force generates torque over the wing. Hence, for simplicity three loading conditions, including out-of-plane, rotational, and in-plane deformation, have been considered at the tail of the wing. In all optimization tasks, the wing is clamped and loads are applied as point loads to the tail of the wing. To consider the effect of combined loading, a new load case for the combination of the abovementioned three load cases has been considered. It is important to mention topology optimization like other optimization methods does not guarantee to give global optimized design.

Optimization for four different load cases and three different total relative densities of 0.18, 0.28, and 0.38 has been conducted, and optimized structures have been shown in Figure S47. Here density for 2D structure is defined by a ratio of the area of the structure in XY plane to the total area of the wing.

A.21 Comparison of external load in graphic statics with actual pressure profile on the wing

Comparison of virtual load diagrams with actual pressure contour of airplane wings shows some similarity in terms of the load distributions. In both of them, the front edge experiences more pressure/load, and from left to right, the pressure/load decrease (Figure S48).

A.22 Finite element analysis (FEA) results

To conduct FEA simulations ABAQUS CAE 2019 is used. In FEA analysis we have considered three elastic stiffness, including, out-of-plane, torsional, and in-plane stiffness. Each stiffness has been calculated by applying point load at the tip and obtaining deformation at the tip, and by dividing two values. The wing must be very stiff for out-of-plane loads. The reason is that the lift-to-drag force ratio for wings is high and it is increasing with more optimized airfoil designs coming. This value is approaching 20 for Boeing 777 [38].

According to the results of the table S7, FEA analysis results have a good agreement with experimental results. Generally, results have a better agreement in bio-inspired designs with thickness limitations than samples with no thickness limitations. The average difference for bio-inspired designs with thickness limitation is 1.7%, 3.7%, and 6.5% for out-of-plane, torsional, and in-plane stiffness, respectively. The average difference for bio-inspired designs with no thickness limitation is 9.2%, 12.1%, and 6.5% for out-of-plane, torsional, and in-plane stiffness, respectively. There are two major reasons for the difference between numerical and experimental results. First, beam element has been utilized in numerical analysis, while few struts do not have a high slenderness ratio. Second, there are some inevitable 3D printing defects especially for designs with no thickness limitations. The higher difference in results of the designs with no thickness limitation indicates that printing accuracy can be a major source of difference.

Besides, the overall height of the wing is the smallest dimension, and make out-of-plane stiffness is much smaller than in-plane stiffness. After out-of-plane stiffness, torsional stiffness is more important. The reason applied lift forces on the asymmetric design of the wing (the great offset of the center of the area from the main axis of the wing) generates a great amount of torque. Dragonfly-inspired structures are made of numerous elements and in some cases, they can reach more than 10000 beams. Modeling such

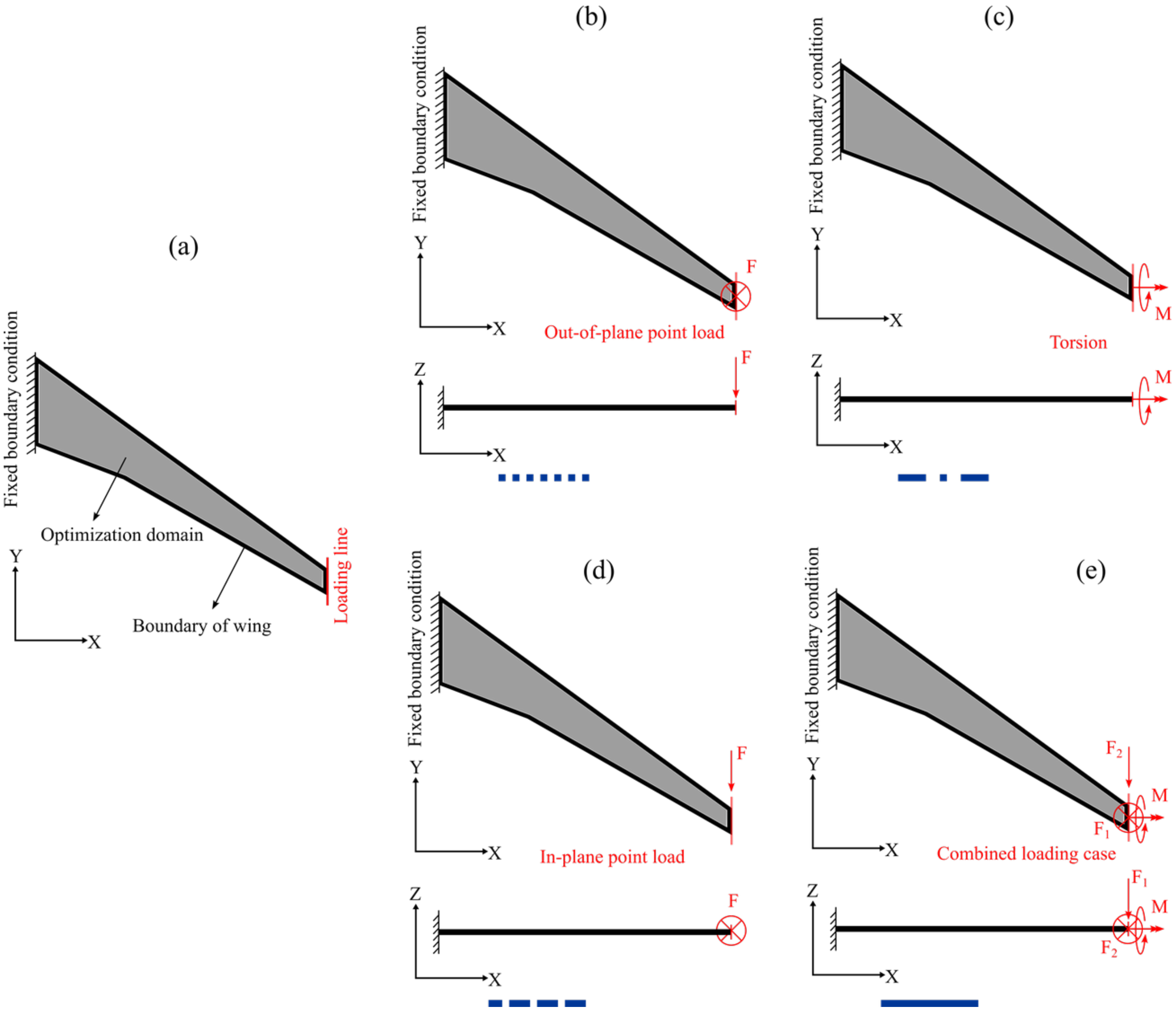

Figure S46: Designing the wing core by topological optimization, (a) defining boundary conditions and medium, which optimization is conducted (b) designing a wing for minimum compliance with respect to out-of-plane point load at the tip of the wing, (c) designing a wing for minimum compliance with respect to torsion of the wing tip (d) design a wing for minimum compliance with respect to in-plane point load at the tip of wing (e) design a wing for minimum compliance with respect to the combination of the loads presented in (b), (c), and (d).

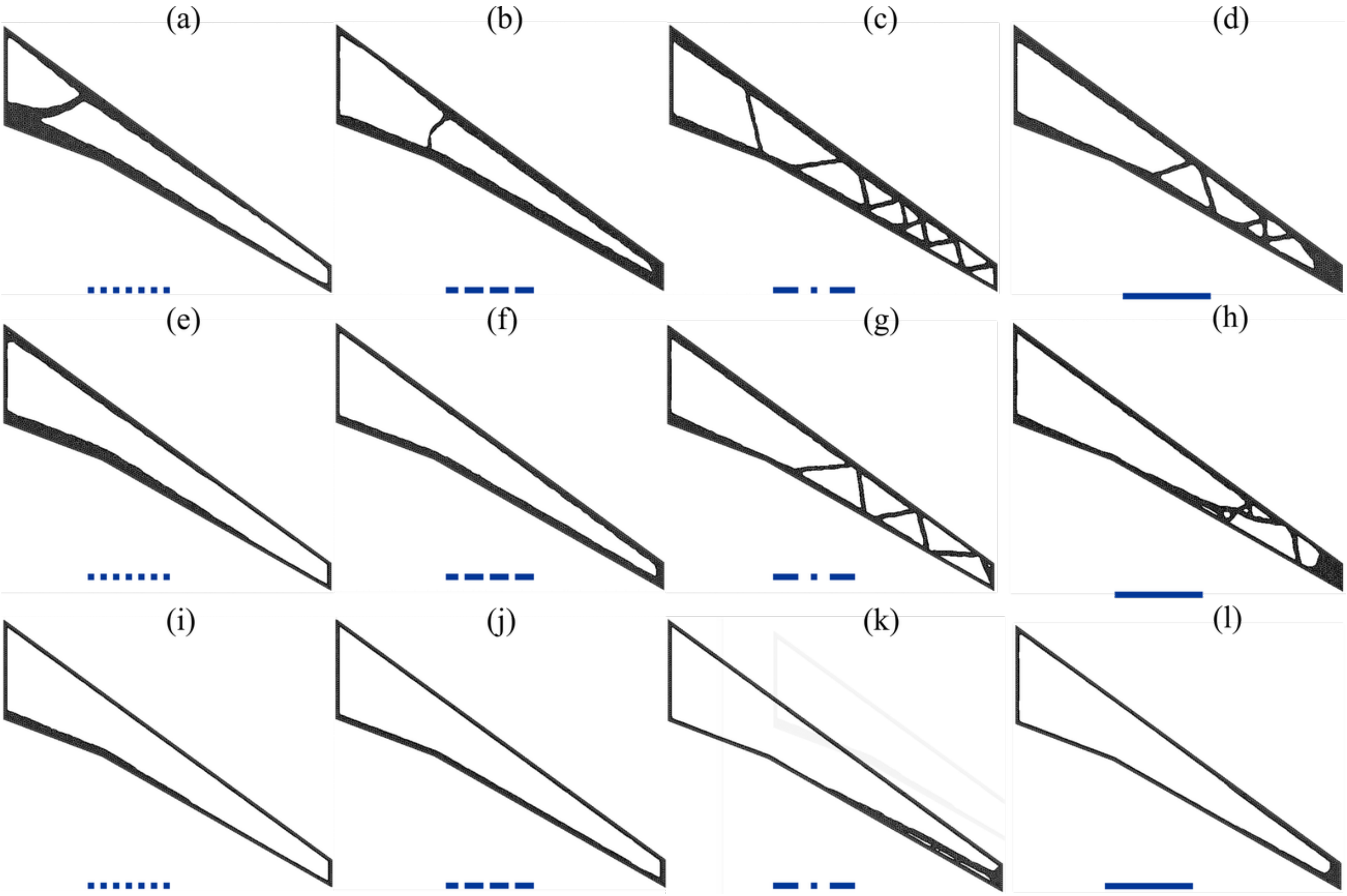

Figure S47: Aircraft wing design with topological optimization, (a), (e), and (i) design a wing for minimum compliance with respect to out-of-plane point load at the tip of the wing with 0.38, 0.28, and 0.18 relative density, respectively, (b), (f), and (j) design a wing for minimum compliance with respect to torsion of the wing tip with 0.38, 0.28, and 0.18 relative density, respectively, (c), (g), and (k)design a wing for minimum compliance with respect to in-plane point load at the tip of wing with 0.38, 0.28, and 0.18 relative density, respectively, (d), (h), and (l) design a wing for minimum compliance with respect to the combination of three load cases with 0.38, 0.28, and 0.18 relative density, respectively.

Out-of-plane stiffness (normalized to the basic design)									
	CD	V_0	V_1	V_2	V_3	V_0^L	V_1^L	V_2^L	V_3^L
FEA	1.01	1.06	1.08	1.17	1.15	0.9	1.05	1.25	1.28
Experiment	0.975	1.05	1.01	1.02	1.01	0.905	1.055	1.245	1.215
Diff (%)	3.6	1	7	14.7	13.9	0.6	0.5	0.4	5.3
Torsional stiffness (normalized to the basic design)									
	CD	V_0	V_1	V_2	V_3	V_0^L	V_1^L	V_2^L	V_3^L
FEA	1.04	0.75	0.76	0.91	0.92	0.78	0.94	1.06	1.1
Experiment	0.95	0.85	0.88	1.01	1.06	0.8	0.88	1.05	1.155
Diff (%)	9.5	11.8	13.6	9.9	13.2	2.5	6.8	1	4.8
In-plane stiffness (normalized to the basic design)									
	CD	V_0	V_1	V_2	V_3	V_0^L	V_1^L	V_2^L	V_3^L
FEA	0.92	0.7	0.7	0.7	0.6	0.7	0.82	0.88	0.9
Experiment	0.855	0.71	0.69	0.73	0.74	0.71	0.74	0.92	0.82
Diff (%)	7.6	1.4	1.5	4.1	18.9	1.4	10.8	4.3	9.7

Table S7: Normalized results of numerical analysis and experimental results and their difference for all bio-inspired and curved rib designs.

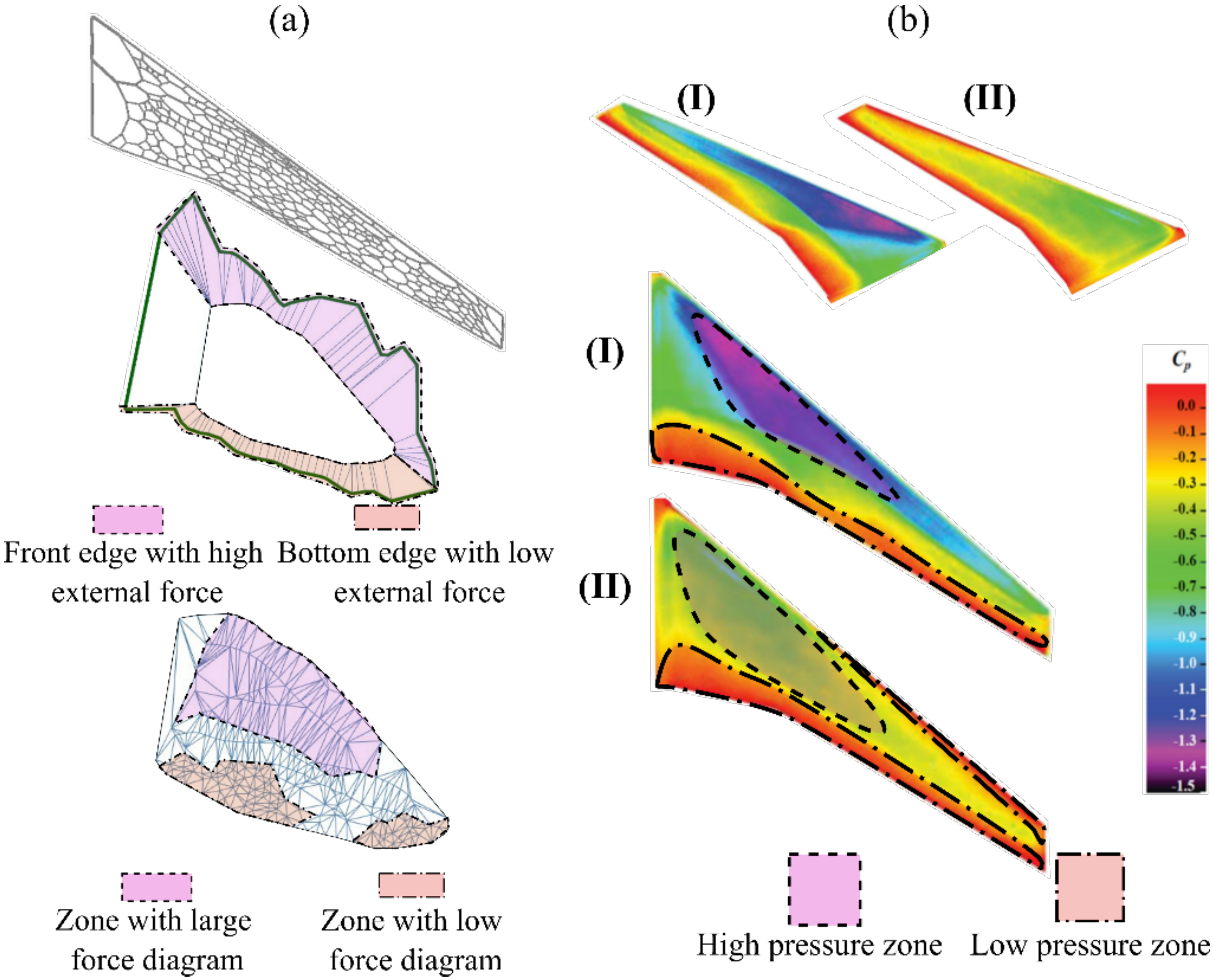

Figure S48: Comparison of (a) the external load diagram and (b) the actual pressure contour on Boeing 777 wing for two different load cases (Cp is the pressure coefficient) [37].

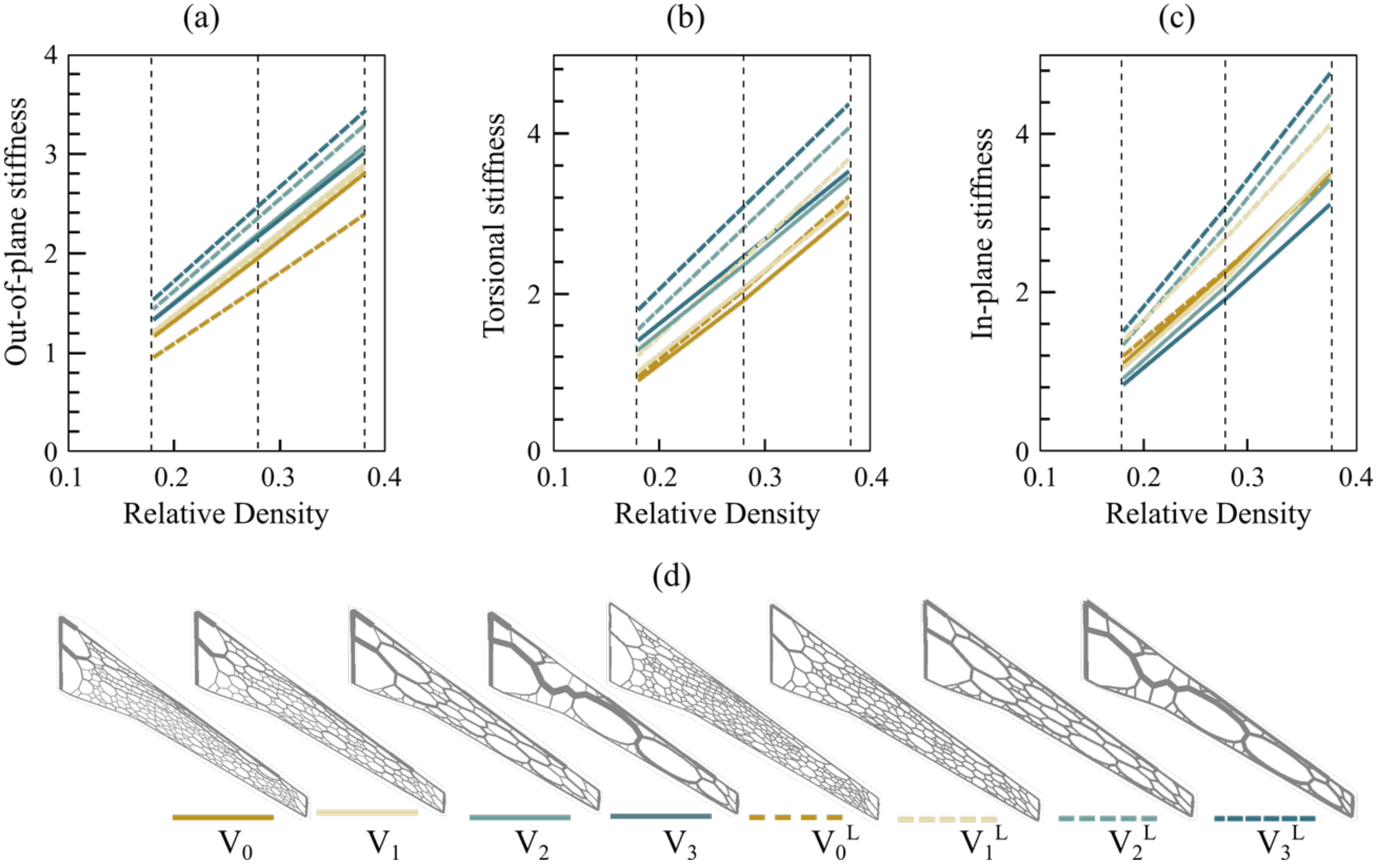

Figure S49: Finite element analysis for (a) out-of-plane, (b) torsional, and (c) in-plane stiffness of dragonfly-inspired designs with thickness limitation and conventional (basic) and curved rib designs with 0.18, 0.28, and 0.38 total relative densities. Results are non-dimensionalized by dividing them by the corresponding results of V0L with 0.18 density.

a complex geometry with solid elements with a proper element aspect ratio is computationally expensive. Hence, all dragonfly-inspired structures and basic and curved rib designs are simulated with beam elements. To compare these results with the topologically optimized design, the height to length ratio of all designs is considered $h/L = 0.033$. All results are non-dimensionalized by diving with the corresponding results of dragonfly design V0L with a total relative density of 0.18.

Numerical results in Figure S49 show more variations in terms of stiffness for designs with minimum acceptable thickness considerations compared to those designs without manufacturing limitation considerations. Results suggest that stiffness (with respect to the point load) of dragonfly-inspired design inherits its performance from the pattern of main streams (thicker beams) and this stream can be preserved even with a reduced number of features. Among all loading cases on designs with thickness limitation, V3L shows better performance; by increasing the number of features (struts) from V3L to V0L, the main streams of the pattern vanish and all out-of-plane, in-plane, and torsional stiffness reduces.

By comparing the results of basic and curved rib designs with those obtained for dragonfly-inspired designs with thickness limitation considerations, it is found that the dragonfly-inspired designs may offer higher out-of-plane and torsional stiffnesses, while they are commonly softer in terms of in-plane stiffness. Considering the out-of-plane and torsional stiffnesses as the main design criteria of the Aeroplan wings, we can observe that V3L dragonfly-inspired design shows considerable improvement compared to the basic design as well as the previously proposed curved rib design.

By evaluating optimized structures (Figure S51), it is obvious each optimized design has superior perfor-

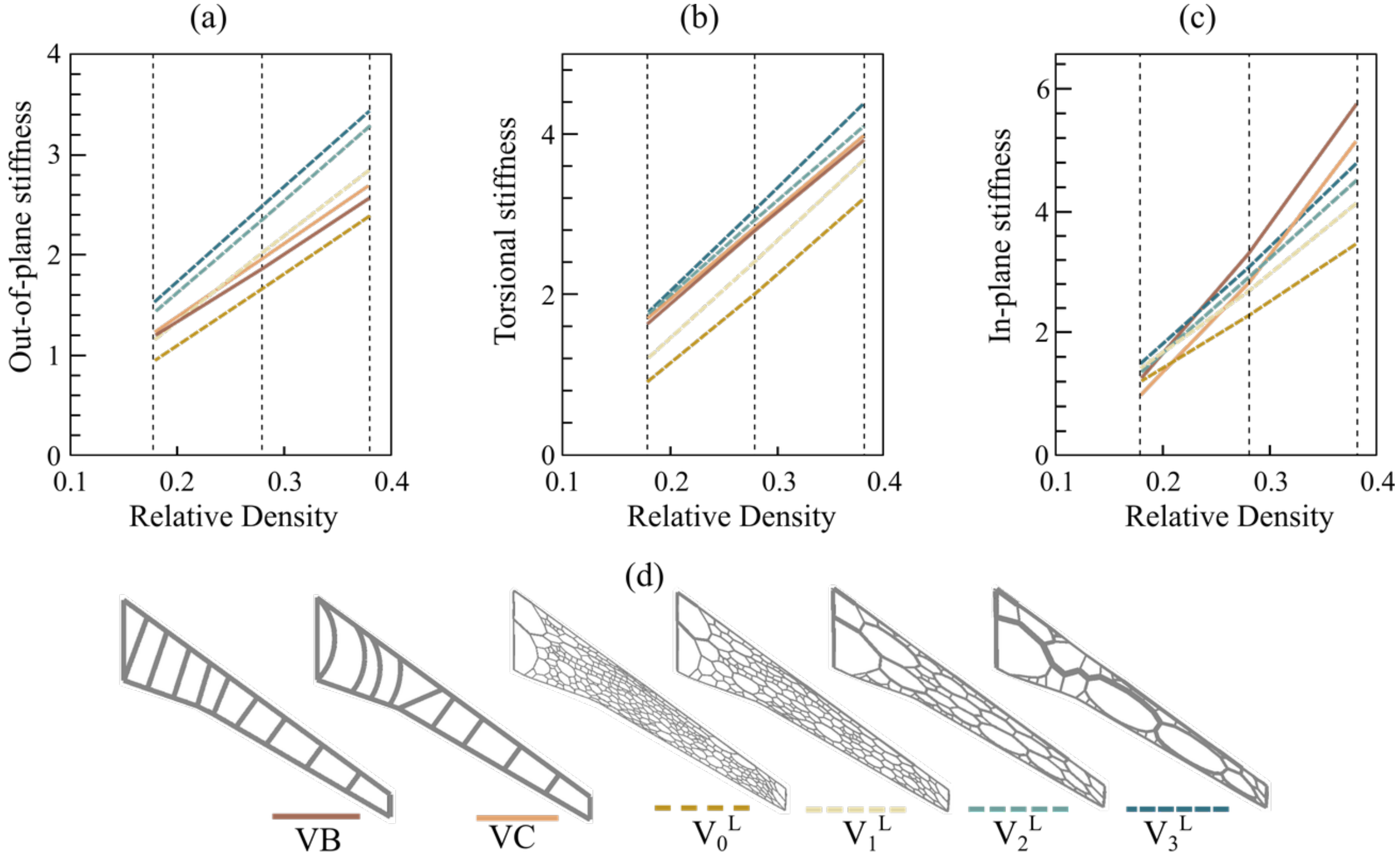

Figure S50: Finite element analysis for (a) out-of-plane, (b) torsional, and (c) in-plane stiffness of dragonfly-inspired designs with thickness limitation and conventional (basic) and curved rib designs with 0.18, 0.28, and 0.38 total relative densities. Results are non-dimensionalized by dividing them by the corresponding results of V0L with 0.18 density.

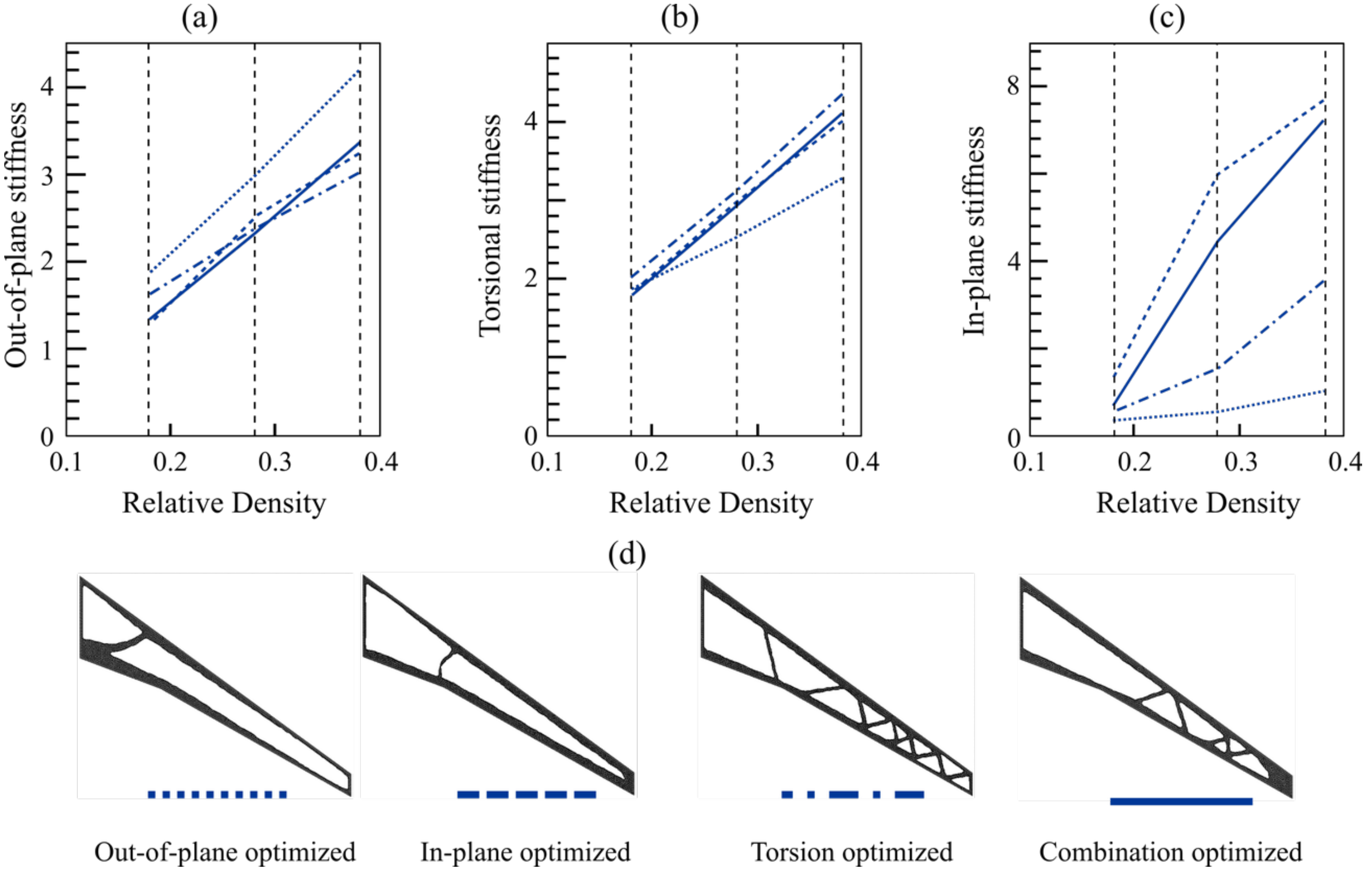

Figure S51: Finite element analysis for (a) out-of-plane, (b) torsional, and (c) in-plane stiffness of topologically optimized designs with 0.18, 0.28, and 0.38 total relative densities. Results are non-dimensionalized by dividing them by the corresponding results of V0L with 0.18 density.

mance with respect to the load case (objective function) that is optimized for. For instance, the out-of-plane optimized structure performs very well with respect to the out-of-plane point load, however, it has very inferior performance with respect to the other load cases. Numerical results show that the difference between the performance of the alternative optimized designs vanishes for lower densities. The reason is that by decreasing total relative density, the boundary is getting more ratio of the area (since the boundary is constant) and less area is remained to be optimized. By comparing the optimization results, we recognize that the optimized design for the combined loading case (solid lines in Figure S51) is the second stiffest among all the optimized structures Hence, it might be considered a better overall design among all topologically optimized designs, specifically for higher relative densities. It is important to mention that aircraft wings are subjected to distributed air pressure in service, while the presented topology optimized designs are obtained for point loads at the tip leading to a highly-porous hollow-like cellular core for the wing which may not be sufficient for supporting the deformation of the solid sheet panels forming the sandwich structure of the wing. However, these optimized designs can be utilized to provide a measure for performance comparison with the dragonfly-inspired designs under the same loading conditions as presented in Figure S52.

In Figure S53, the best performance of dragonfly design, which is obtained by V3L under point load is compared with basic, curved rib and overall best-optimized design (optimized with respect to the combination of loads). Results show the dragonfly-inspired design V3L overperforms other designs in out-of-plane and torsional stiffness, while it is softer under the in-plane load case. According to the importance of out-of-plane and torsional stiffness, it can be concluded dragon fly design V3L is a better design. These findings are valuable because it shows that topology optimization does not necessarily provide globally

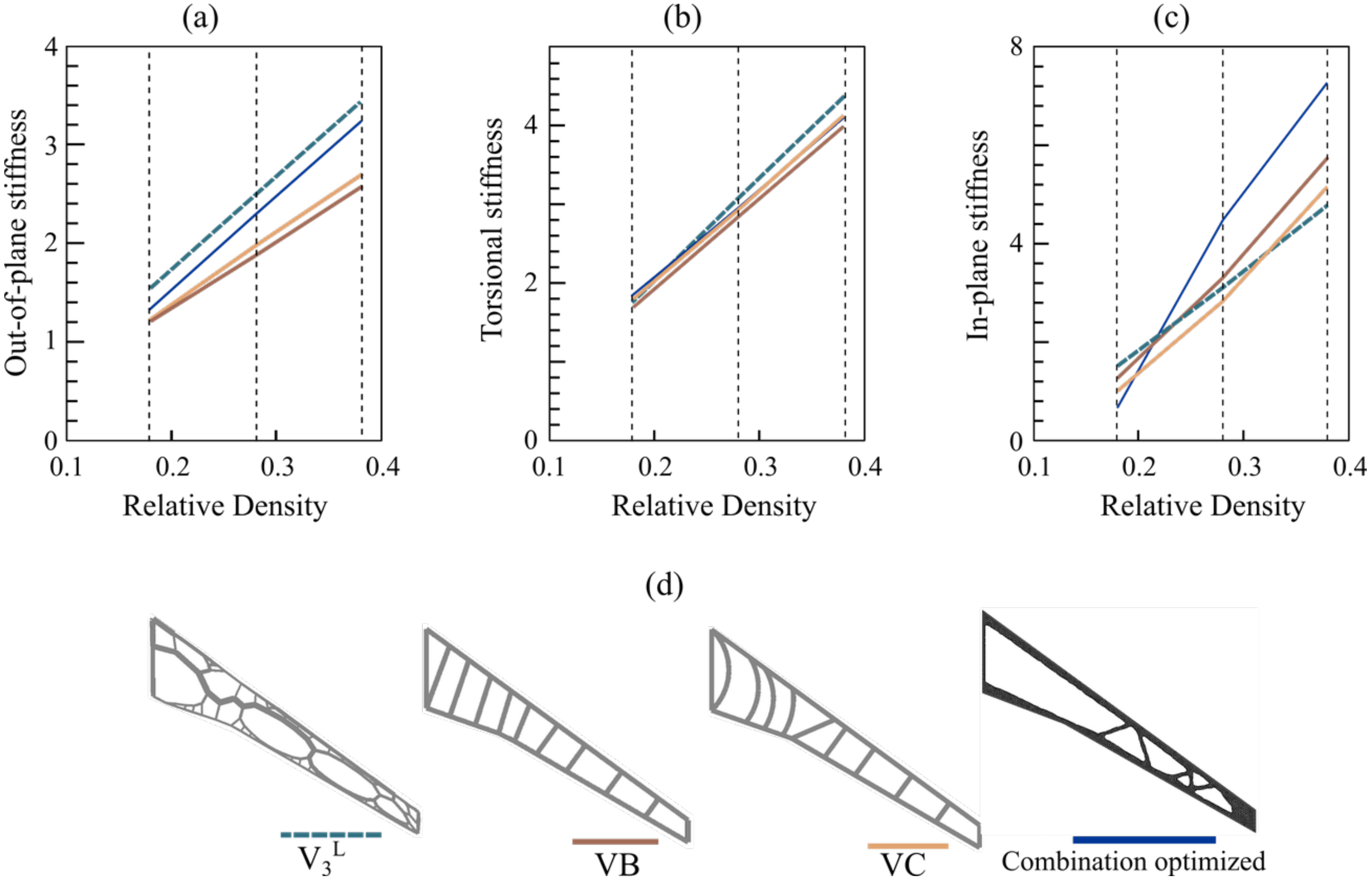

Figure S52: Finite element analysis of (a) out-of-plane, (b) torsional, and (c) in-plane stiffness of dragonfly-inspired design (V3L), conventional design, curved rib design, and topologically optimized design for the combined loading case for 0.18, 0.28, and 0.38 total relative densities. Results are non-dimensionalized by dividing them by the corresponding results of V0L with 0.18 density.

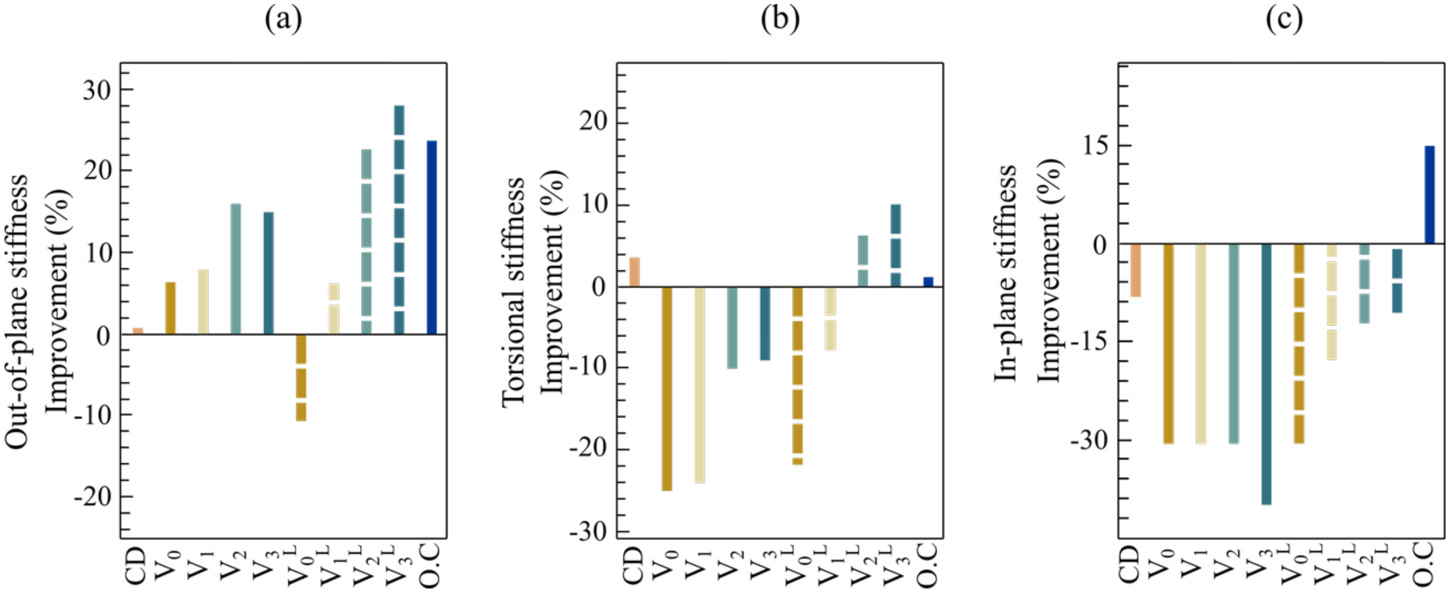

Figure S53: Finite element analysis of (a) out-of-plane, (b) torsional, and (c) in-plane stiffness of dragonfly-inspired designs, curved rib design, and topologically optimized design under the combined loading case (O.C); results are compared to the conventional design.

optimized design. In addition, the dragonfly-inspired design is not still optimized with respect to these load cases, which is suggesting that this design can be optimized further.

Improvement of each design with respect to the basic design is demonstrated in Figure S53. Results show that the curved rib design has a slightly better performance compared to the basic design under the out-of-plane and torsional loading cases. It is also obvious that implementing manufacturing constraints on the thinnest struts in the design causes a great variation in the performance of dragonfly-inspired designs. It is found that the performance of V3L is superior to other designs with a total relative density of 0.38, except for the in-plane stiffness.

The natural frequency of cellular wings can be crucial for evaluating their behavior under dynamic loads which is a common load in aircraft in service, in which air pressure can fluctuate by wind or through the interaction of the fluid and structure. Besides, for designing small-scale robotic drones, which fly by flapping their wings, the speed of flapping is constrained by the natural frequency of the wing structure. It is important to make wings with high natural frequencies that are not unstable under dynamic loads. Here six modes of natural frequencies have been obtained and compared to the basic design. The first mode is about out-of-plane deformation. The second, fourth, and fifth modes are the first, second, and third modes of buckling, respectively. The third and sixth modes are rotation at the tip and double rotation on the tip and middle of the structure, respectively. The importance of natural frequencies reduces sequentially from one to six. Results in Figure S54, interestingly show the best design is the dragonfly design without any thickness limitation, which replicates the insect wing more accurately. The second point is, that there is an optimum number of features. It shows V2 has the best performance, which indicates better mass distribution in addition to having higher stiffness for more crucial modes (mode1 and mode3).

A.23 Material characterization

For the characterization of the solid material property, five dogbone samples have been 3D printed and tested based on the ASTM-D628 standard. According to the experimental results shown in Figure S55, the PLA samples printed by fused deposition modeling (FDM) possess the Young modulus of 1.26 ± 1% GPa.

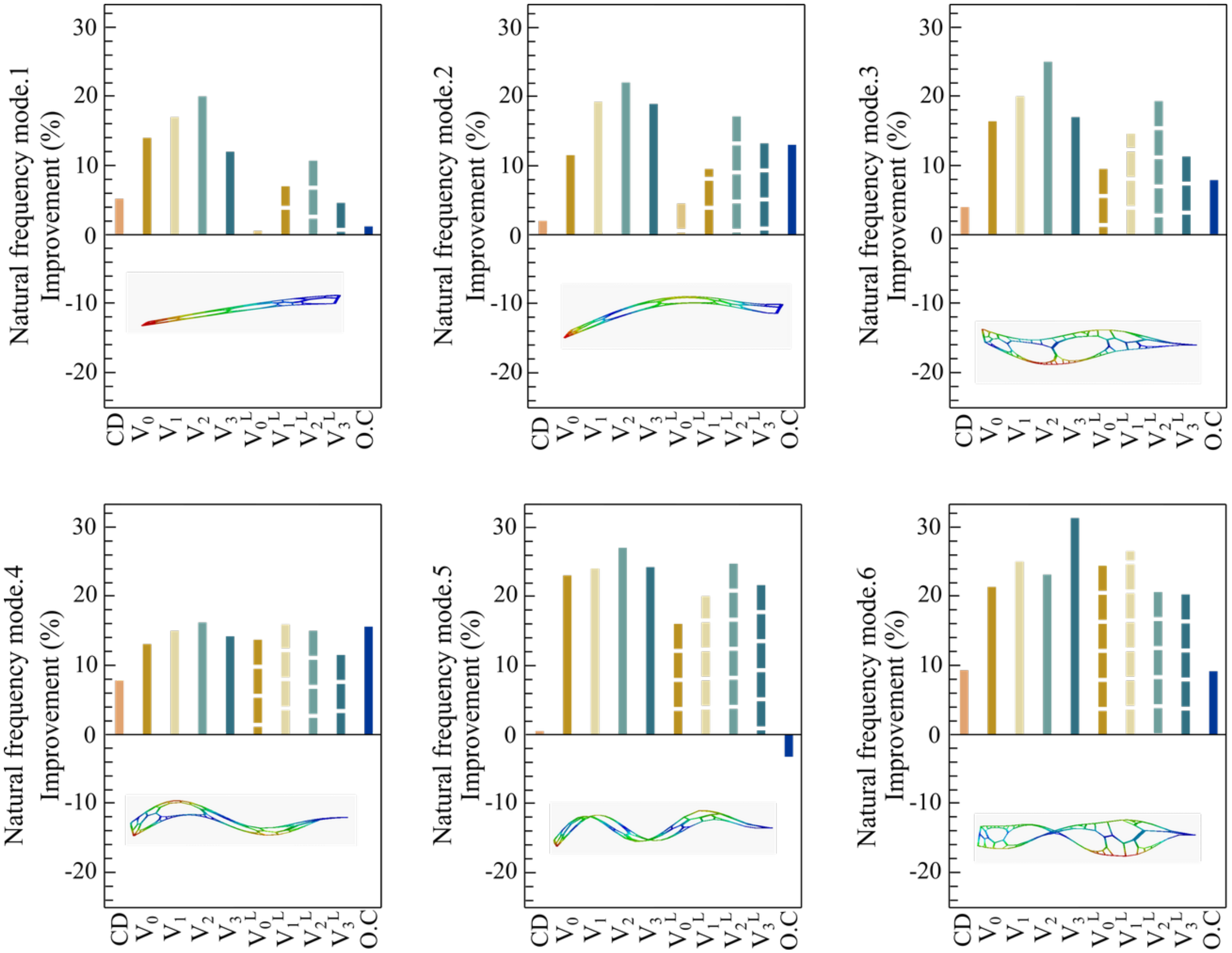

Figure S54: Finite element modal analysis of dragonfly-inspired designs, curved rib design, and optimized design (with respect to the combination of load cases); results are compared to the conventional design.

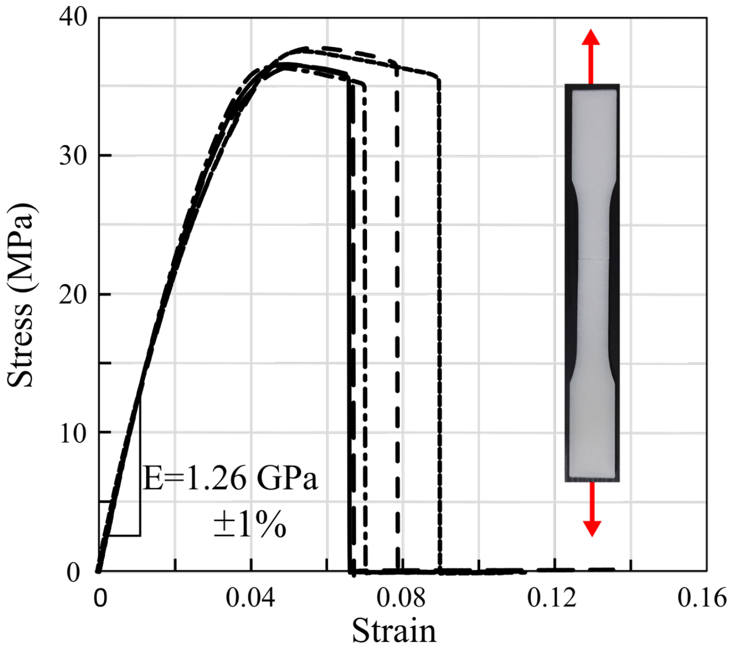

Figure S55: 3D printed dogbone sample made out of PLA under tension; five replicates have been used in the test.

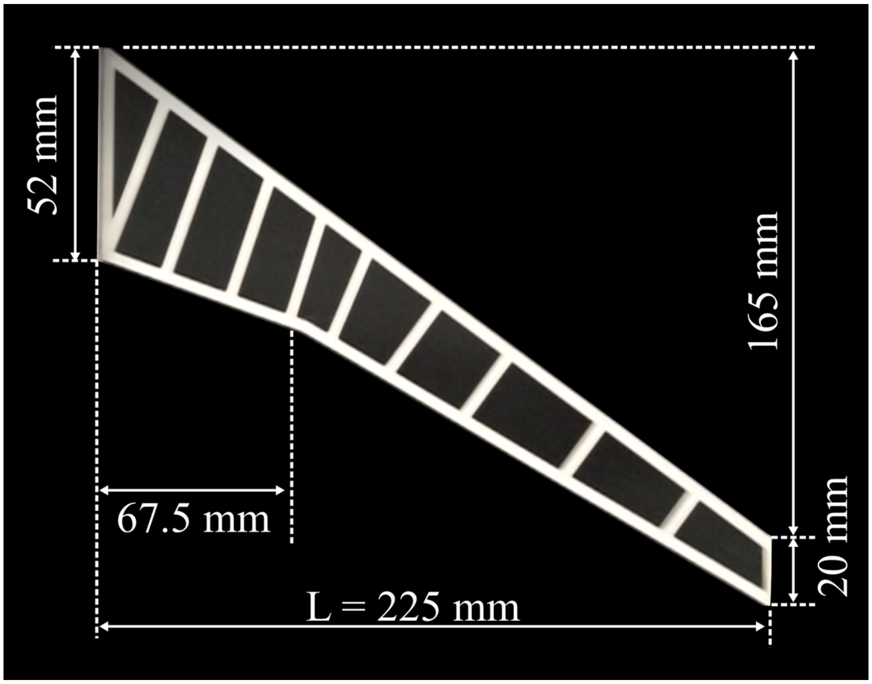

Figure S56: Dimensions of the 3D printed cellular wing boundary.

A.24 Sample dimensions

All 3D-printed samples have the same dimension in the XY plane. 3D Printed samples, which are tested under point load, have an out-of-plane thickness (or height) of h = 7.8 mm, while the samples, for the wind tunnel test, have h = 2.32 and h = 3.85 mm.

A.25 Experimental results for out-of-plane stiffness under point load

To obtain the out-of-plane stiffness (K(I)) under the point load, one side of the dragonfly-inspired wing is clamped while a compressive point load is applied on the tip of the wing using a 4mm round tip load module. Experimental results present saw-like force-displacement curves (Figure S57), which is mainly due to the sliding of the 3D printed samples with respect to the loading nose during the out-of-plane deformation. For each of the 3D printed designs (10 alternative designs), we have three replicates; we have also repeated the tests for each sample four times to further assure the accuracy and repeatability of the experimental data. We obtain three slop from each test and the average of them is reported as the out-of-plane stiffness under the point load.

A.26 Torsional stiffness under point load

Torsional stiffness (K(II)) has been determined by clamping the 3D-printed wings and applying torque on the tail. Three replicates for each design have been used and the tests have been repeated four times.

A.27 In-plane stiffness under point load

To determine the in-plane stiffness (K(III)) of 3D printed wings, the 4mm tip load module used in the out-of-plane point load test is also utilized. Each design has been 3D printed three times and the tests are conducted on each sample four times to assure the repeatability of the experimental test results.

A.28 Out-of-plane deformation

For obtaining the failure strength of the 3D-printed wings, both ends are clamped. Experimental results in Figure S60 demonstrate a brittle fracture behavior in all samples, mainly due to the brittleness of the

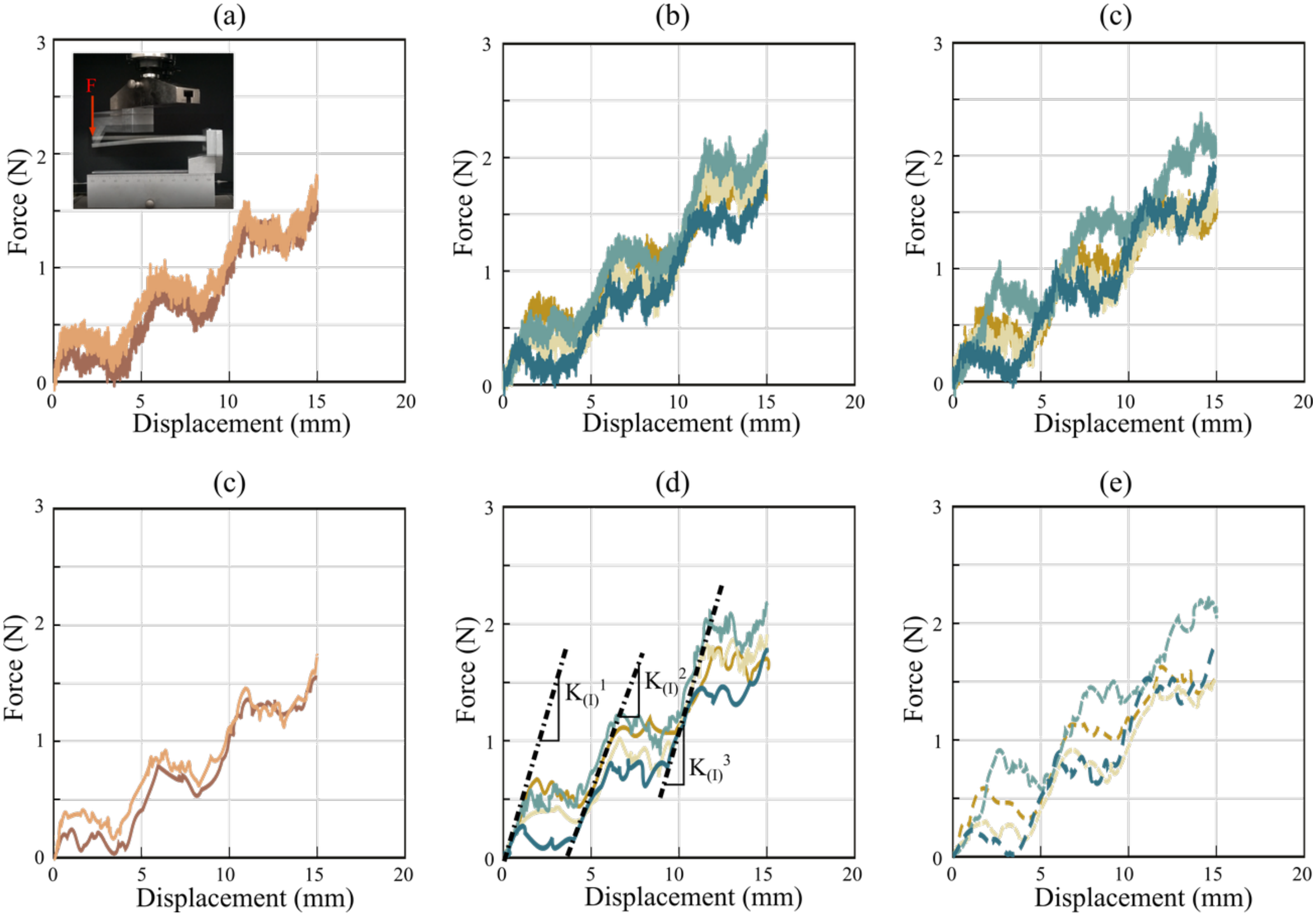

Figure S57: Experimental results of the out-of-plane loading at the tip of 3D printed wings: (a, actual) and (c, smoothed) conventional and curved rib designs, (b, actual) and (d, smoothed) dragonfly-inspired designs, and (c, actual) and (e, smoothed) dragonfly-inspired designs with thickness limitation considerations.

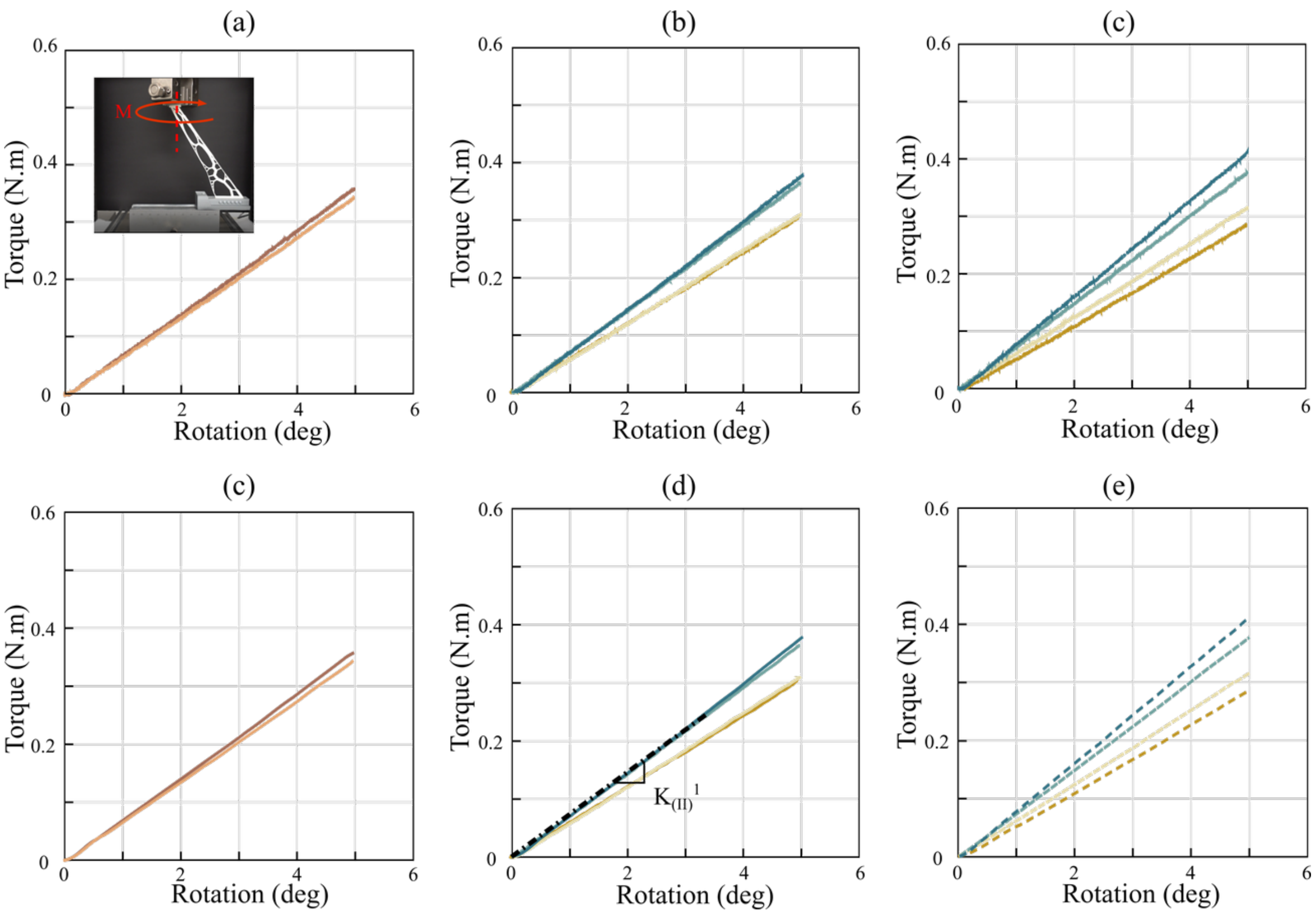

Figure S58: Experimental results for the torsion at the tip of 3D printed wings: (a, actual) and (c, smoothed) conventional and curved rib designs, (b, actual) and (d, smoothed) dragonfly-inspired designs, and (c, actual) and (e, smoothed) dragonfly-inspired designs with thickness limitation considerations.

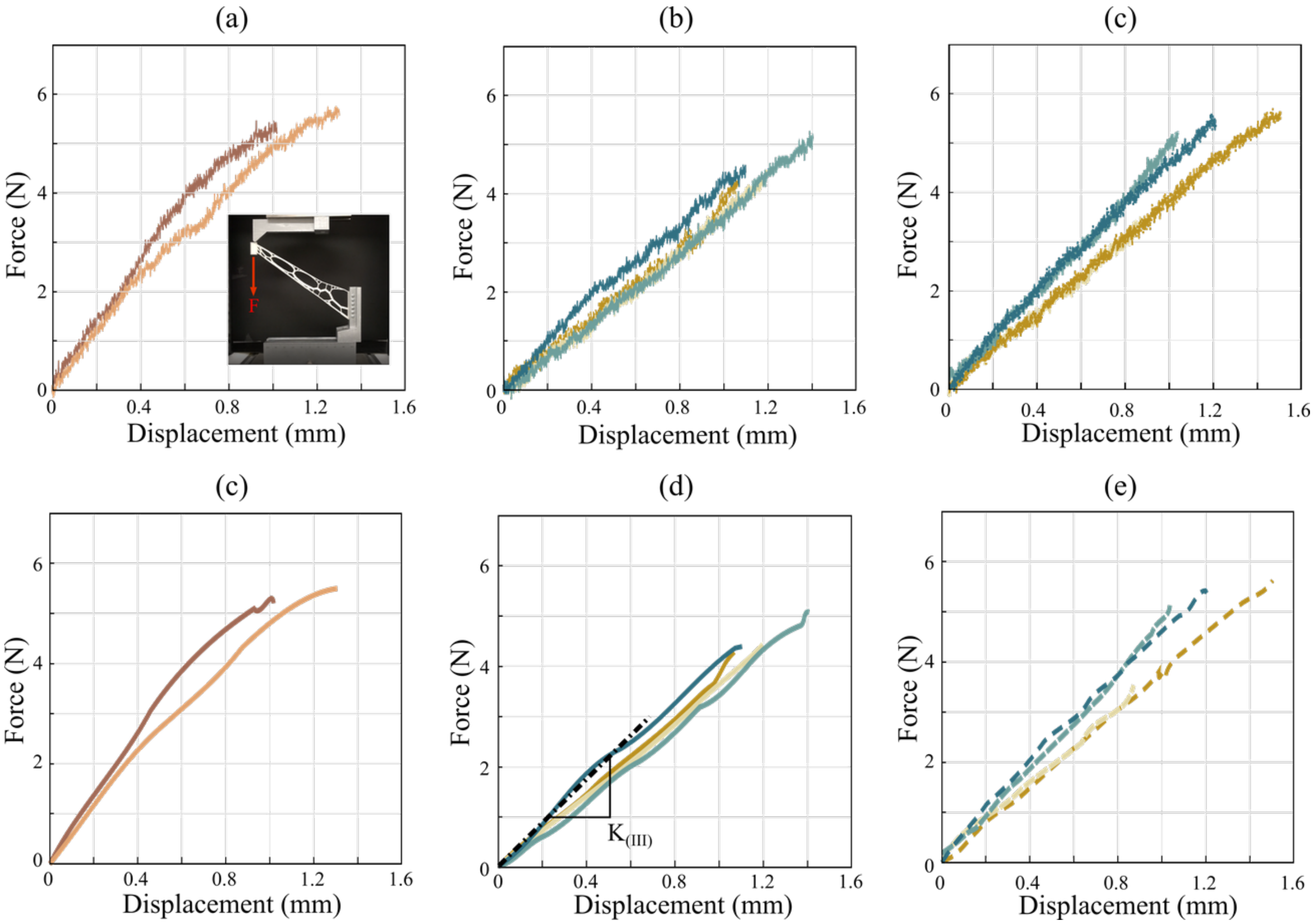

Figure S59: Experimental results of in-plane loading at the tip of 3D printed wings: (a, actual) and (c, smoothed) conventional and curved rib designs, (b, actual) and (d, smoothed) dragonfly-inspired designs, and (c, actual) and (e, smoothed) dragonfly-inspired designs with thickness limitation considerations.

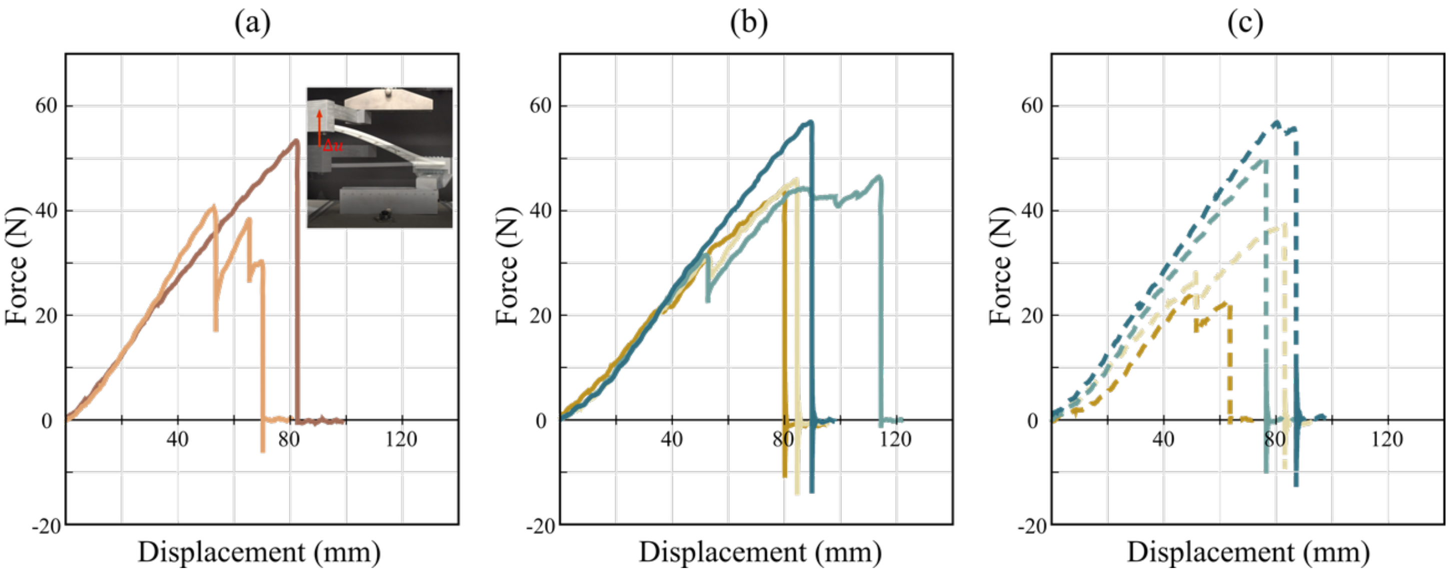

Figure S60: Experimental results till ultimate fracture of 3D printed wings experiencing an out-of-plane loading at the tip: (a) conventional and curved rib designs, (b) dragonfly-inspired designs, and (c) dragonfly-inspired designs with thickness limitation considerations.

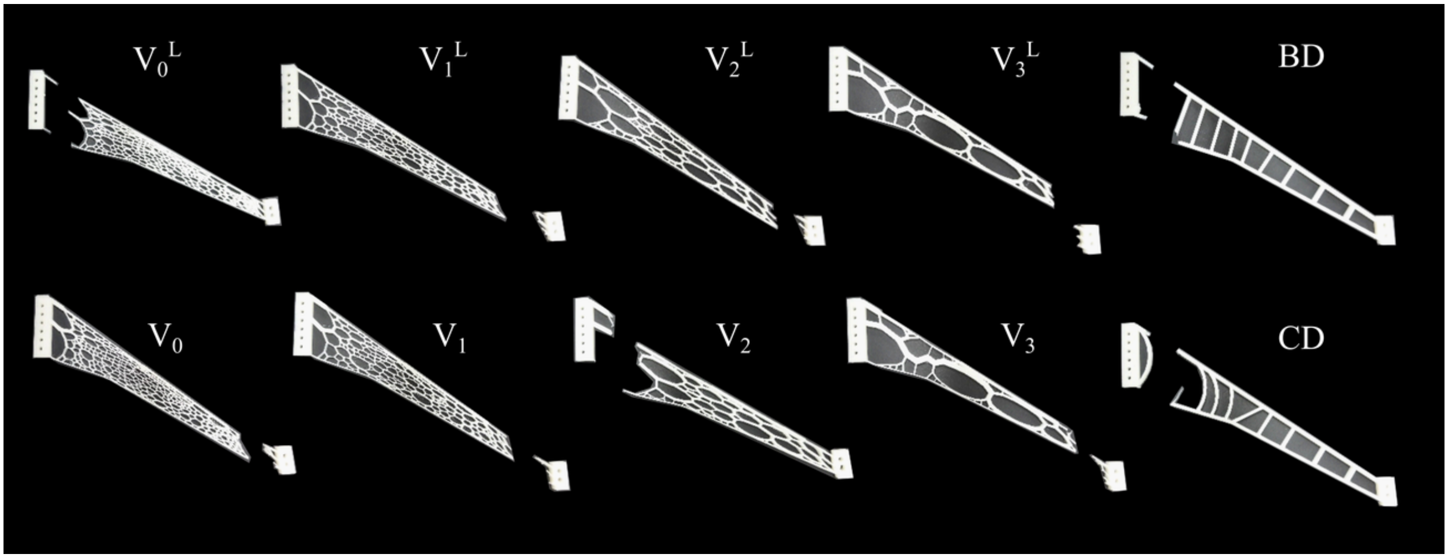

Figure S61: 3D printed wings after failure under the out-of-plane loading.

polylactic acid (PLA) used as a base material for the 3D printing of the samples.

According to the type of loading used for the failure analysis (Figure S60), shear force and moment at the tip of the wings are relatively high. We have also presented the samples after failure in Figure S61. In the majority of the dragonfly-inspired designs (except in V0L and V2 samples), failure occurs at the tip of the wing, which is desirable as wings do not experience shear and moment at the tip-point in service. In the opposite, basic and curved rib designs experience failure from a boundary that resembles attachment to the fuselage of an airplane in real-life applications.

A.29 Torsion

To obtain failure under torsional load, rotation continued until the sample breaks. Results are demonstrated until 50 degrees of rotation. In some samples after the initial break in one element, the other element remained to bear the load and could rotate over itself, which is not a reflection of torque over the whole structure. Hence, results are cut by 50 degrees to emphasize initial failure.

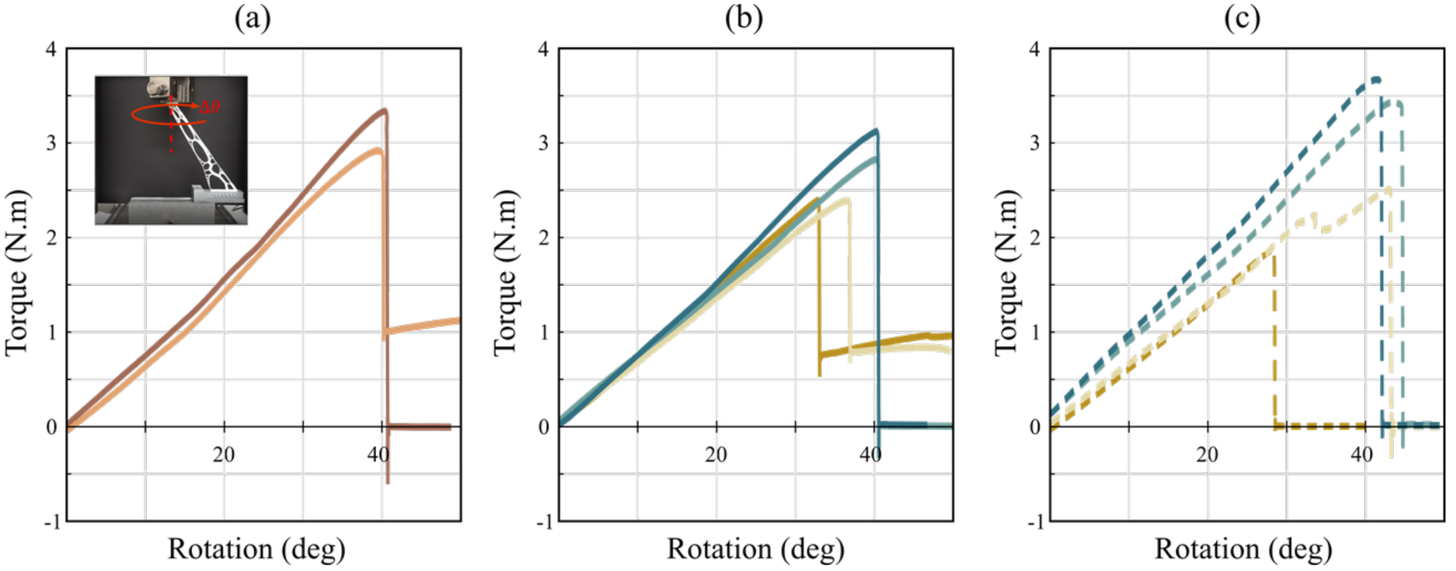

Figure S62: Experimental results till the first or the total failure of 3D printed wings under torsion: (a) conventional and curved rib designs, (b) dragonfly-inspired designs, and (c) dragonfly-inspired designs with thickness limitation considerations.

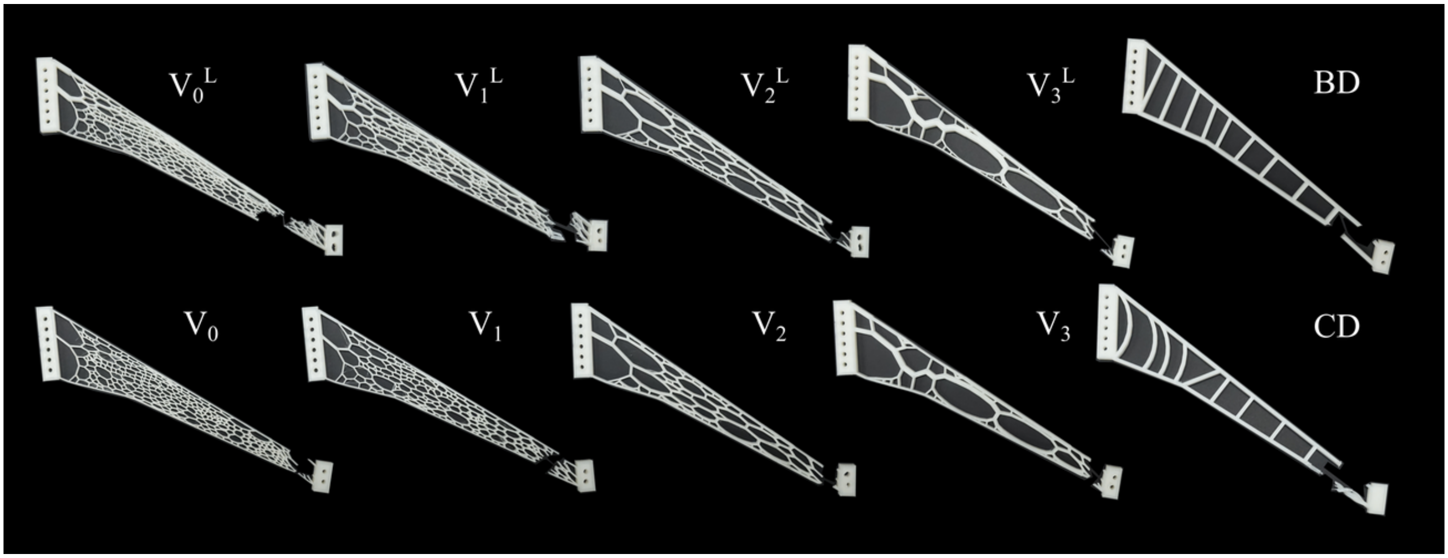

Figure S63: 3D printed wings after failure under torsion.

All samples under torsional loading break from the tip, which is expected as a smaller distance between beams at the tip cause to bear lower torque. In curved rib design, it is obvious first upper beam failed and the lower beam was rotating for much more and failed later.

A.30 3D printing quality and defects

FDM technology has been used in this study for 3D printing. Samples have been printed based on ±45°lay-up in a layer-by-layer fashion with a layer height of 0.3mm (Figure S64); a 0.4 mm nozzle has been used for 3D printing, leading to the accuracy limitation of 0.4mm in the XY plane. As expected, the quality of the as-built samples with thickness limitation consideration is much better than those printed without the manufacturing constraint considerations. For instance, for the V0 design, many struts have a thickness of 0.2-0.3 mm, which are printed only in a wavy shape or not printed at all. These 3D printing defects can reduce the performance of as-designed wings, indicating that 3D printing technologies with higher accuracy should be considered for manufacturing wings with finer geometrical features. However, the presented design methodology in this study can still offer alternative designs that outperform conventional

designs and are manufacturable with low-cost 3D printing technologies like FDM if the minimum thickness limitation is considered during the design process.

A.31 Wind tunnel test

In a real-life application, wings should also perform well under the lift force of air pressure. As a result, we have also studied the out-of-place stiffness of the 3D printed composite wings (composed of the designed cellular core sandwiched between two solid thin plexiglass sheets) under wind pressure; samples are tested perpendicular to the wind direction. To transform wind to pressure and distributed load, two plexiglass patches with 0.02mm thickness have been attached to the top of the designed cellular cores. The air velocity is measured with a barometer, and the total applied force is collected by a load cell located at the fixture clamping the 3D-printed wing. Then, the deformation of the wing tip is captured by a camera, followed by post-processing. For each sample with a height of 2.32 mm (cellular core), the results are obtained for two air velocities (8 and 9 m/s), and each test is repeated three times to confirm the repeatability of the experimental results. For each sample with a height of 3.85 mm (cellular core), four air velocities (9, 10, 11, and 12 m/s) are used. Out-of-plane stiffness under uniform loading is obtained by dividing the recorded force by the tip deflection.

In general pressure on an object in a wind tunnel can be obtained by $P = \frac{1}{2} p_{air} v_{air}^2$; therefore, we can obtain the resultant force applied to the sample by $F = PA_{sample}$. We can relate obtained forces from the load cell to the velocity of the wind. To do so, all velocities and recorded forces are plotted and a quadratic equation is interpolated. Due to the fact that all variables for different designs are the same, forces applied to all samples are also the same for a specific wind velocity. Obtained constant for the interpolation equation is 0.063 kg/m. If we use temperature, humidity, and pressure of air in the lab, we can obtain air density and by multiplication of air density by the sample and fixture area, we get $\frac{1}{2} p_{air} A_{sample} = 0.062 \frac{kg}{m}$, which shows we can assume that pressure is almost uniform over the samples. In this calculation, we have considered the air pressure = 1050 mbar, temperature = 19°, humidity = 33%, which lead to the air density = 1.2488 kg/m^3. The area of the wing and its fixture has been obtained 0.099 m^2. All experimental results of the wind tunnel are presented in Figures S68, S69, and S70.

A.32 Aerodynamic performance of the wing

In wing design, the airfoil determines the pressure profile over the wing surface. Airfoils are designed in a way that creates high pressure under the wing to generate maximum lift and minimum drag forces. In this research, we assumed that the airfoil shape is constant and only the underlying architecture of the core of the wing varies following the dragonfly-inspired approach (Figure S71).

A.33 Direct generation of the form pattern

To compare the generated results from our method and the method that directly generates the form pattern from its boundary, we also trained a GAN model with the same dataset, but only inputting the boundary image and outputting the form image (Figure S72). The result shows that the generated pattern is not continuous and cannot perform structural properties with thickness. Therefore, our method that also generates the force-related information provides a better solution.

Acknowledgements
Our original data was collected from the following resources: the dragonfly wing [1], the grasshopper wing [39], the Amazon water lily [40], and the damselfly wing [17]. The graphic statics implementation was provided by PolyFrame [41]. The GAN model was implemented using PyTorch [42], and the ANN model was implemented using Tensorflow [43].

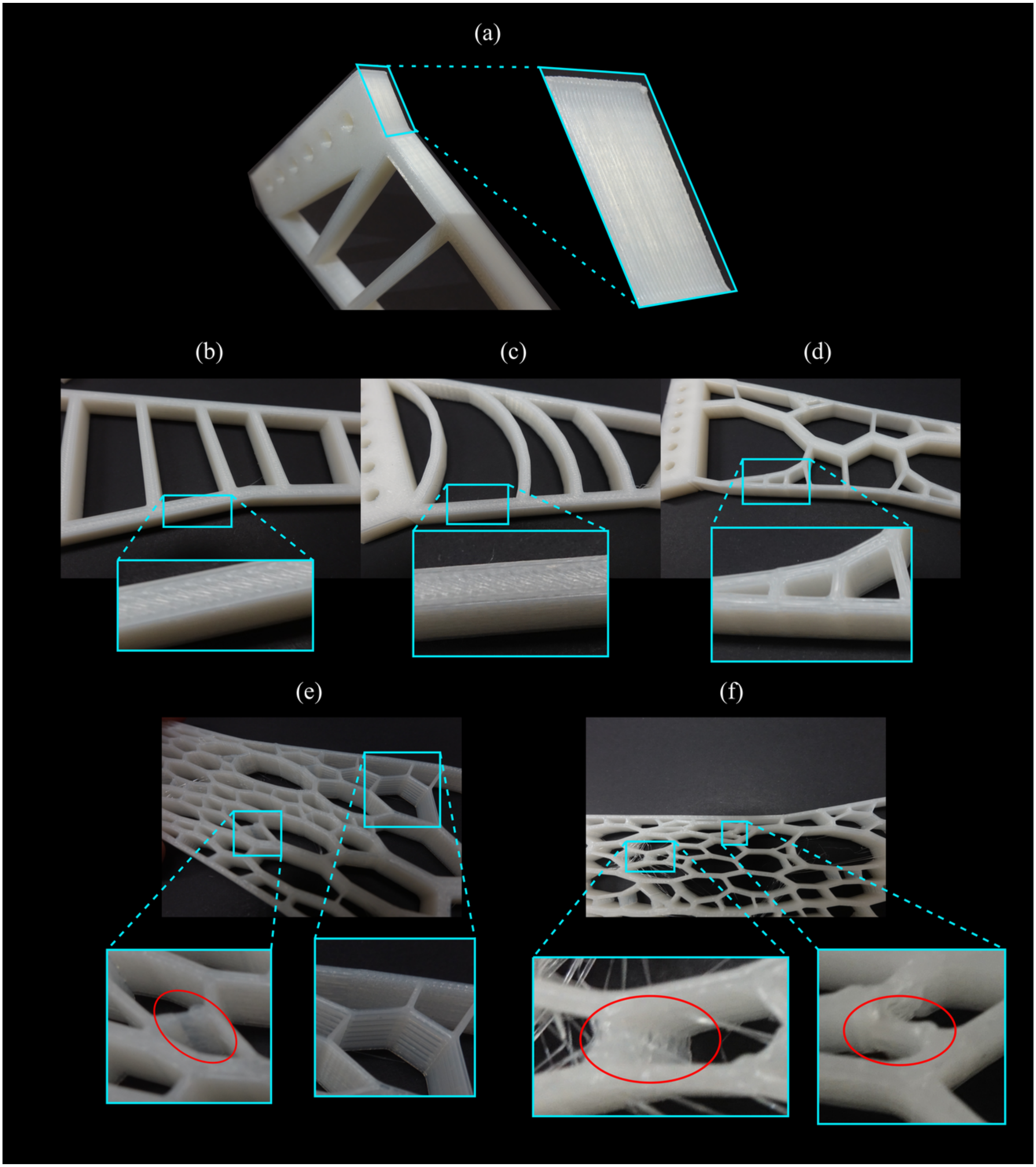

Figure S64: 3D printing quality with FDM: (a) layered structure along the z direction, (b), (c), and (d) show the acceptable quality of 3D printed samples with thick elements, (e) and (f) defects are found in thin struts.

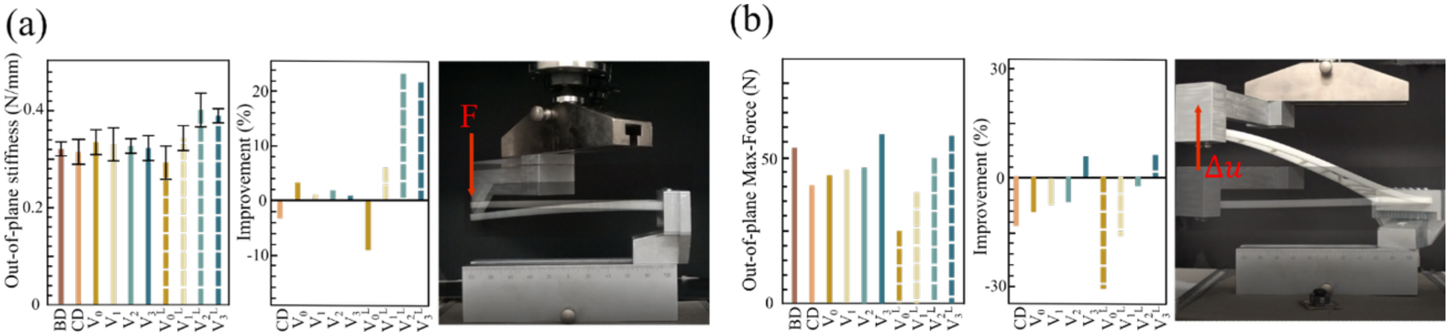

Figure S65: (a) Out-of-plane stiffness properties under an out-of-plane point load and (b) Load bearing ability of the designs under the out-of-plane deformation.

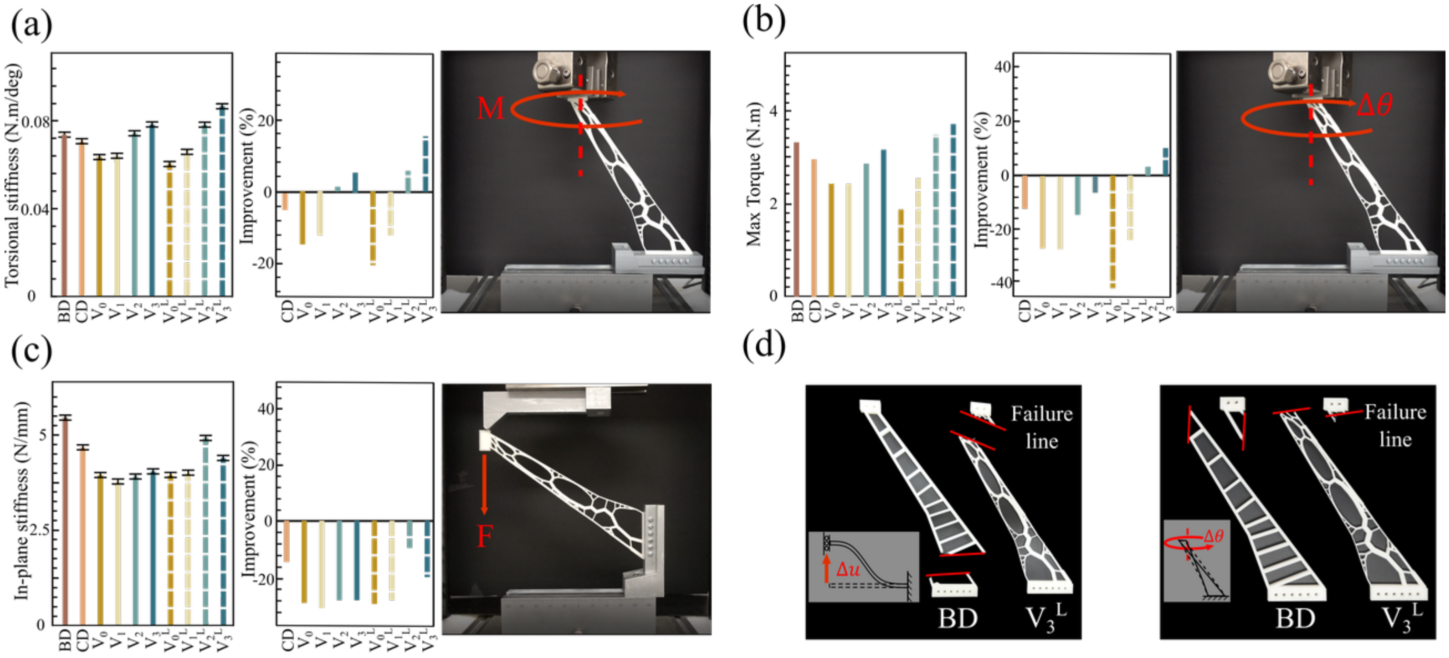

Figure S66: Mechanical properties; stiffness properties under (a) torsional and (c) in-plane point load, (b) load bear-ability of the designs under torsional rotations; and (d) failure section of designs.

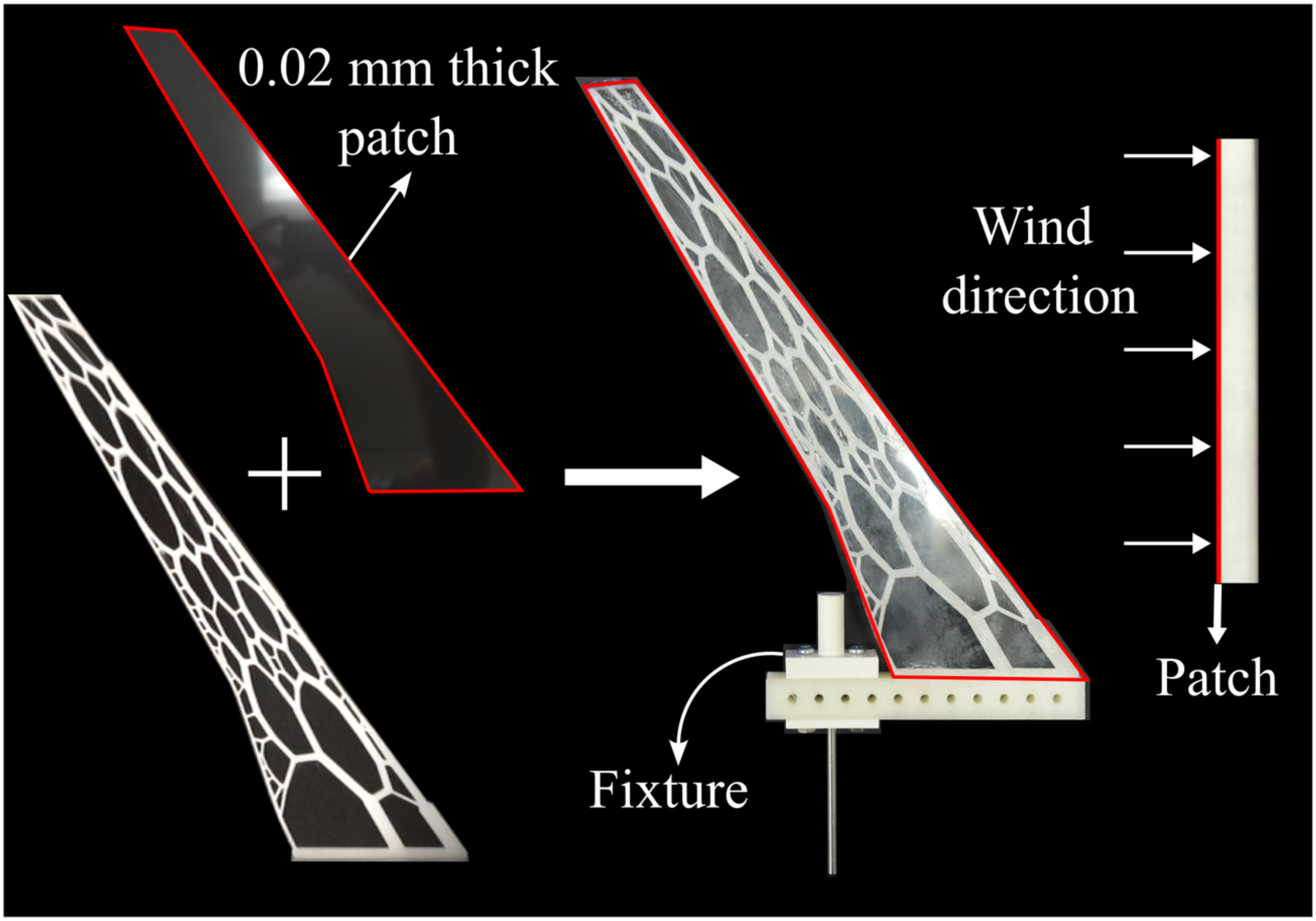

Figure S67: Sample, fixture, and 0.02 mm patch for generating uniform pressure on wing.

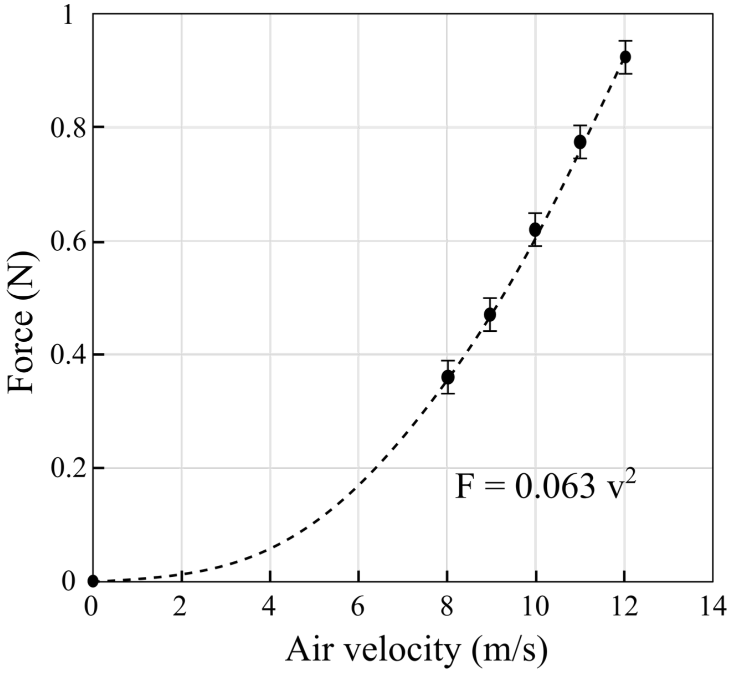

Figure S68: Relation of wind velocity and recorded forces from load cell.

	Record at v=8/m/s			Record at v=9/m/s		
**sample**	Force (N)	Wind speed(m/s)	Defection (mm)	Force (N)	Wind speed(m/s)	Defection (mm)
V_0^L	0.35	8.0	14	0.44	9.0	17.6
V_0	0.4	8.6	15.2	0.52	9.8	18.8
V_1^L	0.34	7.9	12	0.45	9.1	16.1
V_1	0.36	8.1	12.2	0.48	9.4	15.1
V_2^L	0.34	7.9	11.1	0.45	9.1	15.5
V_2	0.38	8.3	11.2	0.49	9.5	14.5
V_3^L	0.34	7.9	10.8	0.47	9.3	15.1
V_3	0.34	7.9	10.9	0.45	9.1	14.3
**VB**	0.38	8.3	13.4	0.49	9.5	17.3
**VR**	0.37	8.2	16.5	0.49	9.5	20.8

Figure S69: Wind tunnel results for wing with h = 2.32 mm.

	v = 9 m/s			v = 10 m/s			v = 11 m/s			v =12 m/s		
**Sample**	F (N)	v (m/s)	D (mm)	F (N)	v (m/s)	D (mm)	F (N)	v (m/s)	D (mm)	F (N)	v (m/s)	D (mm)
V_0^L	0.46	9.0	5.46	0.61	10.1	7.68	0.77	11.0	9.1	0.9	12.0	10.8
V_0	0.48	9.2	5.1	0.62	10.2	6.6	0.77	11.0	7.9	0.91	12.1	9.4
V_1^L	0.47	9.1	5	0.6	10.0	5.9	0.75	10.9	8.4	0.89	11.9	9.8
V_1	0.46	9.0	5.1	0.6	10.0	6.8	0.75	10.9	8.2	0.89	11.9	10.4
V_2^L	0.45	8.9	4.2	0.58	9.8	5.8	0.75	10.9	7.4	0.88	11.9	8.9
V_2	0.48	9.2	4.4	0.61	10.1	5.6	0.78	11.1	7.45	0.91	12.1	9.15
V_3^L	0.45	8.9	4.9	0.61	10.1	6.7	0.78	11.1	8.3	0.91	12.1	9.9
V_3	0.49	9.3	5.2	0.65	10.4	6.4	0.83	11.4	8.1	0.97	12.5	10
**VB**	0.51	9.5	5.9	0.67	10.6	8.4	0.85	11.6	10	0.99	12.6	11.25
**VR**	0.5	9.4	5.5	0.66	10.5	7.5	0.83	11.4	9.35	0.97	12.5	10.9

Figure S70: Wind tunnel results for wing with h = 3.85 mm.

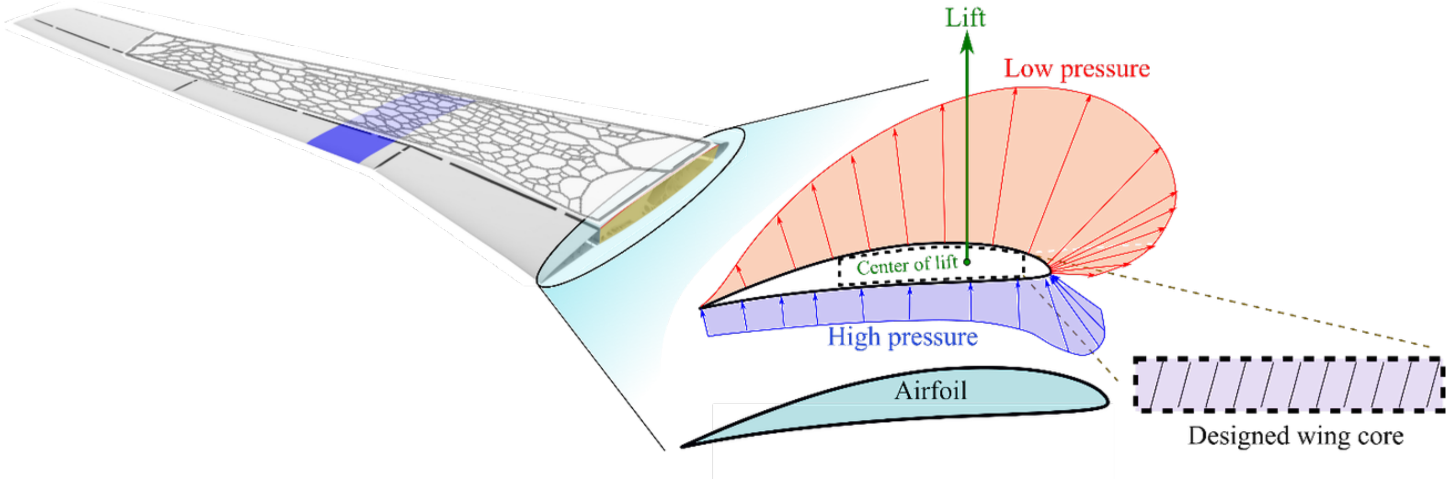

Figure S71: Boeing 777 wing boundary and schematic airfoil and its pressure profile. Wing core position has been shown as a design variable.

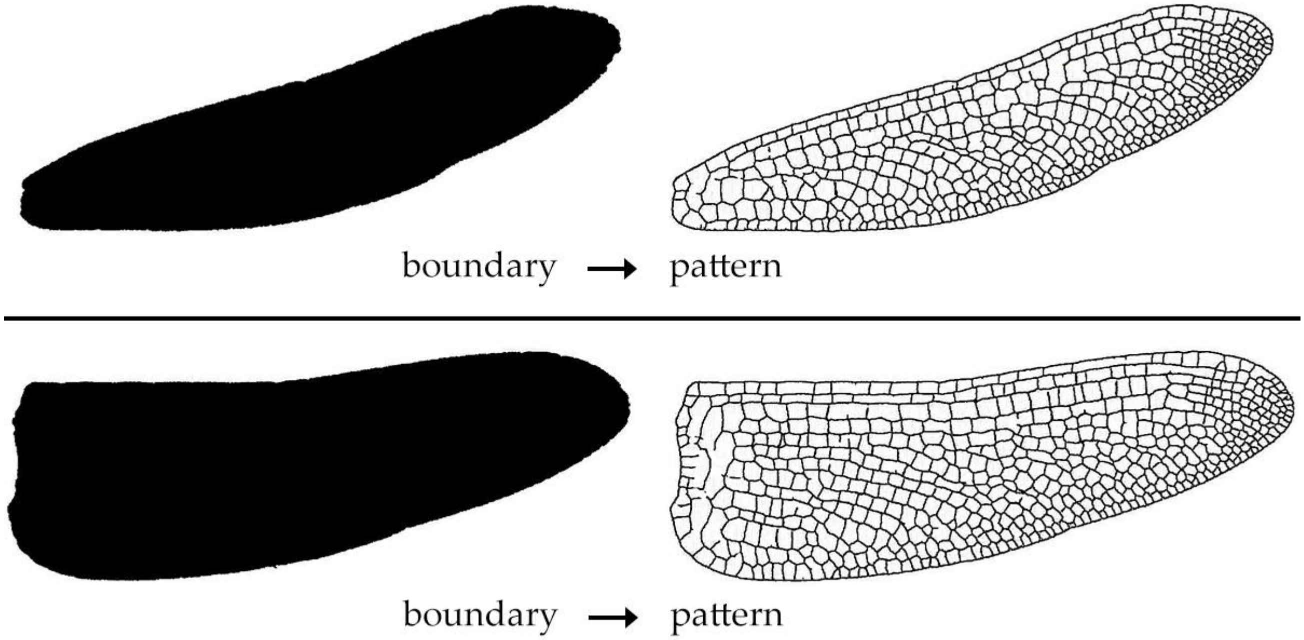

Figure S72: The generated result from the machine learning model that directly learns from the form boundary to the form pattern.

References

[1] John Tann, Dragonfly wings, **2014**, Https://www.flickr.com/photos/31031835@N08.

[2] S. Donoughe, J. D. Crall, R. A. Merz, S. A. Combes, Journal of Morphology **2011**, 272, 12 1409, https://doi.org/10.1002/jmor.10992.

[3] J. Sun, B. Bhushan, Comptes Rendus Mecanique **2012**, 340 3, https://doi.org/10.1016/j.crme.2011.11.003.

[4] F. Johansson, M. Söderquist, F. Bokma, Biological Journal of The Linnean Society **2009**, 97, 2 362.

[5] R. J. Wootton, D. J. Newman, Dragonflies & damselflies. Model organisms for ecological and evolutionary research **2008**, 261 274.

[6] H. Rajabi, A. Shafiei, A. Darvizeh, S. Gorb, Scientific Reports **2016**, 6, 1 39039, https://doi.org/10.1038/srep39039.

[7] S. Fauziyah, C. Alam, R. Soesilohadi, B. Retnoaji, P. Alam, Arthropod structure & development **2014**, 43, 5 415.

[8] E. Appel, L. Heepe, C.-P. Lin, S. N. Gorb, Journal of Anatomy **2015**, 227, 4 561.

[9] K. Culmann, Die Graphische Statik, Verlag Meyer & Zeller, Zürich, **1864**.

[10] R. H. Bow, Economics of construction in relation to framed structures, Spon, London, **1873**.

[11] L. Cremona, Graphical Statics: Two Treatises on the Graphical Calculus and Reciprocal Figures in Graphical Statics, Clarendon Press, Oxford, **1890**.

[12] W. S. Wolfe, Graphical Analysis: A Text Book on Graphic Statics, McGraw-Hill Book Company, Inc., **1921**.

[13] J. C. Maxwell, Philosophical Magazine and Journal Series **1864**, 4, 27 250.

[14] T. Van Mele, P. Block, Computer-Aided Design **2014**, 53 104.

[15] M. Akbarzadeh, M. Hablicsek, In Design Modelling Symposium Berlin. Springer, **2019** 3–17.

[16] A. Nejur, M. Akbarzadeh, Computer-Aided Design **2021**, 134 103003, https://doi.org/10.1016/j.cad.2021.103003.

[17] J. Hoffmann, S. Donoughe, K. Li, M. K. Salcedo, C. H. Rycroft, Proceedings of the National Academy of Sciences **2018**, 115, 40 9905, https://doi.org/10.1073/pnas.1721248115.

[18] P. Isola, J.-Y. Zhu, T. Zhou, A. A. Efros, In Proceedings of the IEEE conference on computer vision and pattern recognition. **2017** 1125–1134.

[19] W. Huang, H. Zheng, In Proceedings of the 38th Annual Conference of the Association for Computer Aided Design in Architecture (ACADIA). **2018** 156–165.

[20] R. Rombach, A. Blattmann, D. Lorenz, P. Esser, B. Ommer, In Proceedings of the IEEE/CVF Conference on Computer Vision and Pattern Recognition. **2022** 10684–10695.

[21] Image-py/sknw: build net work from skeleton image (2d-3d), `https://github.com/Image-Py/sknw`, **2021**, (Accessed on 08/05/2021).

[22] Rhino - rhinoceros 3d, `https://www.rhino3d.com/`, **2021**, (Accessed on 08/05/2021).

[23] H. Zheng, V. Moosavi, M. Akbarzadeh, Automation in Construction **2020**, 119 103346.

[24] H. Zheng, P. F. Yuan, Building and Environment **2021**, 205 108178.

[25] S. Karsoliya, International Journal of Engineering Trends and Technology **2012**, 3, 6 714.

[26] A. Udris, boldmethod **2014**, 6, 30 1.

[27] Javascript.com, https://www.javascript.com/, **2022**, (Accessed on 03/26/2022).

[28] Html canvas, https://www.w3schools.com/html/html5_canvas.asp, **2022**, (Accessed on 03/26/2022).

[29] The web framework for perfectionists with deadlines — django, https://www.djangoproject.com/, **2022**, (Accessed on 03/26/2022).

[30] Mysql, https://www.mysql.com/cn/, **2022**, (Accessed on 03/26/2022).

[31] Grasshopper - algorithmic modeling for rhino, https://www.grasshopper3d.com/, **2022**, (Accessed on 03/03/2022).

[32] Tensorflow, https://www.tensorflow.org/, **2022**, (Accessed on 03/26/2022).

[33] Pytorch, https://pytorch.org/, **2022**, (Accessed on 03/26/2022).

[34] Three.js javascript 3d library, https://threejs.org/, **2022**, (Accessed on 03/26/2022).

[35] Karamba3d – parametric engineering, https://www.karamba3d.com/, **2022**, (Accessed on 04/29/2022).

[36] Wing structure generator — psl, http://www.ai-gs.com/frontend/DFW-GH.html, **2022**, (Accessed on 04/07/2022).

[37] N. Aage, E. Andreassen, B. S. Lazarov, O. Sigmund, Nature **2017**, 550, 7674 84.

[38] R. Martinez-Val, E. Perez, J. F. Palacin, In 43 rd AIAA Aerospace Sciences Meeting and Exhibit. **2005** 2005.

[39] idtools, Grasshoppers of the western u.s., **2021**, Http://idtools.org/id/grasshoppers/gallery.php?page=5.

[40] Google, Google image search, **2021**, Https://www.google.com/.

[41] Polyhedral Structures Laboratory, Polyframe, **2018**, = https://www.food4rhino.com/app/polyframe.

[42] PyTorch, Pytorch, **2021**, Https://pytorch.org/.

[43] tensorflow, tensorflow, **2021**, Https://www.tensorflow.org/.
